# Supplementary material for: Organisation of care for people receiving drug-resistant tuberculosis treatment in South Africa: a mixed methods study
Source: BMJ Open. 2023 Nov 17;13(11):e067121. doi: 10.1136/bmjopen-2022-067121 (PMC10660906; doi:10.1136/bmjopen-2022-067121)
Supplement: Supplementary data [file bmjopen-2022-067121supp002.pdf]

Supplementary material: A

Additional information related to COREQ (Consolidated criteria for Reporting Qualitative research) Checklist

Domain 1: Research team & reflexivity

LD, JH, SLR and LM conducted interviews. The researchers were a team of clinicians and social science degree majors qualified in the fields of medicine, biology and teaching. At the time of the study all were full time researchers on the project. Three were female and one was male. Three were South African and one was Australian. Three had Masters degrees and one had a Bachelor's degree. LD had been employed as a clinician in the DR-TB, HIV and family medicine programs in KZN and the Western Cape and had been previously working on a DR-TB linkage to care study. JH had been employed as a clinician in the DR-TB and infectious diseases domain at Papua New Guinea. SLR had been teaching senior high school students and working in a health care call centre and medical practice previously. LM had been completing a master's degree in biology involving geo-informatics. LD and JH had been investigators in previous DR-TB studies. There was no formal relationship established prior to the study, except some familiarity between the local DR-TB clinicians and LD. The participants were aware of research aims and objectives and the names of the researchers and investigators. The participants were aware that the researchers were interested in health systems around DR-TB disease in SA.

Domain 2: Study design

Questions and guides were piloted with a smaller group of local DR-TB staff at a non-study facility and then provided to participants by the interviewers, together with patient journey maps obtained from that region to serve as probes for some questions. Audio recordings were obtained, except in the case of a correctional services facility where recording was not permitted. A single round of interviews took place at the work place during tea or lunch breaks. Field notes were made during and then more comprehensively after the interview. The duration of the interviews ranged from 30-50 minutes, and depended on the operational work time constraints and the degree of insight and knowledge held and shared by the participant. Data saturation was discussed before ending interviews. Transcripts were not returned for comment or correction.

Domain 3: Analysis and findings

SLR and LD reviewed the interviews and coded the data, using a simple, unstructured coding scheme to extract information on the implications of the patterns of care for patients or the health care system . Participants did not give feedback on the data, except at an end-of-study feedback session with the Departments of Health, which was not used to inform this manuscript. Participant quotations were not presented. There was consistency between the data presented and the findings. Major themes were clearly presented in the findings. The nature of the patient pathway analysis allowed for interrogation of both major themes (common reasons for patient movement) as well as minor themes less common reasons for patient movement).

Dickson L, *et al. BMJ Open* 2023; 13:e067121. doi: 10.1136/bmjopen-2022-067121

Supplementary information: B

		Rural or urban	Demographic and health care descriptors					Poverty indices				Death and disease profile						
			Population count	Population density / km^2	Number of clinics	Number of public hospitals	Provincial spend on health / person / year (ZAR)*	Population below lower poverty line	Unemployed	Informal shack dwellings	Illiteracy	Life expectancy (yrs)	% of deaths due to infectious diseases	Leading cause of natural death	HIV / TB coinfection	TB incidence / 100 000 in 2015	Number of MDR/RR-TB cases in 2017**	
																	XDR	MDR
PROVINCE	KWAZULU-NATAL						R3 527	44%	28%		15%	57-64			69%	685	115	1687
DISTRICT	eThekwini (1)***	City (metro)	3 702 231	1502	31	18		41%	19%	13%	17%		16%	Heart disease		698	85	1063
	uThungulu (2)	City (small)	971 135	88	83	9		72%	34%	3%	12%		21%	TB		859	7	242
	iLembe (3)	Rural (small metro)	657 612	185	36	8		55%	31%	9%	15%		24%	TB		801	10	151
	uMkhanyakude (4)	Rural district	689 090	61	86	5		80%	31%	15%	25%		27%	HIV/ AIDS		577	6	146
	uThukela(5)	Rural district	706 588	63	49	4		63%	29%	1%	10%		24%	TB		533	7	85
PROVINCE	WESTERN CAPE						R3 867	32%	20%		7%	66-71			47%	681	86	1140
DISTRICT	City of Cape Town (6)	City (metro)	4 208 000	1530	130	18		31%	22%	11%	2%		14%	Diabetes		596	59	757
	Eden (7)	City (metro)	611 279	25	209	7		33%	27%	8%	12%		17%	Diabetes		809	5	100
	Cape Winelands (8)	Rural district	917 462	40	134	10		34%	17%	7%			18%	Diabetes		880	14	170
	West Coast (9)	Rural district	436 403	15	180	7		30%	19%	8%	13%		16%	Heart disease		837	8	113
PROVINCE	EASTERN CAPE						R3 046	56%	47%		18%	60-67			56%	692	281	1151
DISTRICT	Buffalo City (7)	City (metro)	893 157	300	103	10		41%	35%	25%	12%		21%	TB		743	77	314
	Nelson Mandela Bay(8)	City (metro)	1 263 051	615	68	12		51%	63%	7%	1%		20%	Diabetes		938	150	471
	OR Tambo (5)	Rural district	1 457 384	108	147	16		67%	39%	1%	14%		20%	TB		571	22	220
	Sarah Baartman (10)	Rural district	520 480	8	89	15		41%	30%	9%	13%		20%	TB		1022	32	146

Table S1. Description of key health care and demographic features of the study districts

* ZAR = South African Rands (1 US dollar is approximately 17 ZAR)

** Data from National Institute of Communicable Diseases

***Reference numbers for data sources are given following each district name

Disease	Definition	Programmatic treatment for MDR/RR-TB in South Africa
DR-TB	Drug resistant TB<br>(Resistance to any TB drug)	Second-line treatment for 18–24 months, with a combination including a combination selection with several of:<br>• Fluoroquinolones, e.g.: ofloxacin, moxifloxacin, levofloxacin<br>• Second-line injectable drugs, e.g.: amikacin, kanamycin, capreomycin<br>• Other available drug, e.g.: ethionamide, terizidone, para-aminosalicyclic acid<br>• Repurposed drugs, e.g.: linezolid, clofazimine<br>• New drugs recently available, e.g.: bedaquiline, delamanid
MDR-TB	Multi drug resistant TB<br>(Resistance to isoniazid and rifampicin)	
RR-TB	Rifampicin resistant TB<br>(Resistant to at least rifampicin, regardless of resistance to other drugs)	
PreXDR-TB	Pre-Extensively drug resistant TB<br>(MDR-TB with resistance to either a fluoroquinolone OR a second-line injectable TB drug)	
XDR-TB	Extensively drug-resistant TB<br>(MDR-TB with resistance to both a fluoroquinolone AND a second-line injectable TB drug)	
Completed treatment	Treatment completed as recommended by the national policy without evidence of failure *but no record that* three or more consecutive cultures ≥30 days apart are negative after the intensive phase.	
Cured	Treatment completed as recommended by the national policy without evidence of failure *and* three or more consecutive cultures ≥30 days apart are negative after the intensive phase.	

Table S2. Definitions and treatment for drug-resistant TB at the time of the study

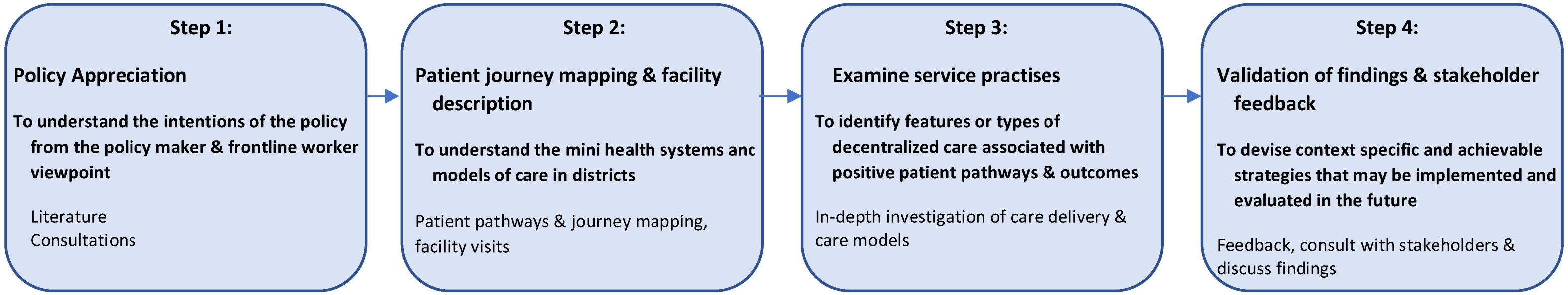

Supplementary Figure 1. The overarching research project, within which this sub-study was nested. This analysis relates to Step 2 and Step 3 of the project.

Supplementary Figure 2: South African map showing thirteen districts selected for study in three provinces. Districts are shaded according to Province (shaded darker green in Western Cape, lighter green in Eastern Cape and orange in KwaZulu-Natal).

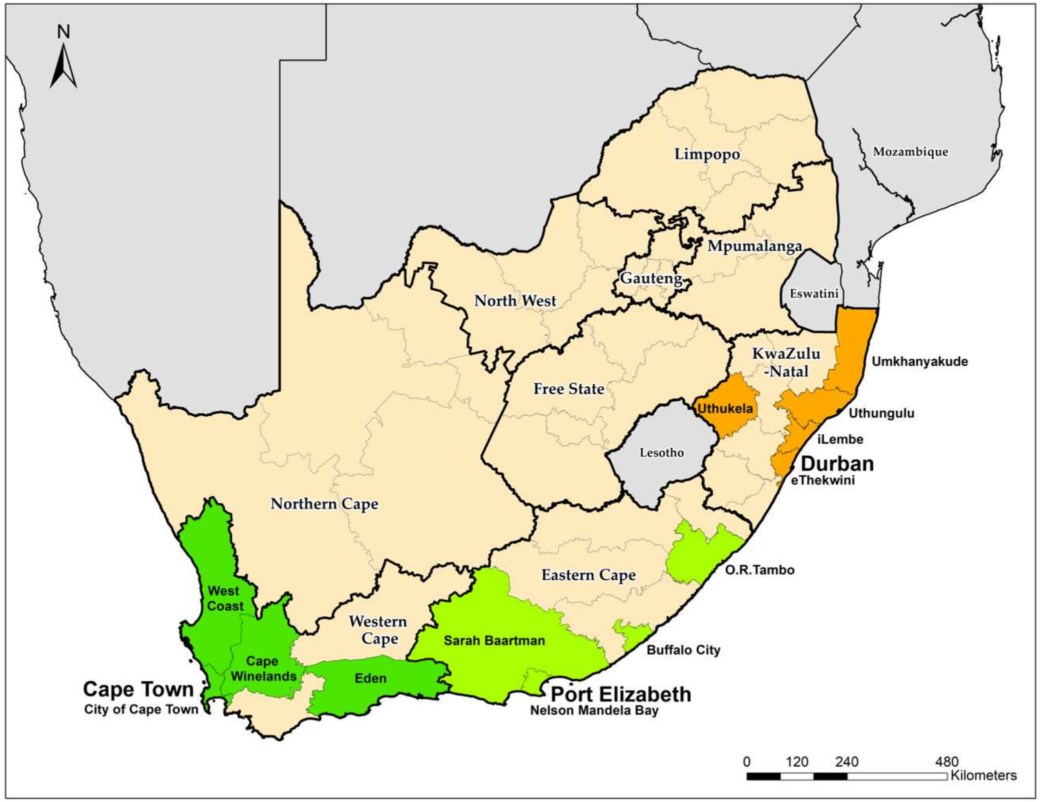

References:

1. National Department of Cooperative Governance & Traditional Affairs South Africa. Profile and Analysis : District Developement Model. 2020. p. 31 Ethekwini Metropolitan KZN. Available from: https://www.cogta.gov.za/ddm/wp-content/uploads/2020/07/Metro-Profile_Ethekwini.pdf
2. National Department of Cooperative Governance & Traditional Affairs South Africa. King Cetshwayo District. 2020.
3. National Department of Cooperative Governance & Traditional Affairs South Africa. Ilembe District Municipality KZN [Internet]. 2020. p. 39. Available from: https://www.cogta.gov.za/ddm/wp-content/uploads/2020/11/Ilembe-September2020.pdf
4. National Department of Cooperative Governance & Traditional Affairs South Africa. Umkhanyakude District Municipality [Internet]. 2020 [cited 2001 Feb 20]. p. 1–24. Available from: https://www.cogta.gov.za/ddm/wp-content/uploads/2020/07/Umkhanyakude-DM-Final-JUNE-2020.pdf
5. National Department of Cooperative Governance & Traditional Affairs South Africa. Uthukela District Municipality EC [Internet]. 2000. p. 34. Available from: https://www.cogta.gov.za/ddm/wp-content/uploads/2020/11/Uthukela-October-2020.pdf
6. National Department of Cooperative Governance & Traditional Affairs. City of Cape town Metropolitan WC. 2020.
7. National Department of Cooperative Governance & Traditional Affairs South Africa. Buffalo City Metro EC [Internet]. 2000. p. 32. Available from: https://www.cogta.gov.za/ddm/wp-content/uploads/2020/08/DistrictProfile_BUFFALOCITY07072020-1.pdf
8. National Department of Cooperative Governance & Traditional Affairs South Africa. NMBM Metro EC [Internet]. 2000. p. 1–30. Available from: https://www.cogta.gov.za/ddm/wp-content/uploads/2020/11/Nelson-Mandela-Bay-Metro-October-2020-1.pdf
9. National Department of Cooperative Governance & Traditional Affairs South Africa. West Coast District Municipality WC [Internet]. 2000 [cited 2022 Jan 20]. Available from: https://www.cogta.gov.za/ddm/wp-content/uploads/2020/08/DistrictProfile_WESTCOAST23072020-002.pdf
10. National Department of Cooperative Governance & Traditional Affairs South Africa. Sarah Baartman District Municipality GAU [Internet]. 2000. p. 43. Available from: https://www.cogta.gov.za/ddm/wp-content/uploads/2020/08/DistrictProfile_SarahBaartman01072020.pdf
