# Supplementary material for: Organisation of care for people receiving drug-resistant tuberculosis treatment in South Africa: a mixed methods study
Source: BMJ Open. 2023 Nov 17;13(11):e067121. doi: 10.1136/bmjopen-2022-067121 (PMC10660906; doi:10.1136/bmjopen-2022-067121)
Supplement: Author's manuscript [file bmjopen-2022-067121.draft_revisions.pdf]

BMJ Open is committed to open peer review. As part of this commitment we make the peer review history of every article we publish publicly available.

When an article is published we post the peer reviewers' comments and the authors' responses online. We also post the versions of the paper that were used during peer review. These are the versions that the peer review comments apply to.

The versions of the paper that follow are the versions that were submitted during the peer review process. They are not the versions of record or the final published versions. They should not be cited or distributed as the published version of this manuscript.

BMJ Open is an open access journal and the full, final, typeset and author-corrected version of record of the manuscript is available on our site with no access controls, subscription charges or pay-per-view fees (http://bmjopen.bmj.com).

If you have any questions on BMJ Open's open peer review process please email info.bmjopen@bmj.com

BMJ Open

A mixed-methods study of organisation of care delivery for patients receiving drug-resistant tuberculosis treatment in South Africa.

Journal:	*BMJ Open*
Manuscript ID	bmjopen-2022-067121
Article Type:	Original research
Date Submitted by the Author:	03-Mar-2023
Complete List of Authors:	Dickson, Lindy; University of Cape Town Faculty of Health Sciences, Department of Pathology<br>Le Roux, Sacha; University of Cape Town Faculty of Health Sciences, Department of Pathology<br>Mitrani, Leila; University of Cape Town<br>Hill, Jeremy; London School of Hygiene &Tropical Medicine , TB Centre<br>Jassat, Waasila; University of the Western Cape School of Public Health<br>Cox, Helen; University of Cape town, Medical Microbiology<br>Mlisana, Koleka; University of KwaZulu-Natal College of Health Sciences, School of Laboratory Medicine and Medical Sciences<br>Black, John; University of Cape Town Faculty of Health Sciences, Division of Infectious Diseases<br>Loveday, Marian; Medical Research Council of South Africa<br>Grant, Alison; London School of Hygiene & Tropical Medicine, TB Centre; Africa Health Research Institute<br>Kielmann, Karina; Institute of Tropical Medicine<br>Ndjeka, Norbert; National Department of Health; University of KwaZulu-Natal<br>Moshabela, Mosa; University of KwaZulu-Natal College of Health Sciences, School of Nursing and Public Health<br>Nicol, Mark; University of Cape Town; University of Western Australia
Keywords:	Tuberculosis < INFECTIOUS DISEASES, Health policy < HEALTH SERVICES ADMINISTRATION & MANAGEMENT, PUBLIC HEALTH, QUALITATIVE RESEARCH

SCHOLARONE™
Manuscripts

I, the Submitting Author has the right to grant and does grant on behalf of all authors of the Work (as defined in the below author licence), an exclusive licence and/or a non-exclusive licence for contributions from authors who are: i) UK Crown employees; ii) where BMJ has agreed a CC-BY licence shall apply, and/or iii) in accordance with the terms applicable for US Federal Government officers or employees acting as part of their official duties; on a worldwide, perpetual, irrevocable, royalty-free basis to BMJ Publishing Group Ltd ("BMJ") its licensees and where the relevant Journal is co-owned by BMJ to the co-owners of the Journal, to publish the Work in this journal and any other BMJ products and to exploit all rights, as set out in our licence.

The Submitting Author accepts and understands that any supply made under these terms is made by BMJ to the Submitting Author unless you are acting as an employee on behalf of your employer or a postgraduate student of an affiliated institution which is paying any applicable article publishing charge ("APC") for Open Access articles. Where the Submitting Author wishes to make the Work available on an Open Access basis (and intends to pay the relevant APC), the terms of reuse of such Open Access shall be governed by a Creative Commons licence – details of these licences and which Creative Commons licence will apply to this Work are set out in our licence referred to above.

Other than as permitted in any relevant BMJ Author's Self Archiving Policies, I confirm this Work has not been accepted for publication elsewhere, is not being considered for publication elsewhere and does not duplicate material already published. I confirm all authors consent to publication of this Work and authorise the granting of this licence.

For peer review only

Title: A mixed-methods study of organisation of care delivery for patients receiving drug-resistant tuberculosis treatment in South Africa.

Corresponding author: Mark Nicol

Email: mark.nicol@uwa.edu.au

Tel: (+61) 410 584 165

Address: School of Biomedical Sciences
University of Western Australia
M504, Perth, WA 6009, Australia

Author names and affiliations:

Lindy Dickson[1], Sacha Le Roux[1], Leila Mitrani[1], Jeremy Hill[3,4], Waasila Jassat[2], Helen Cox[1,5], Koleka Milsana[6], John Black[7,8], Marian Loveday[9,10], Alison D. Grant[11,12,13], Karina Kielmann[14,15], Norbert Ndjeka[16,17], Mosa Moshabela [12,19], Mark P. Nicol[1,18]

1. Division of Medical Microbiology, University of Cape Town, Cape Town, South Africa
2. School of Public Health, University of the Western Cape, Cape Town, South Africa
3. Faculty of Medicine and Health, The University of Sydney, Sydney, New South Wales, Australia
4. Tuberculosis Research Program, Centenary Institute Medical Research Foundation, Newtown, New South Wales Australia
5. Institute for Infectious Disease and Molecular Medicine and Wellcome Centre for Infectious Disease Research in Africa, University of Cape Town, Cape Town, South Africa

For peer review only - http://bmjopen.bmj.com/site/about/guidelines.xhtml

6. School of Laboratory Medicine and Medical Sciences, University of KwaZulu-Natal, Durban, South Africa
7. Department of Infectious Diseases, Livingstone Hospital, Port Elizabeth, South Africa
8. Division of Infectious Diseases, Department of Medicine, University of Cape Town, Cape Town, South Africa
9. HIV and other Infectious Diseases Research Unit, South African Medical Research Council, South Africa
10. Centre for the AIDS Programme of Research in South Africa (CAPRISA), University of KwaZulu-Natal, Durban, South Africa
11. TB Centre, London School of Hygiene & Tropical Medicine, London, UK
12. Africa Health Research Institute, School of Laboratory Medicine and Medical Sciences, College of Health Sciences, University of KwaZulu-Natal, Durban, South Africa
13. School of Public Health, University of the Witwatersrand, Johannesburg, South Africa
14. Institute of International Health and Development, Queen Margaret University, Edinburgh, United Kingdom
15. Equity and Health Unit, Department of Public Health, Institute of Tropical Medicine Antwerp, Antwerp, Belgium
16. National Department of Health, Tuberculosis Control and Management Cluster, Pretoria, South Africa
17. Nelson R Mandela School of Medicine, University of KwaZulu-Natal, Durban, South Africa
18. Marshall Centre, Division of Infection and Immunity, School of Biomedical Sciences, University of Western Australia, Perth, Australia

For peer review only - http://bmjopen.bmj.com/site/about/guidelines.xhtml

19. School of Nursing and Public Health, University of KwaZulu-Natal, Durban, South Africa

Key Words: tuberculosis, drug-resistant, decentralisation, patient pathways, models of care, health care systems, person-centred care, South Africa

For peer review only

For peer review only - http://bmjopen.bmj.com/site/about/guidelines.xhtml

ABSTRACT

Objectives

Treatment for multidrug-resistant/rifampicin-resistant tuberculosis (MDR/RR-TB) is increasingly transitioning from hospital-centred to community-based care. A national policy for decentralised programmatic MDR/RR-TB care was adopted in South Africa in 2011. We aimed to explore variations in the implementation of care models in response this change in policy, and the implications of these variations for patients and the health services.

Methods

A multiple case study methodology was used to study 13 high-burden districts within three provinces. Movements of 191 patients between health care facilities were reconstructed from laboratory records, verified by patient folder reviews, and geospatial patterns were used to identify organisational models. Facility visits and staff and patient interviews were used to confirm the models, identify reasons for patient movement and determine implications of different organisational models for patients and the health system.

Results

Six dominant geospatial movement patterns were identified, which varied in average hospital stay (0-281 days), average patient distance travelled (12-198km) and number of health facilities involved in care (1–5 facilities). More centralized models, which relied on 'Centres of Excellence' for care provision and support, were associated with longer delays to treatment initiation and lengthy hospitalization. Decentralised models facilitated family-centered care and were associated with reduced time to treatment and hospitalization. Flexibility to respond

For peer review only - http://bmjopen.bmj.com/site/about/guidelines.xhtml

to patient needs and health system constraints was achieved through implementation of flexible models, or the implementation of multiple models in a district.

Conclusions

Understanding what models for organising care have evolved may assist policy implementers to tailor implementation to promote particular patterns of care organisation or encourage flexibility, based on patient needs and local health system resources. Our approach can contribute towards the development of a health systems typology for understanding how policy-driven models of service delivery are implemented in the context of variable resources.

Strengths and limitations of this study

- We used real-world patient movement pathways to understand emerging models of care in response to a change in policy.
- We triangulated several different sources of data, including laboratory data, interviews with health care providers and facility visits to understand the implications of these emerging models of care for patients and the health system.
- We could not clearly differentiate to what degree patient needs versus health system organisation and resources determined individual care pathways.
- We were not able to adequately assess care quality indicators, and so are unable to determine whether particular organisational models were associated with favourable patient outcomes.
- We did not interview patients and so cannot draw conclusions about patient preferences.

For peer review only - http://bmjopen.bmj.com/site/about/guidelines.xhtml

BACKGROUND

Tuberculosis (TB) has been the leading cause of death in South Africa for two decades (1) and claims the lives of around 330 persons daily worldwide(2). Multidrug-resistant (MDR-) TB is defined as tuberculosis caused by *Mycobacterium tuberculosis* that is resistant to at least the two key drugs, rifampicin and isoniazid, whilst rifampicin-resistant (RR-) TB is defined by resistance to rifampicin with or without isoniazid resistance. MDR/RR-TB caused illness in around 14 000 persons in South Africa in 2019(3), is more complex and costly to treat than drug sensitive TB(4), and is associated with a fourfold higher mortality (1,5). Within the next 20 years, the MDR/RR-TB case load in South Africa is projected to increase substantially(6,7). Persons with MDR/RR-TB often experience complex medical, psychological(8), social(9,10), and economic(11) challenges. Management for MDR/RR-TB patients requires an intensive multidisciplinary, comprehensive approach, and typically consumes a disproportionate share of health resources(11). Treatment provision requires access to specialised drugs and skilled staff to monitor patients over many months(11). The sustainable use of newer regimens to address this case load will require strengthening MDR/RR-TB treatment delivery systems to ensure effective, person-centred care(12). The WHO End TB Strategy advises a country-specific, tailored, and strategic approach to achieving universal access to high-quality treatment for MDR/RR-TB(13,14).

In response to the rising MDR/RR-TB caseload in South Africa and to local and international pressure from patient and advocacy groups, the National Department of Health (NDoH) adopted an approach to increase ambulatory management of MDR/RR-TB in 2011 (15). This strategy, which had not been widely implemented elsewhere, but was in line with pilot projects

For peer review only

For peer review only - http://bmjopen.bmj.com/site/about/guidelines.xhtml

in South Africa, Peru and Lesotho, was intended to improve treatment access and reduce in-hospital stays(16,17). Operationally, the decentralisation strategy aimed to increase the number of treatment sites from a single dedicated MDR/RR-TB facility per province to several facilities capable of providing MDR/RR-TB care within each district(18). A national guideline for decentralisation of care was published in 2011(19) and updated in 2019(20), and served as a generic, non-prescriptive tool for health managers. Since then, implementation of the policy to decentralize MDR/RR-TB care within South Africa has varied widely (21), in response to differences in health system capacity and readiness to cope with change(22)(23)(22). Funding to support decentralisation was not specifically allocated, which resulted in further setting-specific innovations and adaptations to the program at both provincial and district level(24,25).

We have previously described variation in the care journeys of patients with MDR/RR-TB(21), which may reflect differences in patient co-morbidity or disease features, but likely also reflects regional differences in the organisation of care for the management of MDR/RR-TB. Ideally, models for organization of care should attempt to improve patient outcomes through coordinated care that is person-centred, timely and effective (26); however, models of care often evolve to suit health care providers and health system convenience (27–29).

We therefore studied variation in the patterns of organisation of care for patients with MDR/RR-TB which have emerged in South Africa because of local variation in guideline implementation. We used patient care pathways to identify patterns of care provision, outline

For peer review only - http://bmjopen.bmj.com/site/about/guidelines.xhtml

the implications of these patterns for patients' needs and draw lessons on different ways in which care can be organised for patients with complex illness.

METHODS

The aim of the overarching research project, within which this sub-study was nested, was to identify interventions to optimise decentralisation of services for patients with MDR/RR-TB. The project used a stepwise realist approach to understand the policy context, implementation and working models of decentralisation of MDR/RR-TB care in South Africa. The aim of this sub-study was to explore variations in the implementation of care models in response to a change in MDR/RR-TB health policy, and their implications for patients and the health services.

Study design and setting

We used a multiple case study methodology (30). Data collection included a retrospective cohort study of geographically-linked laboratory records, validated by patient folder reviews. Patterns of patient movement and facility visits were used to understand resource availability and context. Semi-structured interviews with health care workers were undertaken to verify patient journey patterns and organisational patterns and to determine their implications for the health service and patients. We defined MDR/RR-TB as resistance to rifampicin (with or without isoniazid resistance), detected using genotypic or phenotypic methods, irrespective of resistance to other anti-TB drugs.

For peer review only - http://bmjopen.bmj.com/site/about/guidelines.xhtml

Three provinces in South Africa (KwaZulu-Natal [KZN], Eastern Cape [EC] and Western Cape [WC]) were selected due to their high MDR/RR-TB disease burden and differing approaches to decentralised care (Figure 1). Within each province, two urban and two rural districts were selected based on observed differences in patient pathways from the parent study(31). One additional rural district was selected in KZN, due to a rapid shift in urbanisation associated with relocation of the province's airport.

The study took place between July 2016 and July 2019. During this period, MDR/RR-TB treatment regimens were largely based on a standardised aminoglycoside-containing regimen (requiring an injectable drug), tailored according to individualized drug-susceptibility patterns. Prior to the study period, rapid molecular testing for TB and MDR/RR-TB (Xpert MTB/RIF) had been implemented, while limited roll-out of the novel anti-tuberculous drug bedaquiline in pilot programmes and the introduction of a shortened MDR/RR-TB treatment regimen occurred over the study period. Patients generally underwent scheduled monthly clinical evaluation and investigation over the treatment duration, in line with World Health Organization (WHO) guidelines(31).

Selection of patients and assessment of patient movement pathways

We have previously described the methods used to construct MDR/RR-TB patient healthcare journeys(31). Briefly, all patients with a new bacteriologically-confirmed MDR/RR-TB diagnosis were retrospectively sampled from National Health Laboratory Service laboratory records from July to September 2016 (n=2 649). 195 randomly-sampled patients from the study districts were selected and laboratory records served as tracers of patient pathways

For peer review only - http://bmjopen.bmj.com/site/about/guidelines.xhtml

over a 9-month period(31). One patient was found to have been incorrectly diagnosed with MDR/RR-TB, leaving 194 in the analysis. Geographic coordinates of healthcare facilities were collected, and spatial-temporal mapping of patient movement was used to reconstruct patient movement pathways in chronological order for each patient.

Data on patient treatment progress were collected from clinical record reviews, telephonic enquiries with clinics and the electronic MDR/RR-TB registry [EDRWeb] up to two years after diagnosis. Data on patient movement obtained from laboratory records were confirmed by telephonic enquiries at all health care facilities attended by patients. This input was used to verify laboratory information, determine additional patient visits (where no laboratory sample was taken), obtain patient demographic and health data and to identify reasons for movement of patients between facilities. Descriptive statistics for the sampled districts and patients were extracted. Time from laboratory diagnosis to treatment initiation and time on treatment were calculated (32).

Patient movement between health care facilities was geospatially mapped, based on 'as the crow flies' measurements(33). Patterns sharing similar appearance were then grouped according to their pattern and named, as described in other studies(34)(35) (36). Pattern descriptions included the level of facilities visited (ranging from quaternary care level to mobile clinic), movements between facilities, facility admission period or 'period under care' (where facilities retained responsibility for monthly follow up of patients after discharge) and sector of care (state or private). The number of visits (or patient-days) spent under care of a centralized service were used to position the patterns on a decentralisation continuum.

For peer review only - http://bmjopen.bmj.com/site/about/guidelines.xhtml

Detailed assessment of organisation of services for MDR/RR-TB

A total of 22 facilities representing the spectrum of patterns were chosen for more detailed investigation of organisation of MDR/RR-TB care. These facilities represented six urban and seven rural districts as well as the different levels of care ranging from primary health care to tertiary care. At each facility we conducted patient folder reviews and semi-structured interviews with health workers, including pharmacists (n=2), out-patient physicians (n=2), inpatient physicians (n=6), TB facility managers (n=5), ward nurses (n=9), out-patient nurses (n=8) [including HIV care nurses (n=2) and paediatric care nurses (n=2)], and lay health care workers (n=2). Health care workers were selected for interview based on their involvement with the MDR/RR-TB care programme at their facility. No participants refused to participate or dropped out and only the researchers and participant/s were present during interviews. For interviews, specific patient trajectories related to each facility were used as prompts for discussion of the reasons for and implications of patient movements between facilities. Additional data on the same themes were extracted from patient folder reviews. The interviews were conducted in English, with some translation from Afrikaans, and recorded, except in a prison where recording was not permitted. Most interviews were conducted with individuals or pairs of informants, with two interviews involving multidisciplinary panels at a facility. Transcriptions of interviews and folder review data were reviewed by seven researchers who extracted information relating to the implications of the different patterns for care organisation for both patients and the health services. A COREQ checklist is included (Supplementary Material). To further identify the implications of the different organisational patterns for patients and the health services, we used key metrics including time to treatment, length of hospital stay, number of facilities visited, and patient distance travelled.

For peer review only - http://bmjopen.bmj.com/site/about/guidelines.xhtml

Patient and Public Involvement Statement

This study involved neither patients nor members of the public. Dissemination of findings from this study were incorporated into the national MDR/RR-TB meetings organised by the Department of Health and non-governmental organisation partners and presented to programme implementers from all nine provinces of the country.

RESULTS

Description of the provinces and districts where the patient journeys were analysed

We studied urban and rural districts from three high TB burden provinces (Table S1). KwaZulu-Natal is the most populous province, has the lowest life expectancy, the highest MDR/RR-TB incidence, the highest TB/ HIV coinfection rate, and the highest proportion of people with MDR/RR-TB previously treated for TB. The Eastern Cape has highly variable population density between the urban and rural areas, high unemployment, illiteracy and poverty and high levels of resistance to second-line TB drugs in the cities. The Cape Town metropole in the Western Cape is the most populous city of the three study provinces and has the greatest numbers of clinics. The Western Cape has the highest spend on health care per person per year and has the highest life expectancy. It has the lowest overall prevalence of unemployment and poverty (however, with considerable inequity), and diabetes and cardiovascular disease are predominant causes of death.

Description of patient cohort

Of the 194 patients, 62% (n=120) of patients were male and 56% (n=109) were aged 20-39 years. Approximately half of the patients lived in an urban area (54%, n=105) Forty-seven percent (n=91) of the patient cohort were diagnosed with MDR/RR-TB; 16% (n=32) with pre-

For peer review only - http://bmjopen.bmj.com/site/about/guidelines.xhtml

extensively drug-resistant (pre-XDR) and 10% with XDR-TB. Sixty-three percent (n=122) of patients had previously been treated for TB, with 47 % (n=91) previously treated for MDR/RR-TB. 69% (134) of patients were coinfected with HIV at the time of MDR/RR-TB diagnosis (47). Less than half (46%, n=89) had completed some secondary level education, less than a quarter of patients were employed (22%, n=75) and 13% (n=24) reported receiving basic income government grants. Fourteen percent (n=28) had a history of incarceration. Approximately one third of patients (36%, n=69) reported comorbidities other than TB and HIV at diagnosis. All patients were treated exclusively in the state health sector, except for two patients who also received treatment in the private sector. Five percent (n=5) had a household member die from TB disease. We have previously described, in detail, the clinical comorbidities and social needs of patients with MDR/RR-TB in this cohort(47).

The majority (77%, n=150) of patients were diagnosed in an outpatient setting. MDR/RR-TB treatment commenced within a median of 9 days of sample collection, with 58% (n= 112) receiving an aminoglycoside-containing regimen. Bedaquiline was included in 19% (n=38) of regimens. Amongst 164 patients alive at four months after diagnosis, 86% (n=141) were retained in care and amongst 139 patients alive at 6 months after diagnosis, 88% (n=122) were retained in care. At 2 years after diagnosis, 31% of patients (n=60) had completed treatment, 11% (n=21) achieved cure, 22% (n=43) patients had ongoing treatment, 11%(n=22) were lost to follow-up, 23% (n=44) died and 0.5% (n=1) had ongoing disease.

Geospatial patterns of patient movement and their distribution

A total of 191/194 patient movement pathways could be classified into six dominant geospatial movement patterns (Table 1 and Figure 2). These were named according to the trajectories

For peer review only - http://bmjopen.bmj.com/site/about/guidelines.xhtml

of patient movements as 'fan' [n=41 (22%)], 'chicken-foot' [n=19 (10%)], 'hub and spoke' [n=72 (38%)], 'triangle' [n=30 (15%)], 'line' [n=12 (6%)], and 'dot' [n=17 (9%)]. Three patient pathways included too few data points to classify. The patterns were placed on a continuum from the most centralised, 'fan', to the most decentralised, 'dot'. The 'hub and spoke' configuration was the most frequently observed pattern across all three provinces in both urban [n=35 (18%)] and rural [37 (19%)] settings (Table 2).

Description of organisation of care within each of the patterns

We used facility visits, staff interviews and patient record review to describe the organisation of care within each of these patterns. The most centralised pattern, fan, is highly dependent on the designated MDR-TB Centre of Excellence (CoE) to manage patients, following initial assessment at a peripheral facility. CoEs were historically designated by the provincial health departments to manage patients with complex TB. The first step in increasing decentralisation of MDR/RR-TB services was the establishment of specialised MDR-TB hospitals, as a 'step down' from the CoEs. The chicken-foot pattern demonstrates some decentralisation to these specialized hospitals for routine cases, but with dependence on the CoEs for caring for patients with more extensive TB drug resistance or complex presentations. This pattern was common for regions with higher XDR-TB or MDR/RR-TB numbers and where new drugs were being introduced into TB regimens. The hub and spoke pattern, which was predominately observed in rural districts, primarily utilizes specialised MDR-TB hospitals for all care provision, with little capacity existing at primary care level. These three models are considered the more centralised of the six models identified.

For peer review only

The triangle pattern incorporates a more flexible network of MDR-TB services and indicates availability of MDR-TB services at many facilities within the district. Typically, this pattern has oversight by a specialist infectious diseases or TB physician, who is either within the district or closely monitors patient progress through telemedicine. This 'conductor' is responsible for weighing complex social and medical needs and for directing patient movement to an appropriate level of care, according to the clinical need and social circumstances of the patient, at various points during their care. This organisational model is also able to respond to changes in the local availability of MDR-TB resources, by modifying patient referral pathways. This model is frequently associated with decision-making shared between patients and clinicians.

The line and dot patterns reflect a substantial shift to outpatient clinic-based care, frequently including MDR-TB clinical practitioners providing mobile outreach services to different subdistrict primary health care sites. In the line pattern, not all clinics had capacity to initiate treatment, which resulted in some clinics referring to neighbouring clinics or a district hospital for a short admission for treatment initiation. In the dot pattern, clinics had capacity to initiate and maintain MDR/RR-TB treatment independently, with pharmacy and multidisciplinary resources available.

Different patient movement pathways may reflect organisational models within the health system, the incidence of MDR/RR-TB within districts as well as patient features which require delivery of care at a particular level(48). We observed that the distribution of the different movement patterns across districts differed. Most districts had three or four patterns identified

For peer review only - http://bmjopen.bmj.com/site/about/guidelines.xhtml

(Table 2). This suggests flexibility in the organisation of care within these districts to respond to differing patient or health system needs.

We performed exploratory analysis to assess whether patient clinical features were associated with certain patterns of care (Table 3). Children, patients older than 60 years and patients with XDR -TB/ pre-XDR-TB seldom followed the more decentralised line and dot patterns. There was otherwise no evidence that patterns of care differed by patient HIV status, previous TB treatment, low body mass index, or co-morbidities; however, the small sample size precludes robust statistical analysis.

Implications of different patterns of organising care for patients and the health system

We observed patient care pathways, reviewed patient records, visited healthcare facilities, and interviewed facility staff to identify the implications of different organizational patterns on patients and the health system (Table 4). More decentralized models (line and dot) had advantages such as quick linkage to care, shorter hospital stays, and less travel distance for diagnosis and treatment. However, they were at risk of clinical practitioner shortages and less rigorous treatment monitoring. More centralized models (fan and chicken foot) had advantages such as multidisciplinary clinical, pharmacy, radiological, and laboratory resources in one facility, but had disadvantages including long travel distances, treatment initiation delays, and patient travel distance. (Table 1).

For peer review only

DISCUSSION

This study used a novel approach, combining quantitative and qualitative methods and patient pathway analysis, to describe emerging patterns of decentralised care for MDR/RR-TB in South Africa. We identified six patterns of care organisation, ranging from highly centralised to highly decentralised models, with several different models operational in many study districts. The different models demonstrated distinct advantages and disadvantages for both patients and the health system. Although exploratory, the patterns identified can contribute to developing a useful health systems typology for understanding how policy-driven models of service delivery are implemented in the context of variable resources and differing patient needs. Explicit recognition of the divergence of organisational models and cross-district learning around different models for organising care may provide opportunity for health system managers to optimize delivery of care in different contexts, and should be considered by policy developers when planning interventions to decentralize care for complex illness.

Stokes et al have previously described a framework for analysing models of care for patients with multimorbidity (32). A key component of this framework is the organisation of care across different levels of the health system. We, and others, have previously described other elements of this framework in relation to MDR/RR-TB in South Africa, including the theoretical basis for the decentralisation policy (49), the clinical and social needs of the target population(47,50), and the local adaptations of the decentralisation policy(22,51,52). Our approach to identifying organisational models, which involved analysis of patient movement pathways, may be a useful tool and contribute valuable insights into understanding variation in policy implementation. This approach also provides important metrics, including the number of different facilities visited, and travel distances. These metrics differed substantially between

For peer review only - http://bmjopen.bmj.com/site/about/guidelines.xhtml

models in our study, with more centralized models associated with prolonged hospitalization and multiple transfers between facilities. Although patients with MDR/RR-TB who are treated with adequate doses of appropriate drugs rapidly become non-infectious(53), there is increasing recognition that multiple patient transfers (often together with non-TB patients) and prolonged hospitalization may expose both patients and others to unnecessary infectious risks, reduce retention in care and incur financial costs to patients(54,55). Studies in LMICs have shown that physical proximity of healthcare services can play an important role in the use of healthcare facilities(56–58).

Flexible adaptations to healthcare policies that can be deployed at different levels of the health system in response to patient needs can improve the delivery of patient care (20,22,59,60). For example, a flexible referral model like 'triangle', which utilizes centralized clinical oversight to direct patient care, can provide greater patient care flexibility. Similarly, in the Western Cape, implementation of multiple healthcare models allowed for a more seamless patient flow in response to patient needs. Such flexible healthcare models, that can be organized in response to operational constraints and patient needs, may lead to contextually sensitive and optimal patient care that is safe, effective, patient-centered, timely, efficient, and equitable (61–63).

MDR/RR-TB management is complex, due to operational complexities, such as drug supply and treatment monitoring as well as complexities related to psychosocial needs and patient multimorbidity, such as HIV infection, or requirement for specialised services. Patients in this cohort presented with multiple medical comorbidities, including mental illness, and challenging

For peer review only

For peer review only - http://bmjopen.bmj.com/site/about/guidelines.xhtml

social circumstances(47). Our exploration of the advantages and disadvantages of the different organisational patterns highlights the trade-offs when having to consider patients with complex or severe disease. Since it is not possible to decentralise all components of care for people with complex MDR/RR-TB, flexibility to provide differentiated or individualised care, which adapts to address the specific requirements of individuals or a subgroup of patients, is particularly relevant (64). Care integration, and models that aim to overcome fragmentation between providers have been identified as important(65). Ideally, a bio-psychosocial systems approach should be used, where the primary focus of health care is the client in the context of their family(66) and where adult and paediatric services are provided together in a single setting(67). Reviews of family-centred care models in HIV care, indicate excellent adherence, retention in care, and low mortality and/or loss to follow-up in both adult and child services(68). The dot model, where services are provided closest to the community is likely to be best suited to this approach, but may be less ideal where patients home social circumstances are challenging or where patients require highly specialized care for complex disease (69). In the latter circumstances, models, such as triangle, which are intrinsically more flexible, and rapidly responsive to changing patient needs may be more appropriate. Overall, where resources permit, having multiple or flexible models may be desirable in order to respond to medical complexity and enhance person-centred care.

Societal features, such as urbanization, may have driven the emergence of certain models. However, we found no clear association between care patterns and epidemiological features of districts. For example, the most decentralised patterns (line and dot) were seen both in highly urbanized districts with large numbers of cases as well as in sparsely populated rural areas. These models appeared to be responses to differing pressures – the need to

For peer review only - http://bmjopen.bmj.com/site/about/guidelines.xhtml

commence large numbers of patients on treatment in urban areas, and the need to deliver care closer to patients in remote areas. In rural districts in the Eastern Cape and KZN, where several of the more decentralised models were available, these appeared to be driven by clinician champions, as we have previously described(69). In the Western Cape, the pre-existing strength of the primary health care system may have facilitated the preponderance of line and dot models.

The WHO annual TB report card on South Africa cautions that reported numbers of people diagnosed and commenced on TB treatment in 2020 had fallen by 25% compared to the same period in 2019 and that gains in the TB program made over the past decade could be reversed by the COVID-19 pandemic(70). Countries have been encouraged to mitigate COVID-19 related impacts on MDR/RR-TB services by expanding use of digital technologies for remote advice and support, improving community connectivity(71), reducing the need for visits to health care facilities, encouraging home based treatment and increasing use of strategies for facilitating patient drug supply. Lessons from the COVID-19 pandemic may offer lessons for organisation of MDR/RR-TB care delivery, in particular consideration of opportunities to improve connectivity to enhance decentralised care. We identified several examples where either telemedicine had been used, in line or dot models, or physicians travelled to enable peripheral facilities to initiate and care for newly-diagnosed or more complex patients. In some instances, medication prescriptions or clinical records travelled in place of patients. However, these adaptations were not a formal system response but rather an example of local adaptation, often with staff using their own devices for communication(22). Description of patient movement patterns, critical appraisal of reasons for patient movement, and identification of which travel might be avoided through other equally effective means, such as

For peer review only - http://bmjopen.bmj.com/site/about/guidelines.xhtml

virtual consultations, may be a useful exercise for diseases that are complex, rare or require specialized care (72–74).

A limitation of our study is that it is not possible to clearly differentiate to what degree patient needs versus health system organisation and resources determined individual care pathways, but both are likely to have played a role. The substantial differences in care organisation, regardless of patient needs, reflect that there were multiple factors that were likely to determine care organisation. We were not able to adequately assess care quality indicators, and so are unable to determine whether particular organisational models were associated with favourable patient outcomes. Further, we did not interview patients and so cannot draw conclusions about patient preferences. Patients who did not link to care, patients who died shortly after linking to care, and patients linking through the private sector were not included in this study and are all important groups.

In this analysis, we also did not specifically interrogate the reasons for the emergence of the different organisational models. Models of care are often based on what was in place historically, and over time become less responsive to the changing needs of the patient and to health system constraints(75). Models of care are also often developed to bridge service delivery gaps rather than as a planned strategic response to an identified local need (76,77). The development of models of care is commonly an iterative process(78,79), shaped by socio-political, economic, cultural, environmental, and legal drivers (80,81).

For peer review only - http://bmjopen.bmj.com/site/about/guidelines.xhtml

CONCLUSION

With the challenge of striving towards the WHO targets of zero deaths, disease and suffering due to TB by 2035 (82), stronger and more flexible health systems for decentralising patient management of MDR/RR-TB will be required to accommodate varying treatment needs. The study of patient care pathways is a useful approach to obtain insight into the way care is organised for complex conditions. Having more clearly defined and articulated models of care may assist policy planners and implementers to identify which organisational models are most responsive to patient needs, quality indicators and system constraints. Increased use of digital technologies should be explored to democratize specialised and individualized care for MDR/RR-TB and other complex illnesses.

DECLARATIONS

Acknowledgements

This study was funded by a Health Systems Research Initiative award from the Medical Research Council of the United Kingdom and the Wellcome Trust (MR/N015924/1). This UK funded award is part of the EDCTP2 program supported by the European Union. HC is supported by a Wellcome Trust Fellowship. MN is supported by an Australian National Health and Medical Research Council Award (APP1174455). No conflicts of interest were reported.

The authors wish to thank and acknowledge Dr. Norbert Ndjeka (SA NDOH), the provinces of the W Cape, E Cape, KZN the staff at the NHLS and all the facilities, staff and patients for their tremendous input and assistance. We give special mention to the late Dr Iqbal Masters

For peer review only - http://bmjopen.bmj.com/site/about/guidelines.xhtml

and Mrs Anna Maria Evans for their contributions to the study. We also acknowledge the support of S/N Cheryl Liedeman and Dr Widaad Zemanay.

Competing interests:

The authors declare that they have no competing interests.

Funding

This study was supported by a Health Systems Research Initiative award from the Medical Research Council of the United Kingdom and the Wellcome Trust (MR/N015924/1). This UK funded award is part of the EDCTP2 program supported by the European Union. HC is supported by a Wellcome Trust Fellowship. MN is supported by an Australian National Health and Medical Research Council Award (APP1174455).

Authors' contributions

MN, HC, MM, AG, LD and KK designed the overarching study. LD, SL, JH and LM conducted quantitative data collection during phase one of the study. LD, SL, and LM were responsible for qualitative data collection in phase three. LD and MM were involved in conceptualising the manuscript. LD was responsible for drafting the manuscript while MN drafted the manuscript revision. All authors provided input into ongoing versions of the manuscript and approved the final version.

Ethics approval and consent to participate

The study was a retrospective laboratory and record review of routinely captured data and a waiver of informed consent was granted to retrospectively review these records. Participants

For peer review only - http://bmjopen.bmj.com/site/about/guidelines.xhtml

did not provide consent to participate in the study as all data analysed were collected as part of routine diagnosis and care. This study was approved by ethics boards at the University of Cape Town (350/2016) and the London School of Hygiene & Tropical Medicine (11680). Research approval was obtained in each province (Eastern Cape 2016RP30-232, KwaZulu-Natal 2016RP51-466, Western Cape 2016RP45-978).

Consent for publication

Not applicable

Availability of data and materials:

The dataset (anonymised participant data) used and analysed during the current study are not publicly available but can be obtained from the corresponding author (ORCID identifier 0000-0002-0020-1414) on reasonable request and may be shared through a controlled access repository.

Word count: 4326

For peer review only - http://bmjopen.bmj.com/site/about/guidelines.xhtml

Figure titles and legends:

Figure 1: South African map showing thirteen districts selected for study in three provinces. Districts are shaded according to Province (shaded darker green in Western Cape, lighter green in Eastern Cape and orange in KwaZulu-Natal).

Figure 2. Stylised examples of the dominant geospatial movement patterns annotated on a study district. Arrows represent individual patient movements between health care facilities. A: Triangle; B: Chicken Foot; C; Hub and Spoke; D: Dot; E: Line; F: Fan.

References

1. Loveday M, Mzobe YN, Pillay Y, Barron P. Figures of the dead: A decade of tuberculosis mortality registrations in South Africa. S Afr Med J. 2019;109(10):728–32.
2. Statistics South Africa. Mortality and causes of death in South Africa, 2016: Findings from death notification. Stat release [Internet]. 2016;(November 2016):1–127. Available from: https://www.statssa.gov.za/publications/P03093/P030932016.pdf
3. World Health Organization; Global tuberculosis report 2020 [Internet]. Geneva; 2020 [cited 2020 Jun 1]. Available from: https://www.who.int/teams/global-tuberculosis-programme/tb-reports/global-tuberculosis-report-2020
4. Murdoch J, Curran R, Rensburg AJ Van, Awotiwon A, Dube A, Bachmann M, et al. Identifying contextual determinants of problems in tuberculosis care provision in South Africa : a theory - generating case study. Infect Dis Poverty [Internet]. 2021;1–13. Available from: https://doi.org/10.1186/s40249-021-00840-5
5. Sineke T, Evans D, Schnippel K, Van Aswegen H, Berhanu R, Musakwa N, et al. The impact of adverse events on health-related quality of life among patients receiving

For peer review only

treatment for drug-resistant tuberculosis in Johannesburg, South Africa. Health Qual Life Outcomes. 2019;17(1).

6. World Health Organization. Consolidated Guidelines on Tuberculosis Treatment. World Health Organization; 2016. 99 p.

7. World Health Organization. Companion handbook [Internet]. World Health Organization; 2014. 464 p. Available from: https://apps.who.int/iris/bitstream/handle/10665/130918/9789241548809_eng.pdf

8. IOM (Institute of Medicine). Patient Safety: Achieving a NewStandard for Care. Vol. 12, The National Academies Press. Washinton,DC; 2004. 126–129 p.

9. Law S, Daftary A, O'Donnell M, Padayatchi N, Calzavara L, Menzies D. Interventions to improve retention-in-care and treatment adherence among patients with drug-resistant tuberculosis: A systematic review. Eur Respir J. 2019;53(1):13993003.

10. Mohr E, Hughes J, Snyman L, Beko B, Harmans X, Caldwell J, et al. Healthcare delivery: Patient support interventions to improve adherence to drug-resistant tuberculosis treatment: A counselling toolkit. South African Med J. 2015;105(8):631–4.

11. Hochman M, Asch SM. Disruptive Models in Primary Care: Caring for High-Needs, High-Cost Populations. J Gen Intern Med. 2017;32(4):392–7.

12. ReAct Group. Learning from Experiences : Introducing Bedaquiline in South Africa Executive summary [Internet]. 2020 [cited 2021 Nov 18]. Available from: https://www.reactgroup.org/news-and-views/news-and-opinions/year-2020/learning-from-bedaquiline-in-south-africa-comprehensive-health-systems-for-new-antibiotics/

13. Gray RM. The paradigms global plan of TB. Archetypal Explor. 2020;108–28.

14. Stop TB Partnership. The Global Plan to End TB 2016-2020: The Paradigm Shift. World Heal Organ Doc [Internet]. 2015;124. Available from:

For peer review only

For peer review only - http://bmjopen.bmj.com/site/about/guidelines.xhtml

www.stoptb.org/global/plan/plan2/annexes.asp

15. Overland L, Uidelines C. Understanding the process to develop a Model of Care An ACI Framework. Perspect Swallowing Swallowing Disord. 2009;20(3):60–4.

16. Loveday M, Wallengren K, Voce A, Margot B, Reddy T, Master I et al. Comparing early treatment outcomes of MDR-TB in decentralised and centralised settings in KwaZulu-Natal, South Africa. Int J Tuberc Lung Dis. 2012;16(2):209–15.

17. Padayatchi N, Friedland G. Decentralised management of drug-resistant tuberculosis (MDR- and XDR-TB) in South Africa: An alternative model of care. Int J Tuberc Lung Dis. 2008;12(8):978–80.

18. World Health Organization. Guidelines for the programmatic management of drug-resistant tuberculosis Guidelines for the programmatic management of drug-resistant tuberculosis. World Heal Organ. 2008;12–185.

19. National Department of Health South Africa. Multi-drug resistant tuberculosis: A policy framework on decentralised and deinstitutionalised management for South Africa. Pretoria; 2011.

20. National Department of Health South Africa. Multi-drug resistant tuberculosis a policy framework on decentralised and deinstitutionalised management for south africa. 2019.

21. Cox, HS; Dickson-Hall, L; Jassat W et al. Drug-resistant tuberculosis in South Africa: history, progress and opportunities for achieving universal access to diagnosis and effective treatment. In: South African Health Review 2017. 2017. p. 157–67.

22. Kielmann K, Dickson-Hall L, Jassat W et al. “We had to manage what we had on hand, in whatever way we could”: Adaptive responses in policy for decentralized drug-resistant tuberculosis care in South Africa. Health Policy Plan. 2021;36(3):249–59.

For peer review only

23. Kielmann K, Dickson-Hall L, Jassat W, Le Roux S, Moshabela M, Cox H, et al. "We had to manage what we had on hand, in whatever way we could": Adaptive responses in policy for decentralized drug-resistant tuberculosis care in South Africa. Health Policy Plan. 2021;36(3):249–59.

24. WHO. Implementing The End TB Strategy. Antimicrob Agents Chemother [Internet]. 2015;58(12):7250–7. Available from: https://www.cambridge.org/core/product/identifier/CBO9781107415324A009/type/book_part%0Ahttp://arxiv.org/abs/1011.1669%0Ahttp://dx.doi.org/10.1088/1751-8113/44/8/085201%0Ahttp://www.ncbi.nlm.nih.gov/pubmed/25246403%0Ahttp://www.pubmedcentral.nih.gov/artic

25. The Global Fund. Best Practices on TB Case Finding and Treatment. Glob fund org [Internet]. 2018;73. Available from: https://www.theglobalfund.org/Media/8273/Core_Wca-

26. Cott CA, Davis AM, Badley EM, Wong R, Canizares M, Li LC, et al. Commonalities and differences in the implementation of models of care for arthritis: Key informant interviews from Canada. BMC Health Serv Res [Internet]. 2016;16(1):1–12. Available from: http://dx.doi.org/10.1186/s12913-016-1634-9

27. World Health Organization; Everybody's business strengthening health systems to improve health outcomes [Internet]. Geneva; 2007. Available from: https://apps.who.int/iris/bitstream/handle/10665/43918/9789241596077_eng.pdf

28. Saha, S, MD, Beach, MC, Cooper L. Patient Centeredness, Cultural Competence and Healthcare Quality. 2010;100(11):1275–85.

29. National Department of Health South Africa. White paper for the transformation of the health system in South Africa. 1997.

For peer review only - http://bmjopen.bmj.com/site/about/guidelines.xhtml

30. Walt G, Shiffman J, Schneider H, Murray SF, Brugha R, Gilson L. “Doing” health policy analysis: Methodological and conceptual reflections and challenges. Health Policy Plan. 2008;23(5):308–17.

31. Hill J, Dickson-Hall L, Grant AD, Grundy C, Black J, Kielmann K, et al. Drug-resistant tuberculosis patient care journeys in South Africa: A pilot study using routine laboratory data. Int J Tuberc Lung Dis. 2020;24(1):83–91.

32. Stokes J, Man MS, Guthrie B, Mercer SW, Salisbury C, Bower P. The foundations framework for developing and reporting new models of care for multimorbidity. Ann Fam Med. 2017;15(6):570–7.

33. Sijm-Eeken, M, Zheng J, Peute L. Towards a Lean Process for Patient Journey Mapping - A Case Study in a Large Academic Setting. Stud Heal Technol Inf. 2020;270:1071–5.

34. Hanson CL, Osberg M, Brown J, Durham G, Chin DP. Conducting Patient-Pathway Analysis to Inform Programming of Tuberculosis Services: Methods. J Infect Dis. 2017;216(Suppl 7):S679–85.

35. Rathi P, Shringarpure K, Unnikrishnan B, Pandey A, Nair A. Patient treatment pathways of multidrug-resistant tuberculosis cases in coastal South India: Road to a drug resistant tuberculosis center. F1000Research [Internet]. 2019;8:498. Available from: https://doi.org/10.12688/f1000research.17743.5

36. Loveday M, Wallengren K, Reddy T, Besada D, Brust JCM, Voce A, et al. MDR-TB patients in KwaZulu-Natal, South Africa: Cost-effectiveness of 5 models of care. PLoS One [Internet]. 2018;13(4):1–13. Available from: https://dx.plos.org/10.1371/journal.pone.0196003

37. National Department of Cooperative Governance & Traditional Affairs South Africa.

For peer review only

Ethekwini Metropolitan KZN [Internet]. Profile and Analysis : District Developement Model. 2020. p. 31. Available from: https://www.cogta.gov.za/ddm/wp-content/uploads/2020/07/Metro-Profile_Ethekwini.pdf

38. National Department of Cooperative Governance & Traditional Affairs South Africa. King Cetshwayo District [Internet]. 2020. Available from: http://www.health.gov.za/DHP/docs/DHP2018-21/kwazulu_Natal/KCD_DHP_2018.19M.pdf

39. National Department of Cooperative Governance & Traditional Affairs South Africa. Ilembe District Municipality KZN [Internet]. 2020. p. 39. Available from: https://www.cogta.gov.za/ddm/wp-content/uploads/2020/11/Ilembe-September2020.pdf

40. National Department of Cooperative Governance & Traditional Affairs South Africa. Umkhanyakude District Municipality [Internet]. 2020 [cited 2001 Feb 20]. p. 1–24. Available from: https://www.cogta.gov.za/ddm/wp-content/uploads/2020/07/Umkhanyakude-DM-Final-JUNE-2020.pdf

41. National Department of Cooperative Governance & Traditional Affairs South Africa. Uthukela District Municipality EC [Internet]. 2000. p. 34. Available from: https://www.cogta.gov.za/ddm/wp-content/uploads/2020/11/Uthukela-October-2020.pdf

42. National Department of Cooperative Governance & Traditional Affairs. City of Cape town Metropolitan WC [Internet]. 2020. Available from: https://www.cogta.gov.za/ddm/wp-content/uploads/2020/11/City-of-CT-September-2020.pdf

43. National Department of Cooperative Governance & Traditional Affairs South Africa.

For peer review only - http://bmjopen.bmj.com/site/about/guidelines.xhtml

Buffalo City Metro EC [Internet]. 2000. p. 32. Available from: https://www.cogta.gov.za/ddm/wp-content/uploads/2020/08/DistrictProfile_BUFFALOCITY07072020-1.pdf

44. National Department of Cooperative Governance & Traditional Affairs South Africa. NMBM Metro EC [Internet]. 2000. p. 1–30. Available from: https://www.cogta.gov.za/ddm/wp-content/uploads/2020/11/Nelson-Mandela-Bay-Metro-October-2020-1.pdf

45. National Department of Cooperative Governance & Traditional Affairs South Africa. West Coast District Municipality WC [Internet]. 2000 [cited 2022 Jan 20]. Available from: https://www.cogta.gov.za/ddm/wp-content/uploads/2020/08/DistrictProfile_WESTCOAST23072020-002.pdf

46. National Department of Cooperative Governance & Traditional Affairs South Africa. Sarah Baartman District Municipality GAU [Internet]. 2000. p. 43. Available from: https://www.cogta.gov.za/ddm/wp-content/uploads/2020/08/DistrictProfile_SarahBaartman01072020.pdf

47. Mitrani L, Roux S Le, Hill J, Loveday M, Grant AD, Kielmann K, et al. Diverse clinical and social circumstances: developing patient-centred care for DR-TB patients in South Africa L. Public Heal Action. 2021;I(3):120–5.

48. Citro B, Soltan V, Malar J, Katlholo T, Smyth C, Sari AH, et al. Building the Evidence for a Rights-Based , People- Centered , Gender-Transformative Tuberculosis Response : An Analysis of the Stop TB Partnership Community , Rights , and Gender Tuberculosis Assessment. Heal Hum Rights J. 2021;23(2):253–67.

49. Moshabela, M, Cox, H., Jassat, W., Dickson-Hall, L., Kielmann, K., Loveday M., Black, J., Mlisana, K., Nicol, M., Grant A. “We were caught sleeping”: Tensions

For peer review only

For peer review only - http://bmjopen.bmj.com/site/about/guidelines.xhtml

between 'control-based' and 'access-based' approaches in the formulation of a national policy to decentralise management of drug-resistant tuberculosis in South Africa. Manuscr under Rev BMJ Glob Heal.

50. Furin J, Loveday M, Hlangu S, Dickson-hall L, Le Roux S, Nicol M, et al. " A very humiliating illness " : a qualitative study of patient-centered Care for Rifampicin-Resistant Tuberculosis in South Africa. 2020;1–11.

51. Loveday M, Wallengren K, Brust J, Roberts J, Voce A, Margot B et al. Community-based care vs. centralised hospitalisation for MDR-TB patients, KwaZulu-Natal, South Africa. Int J Tuberc Lung Dis. 2015;19(2):163–71.

52. Loveday M, Wallengren K, Voce A, Margot B, Reddy T, Master I, et al. Comparing early treatment outcomes of MDR-TB in decentralised and centralised settings in KwaZulu-Natal, South Africa. Int J Tuberc Lung Dis. 2012;16(2):209–15.

53. Petersen E, Khamis F, Battista G, Glerup J, Marais B, Wejse C, et al. International Journal of Infectious Diseases De-isolation of patients with pulmonary tuberculosis after start of treatment — clear , unequivocal guidelines are missing. Int J Infect Dis [Internet]. 2017;56:34–8. Available from: http://dx.doi.org/10.1016/j.ijid.2017.01.029

54. Shringarpure KS, Isaakidis P, Sagili KD, Baxi RK, Das M, Daftary A. "When treatment is more challenging than the disease": A qualitative study of MDR-TB patient retention. PLoS One. 2016;11(3):1–12.

55. Daftary A, Mondal S, Zelnick J, Friedland G, Seepamore B, Boodhram R, et al. Dynamic needs and challenges of people with drug-resistant tuberculosis and HIV in South Africa : a qualitative study. Lancet Glob Heal [Internet]. 2015;9(4):e479–88. Available from: http://dx.doi.org/10.1016/S2214-109X(20)30548-9

56. Al-mandhari A, Al-adawi S, Al-zakwani I, Al-shafaee M, Eloul L. Impact of

For peer review only

Geographical Proximity on Health Care Seeking Behaviour in Northern Oman. 2008;8(3):310–8.

57. Peabody, JW, Taguiwalo, MM, Robalino, DA, Frenk J. Improving the Quality of Care in Developing Countries. In: Disease Control Priorities in Developing Countries. 2nd ed. New York: Oxford University Press; 2006.

58. Mangundu M, Roets L, Rensberg EJ Van, Africa S, Mangundu M. Accessibility of healthcare in rural Zimbabwe : The perspective of nurses and healthcare users. :1–7.

59. Benn J, Burnett S, Parand A, Pinto A, Iskander S, Vincent C. Studying large-scale programmes to improve patient safety in whole care systems: Challenges for research. Soc Sci Med. 2009 Dec 1;69(12):1767–76.

60. Harrison MB, Legare F, Graham ID, Fervers B. Adapting clinical practice guidelines to local context and assessing barriers to their use. CMAJ. 2010;182(2):78–84.

61. Hodkinson P, Argent A, Wallis L, Reid S, Perera R, Harrison S, et al. Pathways to care for critically Ill or injured children: A cohort study from first presentation to healthcare services through to admission to intensive care or death. PLoS One. 2016;11(1).

62. Das M, Mathur T, Ravi S, Meneguim AC, Iyer A, Mansoor H, et al. Challenging drug-resistant TB treatment journey for children, adolescents and their care-givers: A qualitative study. PLoS One [Internet]. 2021;16(3 March):1–15. Available from: http://dx.doi.org/10.1371/journal.pone.0248408

63. Seddon JA, Hesseling AC, Godfrey-Faussett P, Schaaf HS. High treatment success in children treated for multidrug-resistant tuberculosis: An observational cohort study. Thorax. 2014;69(5):471–7.

64. The Global Fund. A Toolkit for Health Facilities Differentiated Care For HIV and

For peer review only

For peer review only - http://bmjopen.bmj.com/site/about/guidelines.xhtml

Tuberculosis [Internet]. The Global Fund. 2015 [cited 2021 Oct 20]. Available from: https://reliefweb.int/report/world/toolkit-health-facilities-differentiated-care-hiv-and-tuberculosis

65. World Health Organization. Integrated care models: an overview. Heal Serv Deliv Program [Internet]. 2016;42. Available from: http://www.euro.who.int/pubrequest%0Ahttp://www.euro.who.int/pubrequest%0Ahttp://www.euro.who.int/pubrequest%0Ahttp://www.euro.who.int/en/health-topics/Health-systems/health-services-delivery/publications/2016/integrated-care-models-an-overview-2016

66. Epstein RM, Legacy GES. The Biopsychosocial Model 25 Years Later : 2004;576–82.

67. Babiker A, Husseini M El, Nemri A Al, Frayh A Al, Juryyan N Al, Faki MO, et al. Current Opinion Health care professional development : Working as a team to improve patient care. 2014;14(2):9–16.

68. Leeper SC, Montague BT, Friedman JF, Flanigan TP. Lessons learned from family-centred models of treatment for children living with HIV: Current approaches and future directions. J Int AIDS Soc. 2010;13(SUPPL. 2).

69. Le Roux, Sacha, Jassat, Waasila, Dickson-hall, Lindy, Mitrani, Leila, Cox, Helen, Milsana K, et al. The role of healthcare champions in policy implementation for decentralised drug-resistant tuberculosis care in South Africa. Manuscr under Rev BMJ Glob Heal.

70. The Global Fund. THE IMPACT OF COVID-19 ON HIV , TB AND MALARIA SERVICES AND SYSTEMS FOR HEALTH : A SNAPSHOT FROM 502 HEALTH FACILITIES [Internet]. Geneva; 2021. Available from: https://www.theglobalfund.org/media/10776/covid-19_2020-disruption-

For peer review only

impact_report_en.pdf

71. Chin DP, Hanson CL. Finding the Missing Tuberculosis Patients. J Infect Dis. 2017;216(Suppl 7):S675–8.

72. Vaikuntam BP, Middleton JW, Mcelduff P, Pearse J, Walsh J, Cameron ID, et al. Assessing the impact of care pathways on potentially preventable complications and costs for spinal trauma patients : protocol for a data linkage study using cohort study and administrative data. 2018;10–4.

73. Sharwood LN, Whyatt D, Vaikuntam BP, Cheng CL, Noonan VK, Joseph AP, et al. A geospatial examination of specialist care accessibility and impact on health outcomes for patients with acute traumatic spinal cord injury in New South Wales , Australia : a population record linkage study. 2021;4:1–13.

74. Moscibrodzki P, Parkinson, S, Ferry R, Nwaneri, N, Thies W. Mapping the technology landscape of national TB programs. Geneva; 2021.

75. World Health Organization; Monitoring the building blocks of health systems : A handbook of indiators and their measurement strategies. Geneva; 2010.

76. World Health Organization, Organisation for Economic Co-operation and Development TWB. Delivering quality health services: A global imperative for universal health coverage. Geneva; 2018.

77. Ramsden R, Davies S, Richard MPH, Ba C, Service H, Shannon M, et al. Collaborative care : Primary health workforce and service delivery in Western New South Wales — - A case study. 2021;(July):768–78.

78. Taber JM, Ph D, Leyva B, Persoskie A, Ph D. Why do People Avoid Medical Care ? A Qualitative Study Using National Data. 2008;18–24.

79. Kruk ME, Gage AD, Arsenault C, Jordan K, Leslie HH, Roder-dewan S, et al. The

For peer review only

For peer review only - http://bmjopen.bmj.com/site/about/guidelines.xhtml

Lancet Global Health Commission High-quality health systems in the Sustainable Development Goals era : time for a revolution. 2018;6(November):1196–252.

80. World Health Organization. Action on the Social Determinants of Health: Learning from Previous Experiences. Social Determinants of Health Discussion Paper 1 [Internet]. 2010. Available from: http://www.who.int/social_determinants/corner/SDHDP1.pdf?ua=1

81. Dookie S, Singh S. Primary health services at district level in South Africa: a critique of the primary health care approach. BMC Fam Pract. 2012;13:67.

82. World Health Organization. Global Tuberculosis Report 2015. Geneva; 2015.

For peer review only

Pattern name	Fan	Chicken foot	Hub & spoke	Triangle	Line	Dot
**Level of decentralisation**	Highly centralised care	Care decentralised to district level with high reliance on COE	Care decentralised to district level	Multi-level / multi-facility decentralised care	Community based, ambulatory care	Community based, ambulatory care
**Description of care delivery**	Hospital based care with long admissions, far from home.	Hospital based care with long admissions, some care closer to home.	Hospital based care with long admissions, closer to home.	Flexible care, coordinated by specialist doctors,.	Home based care, with short admissions if required.	Home based, outpatient care.
**Diagnosis**	At clinic or general☐ hospital.	At clinic or general☐ hospital	At clinic or general hospital	At clinic or general☐ hospital	At clinic	At clinic
**Treatment initiation**	At provincial COE	At specialist TB hospital, but with frequent change to regimen at COE.	At specialist TB hospital	At specialist TB hospital or by specialist TB outreach service.	At MDR/RR-TB clinic or district hospital MDR/RR-TB clinic	At the diagnosing clinic
**Co-ordination of care and follow up**	Specialist TB doctors at the COE	Specialist TB doctors at the COE and TB doctors at the decentralised site.	TB doctors at the specialised site	Specialist physician or TB physician on site or through telemedicine.	TB nurses at clinics. Sometimes assisted by a generalist doctor.	TB nurses at clinics. Sometimes assisted in person/telephonically by generalist doctor.
**Hospitalisation**	At COE, frequently for long periods.	At specialist TB hospital or COE, frequently for long periods.	At specialist TB hospital for long periods.	Depending on patient's medical and social circumstances.	Depending on patient's medical and social circumstances.	No admission.
**Number of patients**	N=41 (urban n=25, rural n =16)	N=19 (urban n=5, rural n=14)	N=72 (urban n=35, rural n=37)	N=30 (urban n=11, rural n=19)	N=12 (urban n=4, rural n=8)	N=17 (urban n=8, rural n=9)
**Median distance from home to diagnosing site****	10km (n=36)	11.4km (n=17)	14.6km (n=66)	10.6km (n=24)	5.4km (n=6)	0.9km (n=14)
**Median distance from home to initiating site****	44km (n=36)	38km (n=17)	41km (n=66)	26km (n=24)	6km (n=6)	2km (n=14)

For peer review only - http://bmjopen.bmj.com/site/about/guidelines.xhtml

**Median distance travelled between facilities**	102 km	188km	51km	76km	8km	0km
**Median time to treatment initiation*** **	35 days	13 days	14 days	7 days	0 days	1 day
**Median (range) number of trips********	6 (1-7)	5 (2-12)	6 (1-17)	8 (2-16)	6 (1-21)	8 (6-22)
**Median duration of hospital admission**	79 days (IQR=105) Range 0 - 240	103 days (IQR=91) Range 20 - 197	81 days (IQR=124 Range 0 - 281	40 days (IQR=57) Range 0 - 175	12 days (IQR=24) Range 0 - 38	0 days (IQR=0) Range:0 - 0

* n = number of patients ** distance travelled calculated as straight-line distance as calculated from the patient's home to the health care site. *** Days from MDR/RR-TB diagnosis to treatment initiation **** Number of trips undertaken to health care sites during first 9 months of treatment. ☐General hospital indicates either a district or regional level hospital. COE – TB Centre of Excellence

<u>Table 1: Dominant geospatial movement patterns observed within study districts, together with key features of organisation of care within these patterns and descriptive statistics of patient journeys.</u>

For peer review only

Province	Rural or urban	Districts	Fan	Chicken foot	Hub & Spoke	Triangle	Line	Dot
**KwaZulu -Natal**	Urban	Ethekwini	4	3	5	1		1
		Uthungulu		4	2	7		2
	Rural	Ilembe*	2		8	5		
		Umkanyakude		2	7			6
		Uthukela		6	8			1
**Western Cape**	Urban	City of Cape Town	5	2			3	5
		Eden			12	2		1
	Rural	Cape Winelands			8	2	2	3
		West Coast	1		9		2	2
**Eastern Cape**	Urban	Buffalo City	14					
		Nelson Mandela Bay			10	3	1	1
	Rural	OR Tambo	8	1	1		4	1
		Sarah Baartman	7	2	2	2		1

iLembe* – undergoing rural to urban transition

Numbers in cells refer to number of individual patient pathways observed for each organisational model within the district

Table 2: The distribution of organisational models by study district.

For peer review only

PATIENT FEATURES	Category	Fan *n=41	Chicken foot n=19	Hub & spoke n=72	Triangle n=30	Line n=12	Dot n=17	Total n=191
Age category (yrs)	0-19	3 (7)	1 (5)	3 (4)	2 (7)	0	0	9 (5)
	>60	0	0	4 (6)	1 (3)	0	0	5 (3)
Not receiving income		30 (73)	14 (74)	49 (68)	18 (60)	4 (33)	9 (53)	124 (65)
HIV positive		29 (71)	16 (84)	50 (70)	18 (60)	11 (92)	7 (41)	131 (69)
Baseline CD4 <200 c/ul**	<200	14 (48)	6 (38)	15 (30)	5 (26)	5 (50)	2 (29)	47 (35)
	Unreported	6 (21)	5 (31)	18 (36)	7 (39)	3 (30)	2 (29)	41 (32)
Previous TB history	DS-TB any episode	25 (61)	14 (74)	49 (68)	19 (63)	6 (50)	8 (47)	121 (63)
	MDR/RR-TB 1 episode	3 (7)	4 (21)	6 (8)	3 (10)	3 (25)	1 (6)	20 (10)
	MDR/RR-TB ≥2 episodes	5 (12)	1 (5)	9 (13)	1 (3)	0	0	16 (9)
Baseline BMI <18.5 kg/m2	<18.5	10 (24)	7 (37)	25 (35)	7 (23)	5 (41)	4 (24)	58 (49)
	Unreported	12 (29)	4 (21)	21 (29)	12 (40)	7 (58)	4 (24)	60 (51)
Comorbidities per patient (excluding HIV)	1-3	4 (10)	4 (21)	15 (20)	10 (33)	2 (17)	2 (12)	37 (19)
	≥4	0	0	2 (3)	1 (3)	4 (33)	2 (12)	9 (5)
2nd line TB drug resistance	Pre – XDR	11 (27)	6 (32)	8 (11)	4 (13)	1 (8)	1 (6)	31 (16)
	XDR	6 (15)	1 (5)	8 (11)	2 (7)	1 (8)	1 (6)	19 (10)
	Unknown	2 (5)	1 (5)	10 (14)	2 (7)	1 (8)	3 (18)	19 (10)

*Number of patients **Within 3 months of diagnosis % refer to proportion of patients within each pattern

Table 3: Characteristics of the study patients within each organisational pattern.

For peer review only - http://bmjopen.bmj.com/site/about/guidelines.xhtml

PATTERN NAME	Fan	Chicken foot	Hub & spoke	Triangle	Line	Dot
**Advantages of the organisational model for the patient**	Access to multidisciplinary team with experienced clinicians, drug availability.<br><br>Access to acute tertiary (ICU, dialysis) and chronic tertiary care services (nephrology, cardiology, neurology).	Patients with less complex illness are able to be cared for in their districts.<br><br>Ready referral pathway back to the centralised site for patients with complex illness.	Patients are cared for in their districts, closer to home .<br><br>Specialist TB hospitals may be better designed for long-term care than COEs. Staff are more familiar with individual patients and their conditions and contexts, compared to COE.	Patients able to access care at different facilities, depending on preference.<br><br>As a collective, the facilities have multidisciplinary resources.<br><br>Expert conductor has the ability to navigate care and advocate for holistic care. Oversight for patients with complex conditions.	Care close to home.<br><br>Initial period of supervised inpatient care may provide nutrition support, side effect monitoring, linkage to financial grant support & patient education.	Care very close to home<br><br>Increased focus on community, patient- and family-centred care, with staff embedded within the community<br><br>Other decentralised services, eg, HIV care, family planning, are easily accessible.
**Advantages of the organisational model for the health system**	Logistically simple to compile statistics & monitor patients and train specialised staff.<br><br>Rapid response to change in diagnostic or drug regimens.<br><br>Centralization of expensive drug storage & control – minimise waste, expiry or shrinkage.	Reduces bed pressure on centralised sites, whilst maintaining strong clinical governance of patients with complex illness.<br><br>Less reliance on one facility per province (redundancy).	Strong connections between specialist TB hospital and clinics facilitates patient monitoring and surveillance.<br><br>The district node taking a high degree of responsibility promotes accountability.<br><br>Early treatment modification by TB specialists at the district level reduces admissions.	Oversight from an expert conductor facilitates rapid adjustment of patient care, reducing hospitalization and optimising resource usage.<br><br>Leverage of resources by senior clinicians.<br><br>Flexibility around staffing allows system to buffer more easily.	Outpatient review within the community, with option for short hospital admission for treatment optimisation and stabilisation.<br><br>Reduces patient transport (infection transmission, expense) and promotes family support, whilst minimising the need to provide services and drugs to every clinic.	Rapid diagnosis and treatment initiation.<br><br>Reduced requirement for patient transport.<br><br>Patient trust in local staff promotes adherence and treatment completion.<br><br>Support from decentralised HIV programmes.
**Challenges of the organisational model for the patient**	Treatment delay, long admission times, hospital acquired infection, poor family centredness, loss of patient income.	Patients need to adjust to care in dfferent hospitals.<br><br>Fragmentation of care	District sites may have reduced support from COEs. This may result in difficulty referring patients requiring more complex care.	Care may be fragmented between the sites & patients may become lost to follow-up between sites.<br><br>Requires support from the	Sites may have reduced support from COEs. This may result in difficulty referring patients requiring more complex care.	Sites may have reduced support from COEs. This may result in difficulty referring patients requiring more complex care.

For peer review only - http://bmjopen.bmj.com/site/about/guidelines.xhtml

	Community stigma of being at a CoE for 'strong' TB.<br><br>Disconnect from community chronic care services.	Travel between sites.<br><br>Moderate to long admission times.	Moderate to long admission times.	province (travel, information technology services, training) which may not be consistent.	Multidisciplinary team not always available. Complications may go undetected or there may be a delay in referring for higher levels of care.	Multidisciplinary team less available. Complications may go undetected or delay in referral for higher-level care.
**Challenges of the organisational model for the health system**	Reliance on a single centre - lack of redundancy.<br><br>Skill set 'owned' by a few individuals at the COE's. Other HCWs see themselves as unable to care for MDR/RR-TB reducing willingness to care for these patients.	Over-reliance on COE, with decentralised site poorly empowered to manage MDR/RR-TB patients.<br><br>Providing safe patient transport.	Depending on the geographical size of the province, many patients still travel far distances to visit the TB service or be admitted to hospital.	Reliance on retention of individual specialist physicians to maintain the program.<br><br>Tracking patient progress through the system.<br><br>Providing safe patient transport.	Monitoring & supporting many MDR-TB sites. Providing sites with laboratory & IT infrastructure, drugs, trained staff and supervision.<br><br>Ensuring quality of care provision and timely capturing of accurate data.	Monitoring & supporting many MDR-TB sites. Providing sites with laboratory & IT infrastructure, drugs, trained staff, and supervision.<br><br>Ensuring quality of care provision and timely capturing of accurate data.

Table 4. Advantages and challenges associated with each organisational care pattern

For peer review only

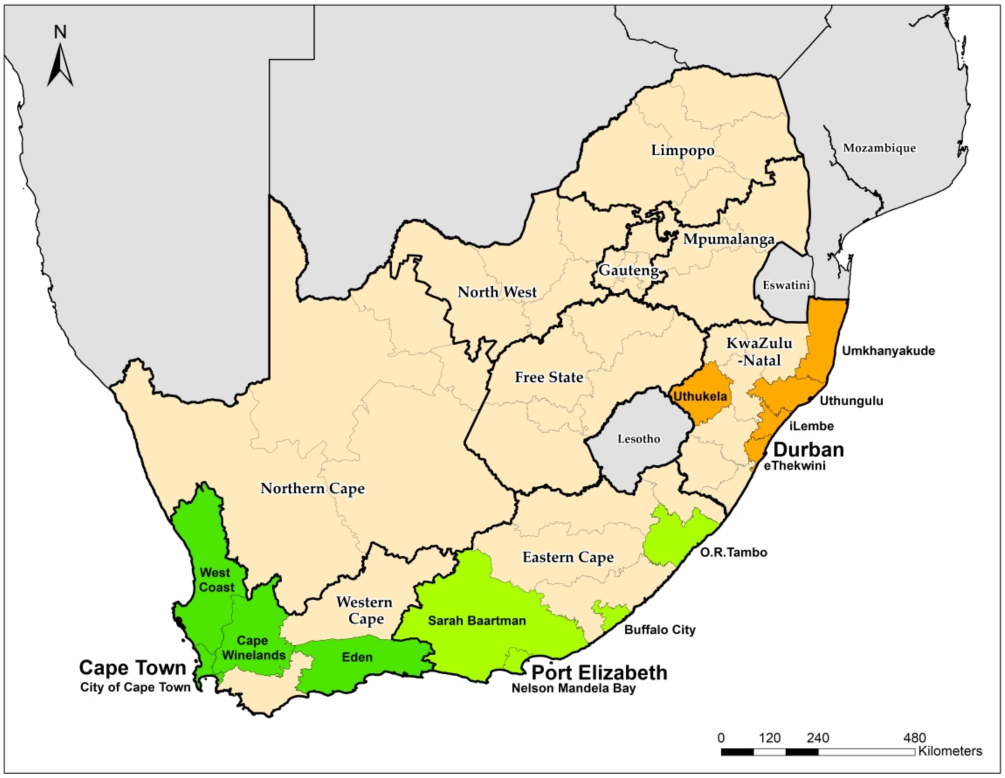

Figure 1: South African map showing thirteen districts selected for study in three provinces. Districts are shaded according to Province (shaded darker green in Western Cape, lighter green in Eastern Cape and orange in KwaZulu-Natal).

1420x1097mm (118 x 118 DPI)

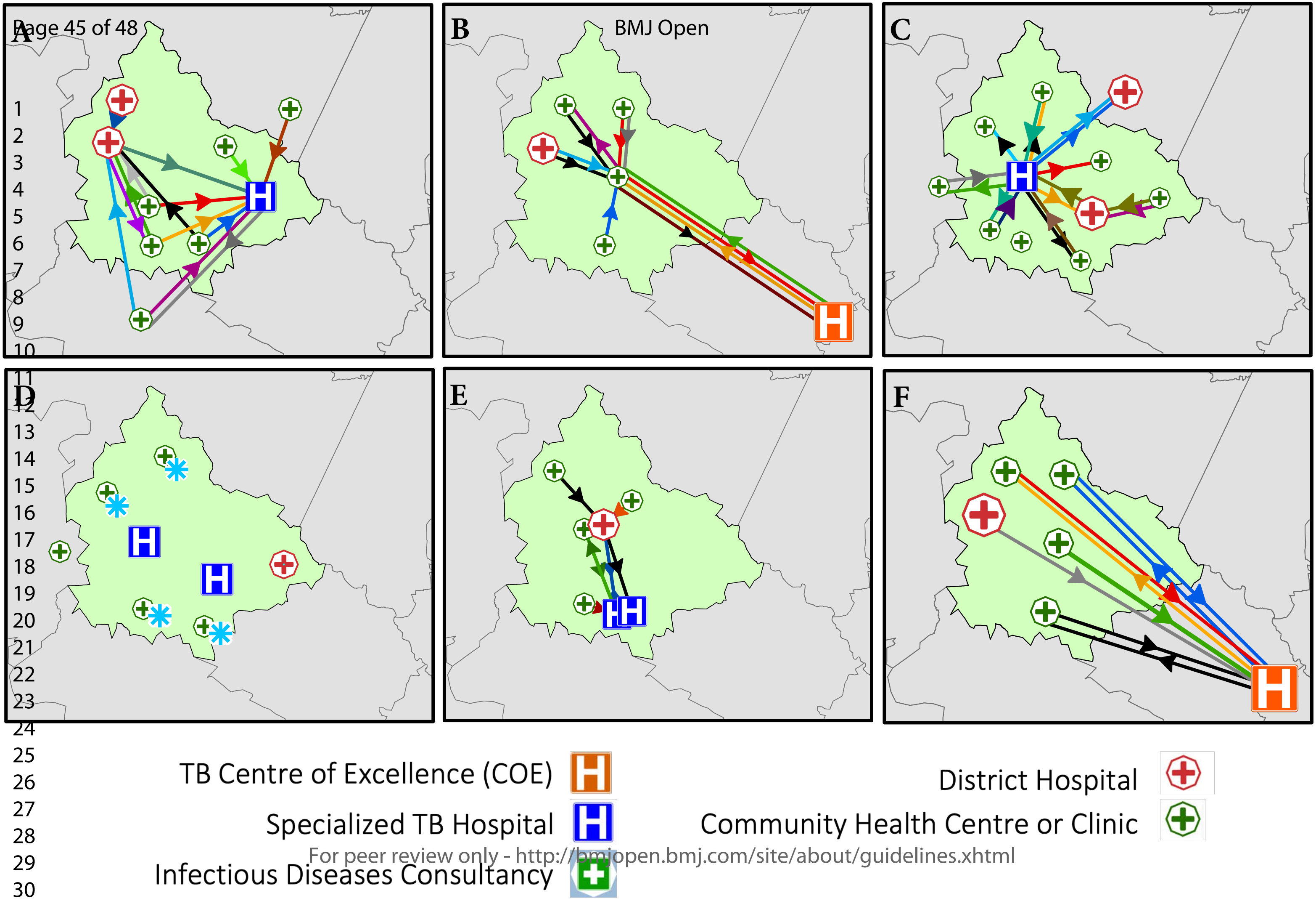

For peer review only - http://bmjopen.bmj.com/site/about/guidelines.xhtml

Supplementary material: A

Additional information related to COREQ (Consolidated criteria for Reporting Qualitative research) Checklist

Domain 1: Research team & reflexivity

LD, JH, SLR and LM conducted interviews. The researchers were a team of clinicians and social science degree majors qualified in the fields of medicine, biology and teaching. At the time of the study all were full time researchers on the project. Three were female and one was male. Three were South African and one was Australian. Three had Masters degrees and one had a Bachelors degree. LD had been employed as a clinician in the DR-TB, HIV and family medicine programs in KZN and the Western Cape and had been previously working on a DR-TB linkage to care study. JH had been employed as a clinician in the DR-TB and infectious diseases domain at Papua New Guinea. SLR had been teaching senior high school students and working in a health care call centre and medical practice previously. LM had been completing a master's degree in biology involving geo-informatics. LD and JH had been investigators in previous DR-TB studies. There was no formal relationship established prior to the study, except some familiarity between the local DR-TB clinicians and LD. The participants were aware of research aims and objectives and the names of the researchers and investigators. The participants were aware that the researchers were interested in health systems around DR-TB disease in SA.

Domain 2: Study design

Questions and guides were piloted with a smaller group of local DR-TB staff at a non-study facility and then provided to participants by the interviewers, together with patient journey maps obtained from that region to serve as probes for some questions. Audio recordings were obtained, except in the case of a correctional services facility where recording was not permitted. A single round of interviews took place at the work place during tea or lunch breaks. Field notes were made during and then more comprehensively after the interview. The duration of the interviews ranged from 30-50 minutes, and depended on the operational work time constraints and the degree of insight and knowledge held and shared by the participant. Data saturation was discussed before ending interviews. Transcripts were not returned for comment or correction.

Domain 3: Analysis and findings

SLR and LD reviewed the interviews and coded the data, using a simple, unstructured coding scheme to extract information on the implications of the patterns of care for patients or the health care system . Participants did not give feedback on the data, except at an end-of-study feedback session with the Departments of Health, which was not used to inform this manuscript. Participant quotations were not presented. There was consistency between the data presented and the findings. Major themes were clearly presented in the findings. The nature of the patient pathway analysis allowed for interrogation of both major themes (common reasons for patient movement) as well as minor themes less common reasons for patient movement).

For peer review only - http://bmjopen.bmj.com/site/about/guidelines.xhtml

For peer review only

Supplementary information: B

		Rural or urban	Demographic and health care descriptors					Poverty indices				Death and disease profile						
			Population count	Population density / km²	Number of clinics	Number of public hospitals	Provincial spend on health / person / year (ZAR)*	Population below lower poverty line	Unemployed	Informal shack dwellings	Illiteracy	Life expectancy (yrs)	% of deaths due to infectious diseases	Leading cause of natural death	HIV / TB coinfection	TB incidence / 100 000 in 2015	Number of MDR/RR-TB cases in 2017** XDR	MDR
PROVINCE	KWAZULU-NATAL						R3 527	44%	28%		15%	57-64			69%	685	115	1687
DISTRICT	eThekwini (37)***	City (metro)	3 702 231	1502	31	18		41%	19%	13%	17%		16%	Heart disease		698	85	1063
	uThungulu (38)	City (small)	971 135	88	83	9		72%	34%	3%	12%		21%	TB		859	7	242
	iLembe (39)	Rural (small metro)	657 612	185	36	8		55%	31%	9%	15%		24%	TB		801	10	151
	uMkhanyakude (40)	Rural district	689 090	61	86	5		80%	31%	15%	25%		27%	HIV/ AIDS		577	6	146
	uThukela(41)	Rural district	706 588	63	49	4		63%	29%	1%	10%		24%	TB		533	7	85
PROVINCE	WESTERN CAPE						R3 867	32%	20%		7%	66-71			47%	681	86	1140
DISTRICT	City of Cape Town (42)	City (metro)	4 208 000	1530	130	18		31%	22%	11%	2%		14%	Diabetes		596	59	757
	Eden (43)	City (metro)	611 279	25	209	7		33%	27%	8%	12%		17%	Diabetes		809	5	100
	Cape Winelands (44)	Rural district	917 462	40	134	10		34%	17%	7%			18%	Diabetes		880	14	170
	West Coast (45)	Rural district	436 403	15	180	7		30%	19%	8%	13%		16%	Heart disease		837	8	113
PROVINCE	EASTERN CAPE						R3 046	56%	47%		18%	60-67			56%	692	281	1151
DISTRICT	Buffalo City (43)	City (metro)	893 157	300	103	10		41%	35%	25%	12%		21%	TB		743	77	314
	Nelson Mandela Bay(44)	City (metro)	1 263 051	615	68	12		51%	63%	7%	1%		20%	Diabetes		938	150	471
	OR Tambo (41)	Rural district	1 457 384	108	147	16		67%	39%	1%	14%		20%	TB		571	22	220
	Sarah Baartman (46)	Rural district	520 480	8	89	15		41%	30%	9%	13%		20%	TB		1022	32	146

Table S1. Description of key health care and demographic features of the study districts

* ZAR = South African Rands (1 US dollar is approximately 17 ZAR)

** Data from National Institute of Communicable Diseases

***Reference numbers for data sources are given following each district name

For peer review only - http://bmjopen.bmj.com/site/about/guidelines.xhtml

COREQ (COnsolidated criteria for REporting Qualitative research) Checklist

A checklist of items that should be included in reports of qualitative research. You must report the page number in your manuscript where you consider each of the items listed in this checklist. If you have not included this information, either revise your manuscript accordingly before submitting or note N/A.

Topic	Item No.	Guide Questions/Description	Reported on Page No.
**Domain 1: Research team and reflexivity**			
Personal characteristics			
Interviewer/facilitator	1	Which author/s conducted the interview or focus group?	
Credentials	2	What were the researcher's credentials? E.g. PhD, MD	
Occupation	3	What was their occupation at the time of the study?	
Gender	4	Was the researcher male or female?	
Experience and training	5	What experience or training did the researcher have?	
Relationship with participants			
Relationship established	6	Was a relationship established prior to study commencement?	
Participant knowledge of the interviewer	7	What did the participants know about the researcher? e.g. personal goals, reasons for doing the research	
Interviewer characteristics	8	What characteristics were reported about the inter viewer/facilitator? e.g. Bias, assumptions, reasons and interests in the research topic	
**Domain 2: Study design**			
Theoretical framework			
Methodological orientation and Theory	9	What methodological orientation was stated to underpin the study? e.g. grounded theory, discourse analysis, ethnography, phenomenology, content analysis	
Participant selection			
Sampling	10	How were participants selected? e.g. purposive, convenience, consecutive, snowball	
Method of approach	11	How were participants approached? e.g. face-to-face, telephone, mail, email	
Sample size	12	How many participants were in the study?	
Non-participation	13	How many people refused to participate or dropped out? Reasons?	
Setting			
Setting of data collection	14	Where was the data collected? e.g. home, clinic, workplace	
Presence of non-participants	15	Was anyone else present besides the participants and researchers?	
Description of sample	16	What are the important characteristics of the sample? e.g. demographic data, date	
Data collection			
Interview guide	17	Were questions, prompts, guides provided by the authors? Was it pilot tested?	
Repeat interviews	18	Were repeat inter views carried out? If yes, how many?	
Audio/visual recording	19	Did the research use audio or visual recording to collect the data?	
Field notes	20	Were field notes made during and/or after the inter view or focus group?	
Duration	21	What was the duration of the inter views or focus group?	
Data saturation	22	Was data saturation discussed?	
Transcripts returned	23	Were transcripts returned to participants for comment and/or	

For peer review only

Topic	Item No.	Guide Questions/Description	Reported on Page No.
		correction?	
**Domain 3: analysis and findings**			
Data analysis			
Number of data coders	24	How many data coders coded the data?	
Description of the coding tree	25	Did authors provide a description of the coding tree?	
Derivation of themes	26	Were themes identified in advance or derived from the data?	
Software	27	What software, if applicable, was used to manage the data?	
Participant checking	28	Did participants provide feedback on the findings?	
Reporting			
Quotations presented	29	Were participant quotations presented to illustrate the themes/findings? Was each quotation identified? e.g. participant number	
Data and findings consistent	30	Was there consistency between the data presented and the findings?	
Clarity of major themes	31	Were major themes clearly presented in the findings?	
Clarity of minor themes	32	Is there a description of diverse cases or discussion of minor themes?	

Developed from: Tong A, Sainsbury P, Craig J. Consolidated criteria for reporting qualitative research (COREQ): a 32-item checklist for interviews and focus groups. *International Journal for Quality in Health Care*. 2007. Volume 19, Number 6: pp. 349 – 357

Once you have completed this checklist, please save a copy and upload it as part of your submission. DO NOT include this checklist as part of the main manuscript document. It must be uploaded as a separate file.

For peer review only

BMJ Open

The organisation of care for people receiving drug-resistant tuberculosis treatment in South Africa: a mixed methods study

Journal:	*BMJ Open*
Manuscript ID	bmjopen-2022-067121.R1
Article Type:	Original research
Date Submitted by the Author:	30-Aug-2023
Complete List of Authors:	Dickson, Lindy; University of Cape Town Faculty of Health Sciences, Department of Pathology<br>Le Roux, Sacha; University of Cape Town Faculty of Health Sciences, Department of Pathology<br>Mitrani, Leila; University of Cape Town<br>Hill, Jeremy; London School of Hygiene &Tropical Medicine , TB Centre<br>Jassat, Waasila; University of the Western Cape School of Public Health<br>Cox, Helen; University of Cape town, Medical Microbiology<br>Mlisana, Koleka; University of KwaZulu-Natal College of Health Sciences, School of Laboratory Medicine and Medical Sciences<br>Black, John; University of Cape Town Faculty of Health Sciences, Division of Infectious Diseases<br>Loveday, Marian; Medical Research Council of South Africa<br>Grant, Alison; London School of Hygiene & Tropical Medicine, TB Centre; Africa Health Research Institute<br>Kielmann, Karina; Institute of Tropical Medicine<br>Ndjeka, Norbert; National Department of Health; University of KwaZulu-Natal<br>Moshabela, Mosa; University of KwaZulu-Natal College of Health Sciences, School of Nursing and Public Health<br>Nicol, Mark; University of Cape Town; University of Western Australia
**Primary Subject Heading**:	Health services research
Secondary Subject Heading:	Infectious diseases, Patient-centred medicine, Health policy, Public health, Qualitative research
Keywords:	Tuberculosis < INFECTIOUS DISEASES, Health policy < HEALTH SERVICES ADMINISTRATION & MANAGEMENT, PUBLIC HEALTH, QUALITATIVE RESEARCH, Patient-Centered Care, Organisation of health services < HEALTH SERVICES ADMINISTRATION & MANAGEMENT

SCHOLARONE™
Manuscripts

I, the Submitting Author has the right to grant and does grant on behalf of all authors of the Work (as defined in the below author licence), an exclusive licence and/or a non-exclusive licence for contributions from authors who are: i) UK Crown employees; ii) where BMJ has agreed a CC-BY licence shall apply, and/or iii) in accordance with the terms applicable for US Federal Government officers or employees acting as part of their official duties; on a worldwide, perpetual, irrevocable, royalty-free basis to BMJ Publishing Group Ltd ("BMJ") its licensees and where the relevant Journal is co-owned by BMJ to the co-owners of the Journal, to publish the Work in this journal and any other BMJ products and to exploit all rights, as set out in our licence.

The Submitting Author accepts and understands that any supply made under these terms is made by BMJ to the Submitting Author unless you are acting as an employee on behalf of your employer or a postgraduate student of an affiliated institution which is paying any applicable article publishing charge ("APC") for Open Access articles. Where the Submitting Author wishes to make the Work available on an Open Access basis (and intends to pay the relevant APC), the terms of reuse of such Open Access shall be governed by a Creative Commons licence – details of these licences and which Creative Commons licence will apply to this Work are set out in our licence referred to above.

Other than as permitted in any relevant BMJ Author's Self Archiving Policies, I confirm this Work has not been accepted for publication elsewhere, is not being considered for publication elsewhere and does not duplicate material already published. I confirm all authors consent to publication of this Work and authorise the granting of this licence.

For peer review only

Title: The organisation of care for people receiving drug-resistant tuberculosis treatment in South Africa: a mixed methods study

Corresponding author: Mark Nicol

Email: mark.nicol@uwa.edu.au

Tel: (+61) 410 584 165

Address: School of Biomedical Sciences

University of Western Australia

M504, Perth, WA 6009, Australia

Author names and affiliations:

Lindy Dickson[1], Sacha Le Roux[1], Leila Mitrani[1], Jeremy Hill[3,4], Waasila Jassat[2], Helen Cox[1,5], Koleka Milsana[6], John Black[7,8], Marian Loveday[9,10], Alison D. Grant[11,12,], Karina Kielmann[13,14,] , Norbert Ndjeka[15,16,] , Mosa Moshabela [12,18], Mark P. Nicol[1,17]

1. Division of Medical Microbiology, University of Cape Town, Cape Town, South Africa
2. School of Public Health, University of the Western Cape, Cape Town, South Africa
3. Faculty of Medicine and Health, The University of Sydney, Sydney, New South Wales, Australia
4. Tuberculosis Research Program, Centenary Institute Medical Research Foundation, Newtown, New South Wales Australia
5. Institute for Infectious Disease and Molecular Medicine and Wellcome Centre for Infectious Disease Research in Africa, University of Cape Town, Cape Town, South Africa

For peer review only - http://bmjopen.bmj.com/site/about/guidelines.xhtml

6. School of Laboratory Medicine and Medical Sciences, University of KwaZulu-Natal, Durban, South Africa
7. Department of Infectious Diseases, Livingstone Hospital, Port Elizabeth, South Africa
8. Division of Infectious Diseases, Department of Medicine, University of Cape Town, Cape Town, South Africa
9. HIV and other Infectious Diseases Research Unit, South African Medical Research Council, South Africa
10. Centre for the AIDS Programme of Research in South Africa (CAPRISA), University of KwaZulu-Natal, Durban, South Africa
11. TB Centre, London School of Hygiene & Tropical Medicine, London, UK
12. Africa Health Research Institute, School of Laboratory Medicine and Medical Sciences, College of Health Sciences, University of KwaZulu-Natal, Durban, South Africa
13. Institute of International Health and Development, Queen Margaret University, Edinburgh, United Kingdom
14. Equity and Health Unit, Department of Public Health, Institute of Tropical Medicine Antwerp, Antwerp, Belgium
15. National Department of Health, Tuberculosis Control and Management Cluster, Pretoria, South Africa
16. Nelson R Mandela School of Medicine, University of KwaZulu-Natal, Durban, South Africa
17. Marshall Centre, Division of Infection and Immunity, School of Biomedical Sciences, University of Western Australia, Perth, Australia
18. School of Nursing and Public Health, University of KwaZulu-Natal, Durban, South Africa

For peer review only

Key Words: tuberculosis, drug-resistant, decentralisation, patient pathways, models of care, health care systems, person-centred care, South Africa

For peer review only

For peer review only - http://bmjopen.bmj.com/site/about/guidelines.xhtml

ABSTRACT

Objectives

Treatment for multidrug-resistant/rifampicin-resistant tuberculosis (MDR/RR-TB) is increasingly transitioning from hospital-centred to community-based care. A national policy for decentralised programmatic MDR/RR-TB care was adopted in South Africa in 2011. We aimed to explore variations in the implementation of care models in response to this change in policy, and the implications of these health service variations for people affected by MDR/RR-TB.

Methods

A multiple case study methodology was used to study 13 high-burden districts within three provinces. Movements of 191 people with MDR/RR-TB between health care facilities were reconstructed from laboratory records, verified by patient folder reviews. The resultant geospatial patterns were used to identify organisational models. Facility visits and staff interviews were used to confirm the models, identify reasons for patient movement, and determine implications of different organisational models for people affected by MDR/RR-TB and the health system.

Results

Six dominant geospatial movement patterns were identified, which varied in average hospital stay (0-281 days), average patient distance travelled (12-198km) and number of health facilities involved in care (1–5 facilities). More centralised models, which relied on ‘Centres of Excellence’ for care provision and support, were associated with longer delays to treatment initiation and lengthy hospitalisation. Decentralised models facilitated family-centered care and were associated with reduced time to treatment and hospitalisation. Flexibility to respond

For peer review only

For peer review only - http://bmjopen.bmj.com/site/about/guidelines.xhtml

to the needs of people affected by MDR/RR-TB and health system constraints was achieved through implementation of flexible models, or the implementation of multiple models in a district.

Conclusions

Understanding how models for organising care have evolved may assist policy implementers to tailor implementation to promote particular patterns of care organisation or encourage flexibility, based on patient needs and local health system resources. Our approach can contribute towards the development of a health systems typology for understanding how policy-driven models of service delivery are implemented in the context of variable resources.

Strengths and limitations of this study

- We used real-world patient movement pathways to understand emerging models of care in response to a change in policy.
- We triangulated several different sources of data, including laboratory data, interviews with health care providers and facility visits to understand the implications of these emerging models of care for people affected by MDR/RR-TB and the health system.
- We could not clearly differentiate to what degree patient needs versus health system organisation and resources determined individual care pathways.
- We were not able to adequately assess care quality indicators, and so are unable to determine whether particular organisational models were associated with favourable patient outcomes.

For peer review only - http://bmjopen.bmj.com/site/about/guidelines.xhtml

- We did not interview people affected by MDR/RR-TB, and so cannot draw conclusions about their preferences.

For peer review only

For peer review only - http://bmjopen.bmj.com/site/about/guidelines.xhtml

BACKGROUND

Tuberculosis (TB) has been the leading cause of death in South Africa for two decades (1) and claims the lives of around 330 persons daily worldwide(2). Multidrug-resistant (MDR-) TB is defined as tuberculosis caused by *Mycobacterium tuberculosis* that is resistant to at least the two key drugs, rifampicin and isoniazid, whilst rifampicin-resistant (RR-) TB is defined by resistance to rifampicin with or without isoniazid resistance. MDR/RR-TB caused illness in around 14 000 persons in South Africa in 2019(3), is more complex and costly to treat than drug sensitive TB(4), and is associated with a fourfold higher mortality (1,5). Within the next 20 years, the MDR/RR-TB case load in South Africa is projected to increase substantially(6,7). Persons with MDR/RR-TB often experience complex medical, psychological(8), social(9,10), and economic(11) challenges. Management for people with MDR/RR-TB requires an intensive multidisciplinary, comprehensive approach, and typically consumes a disproportionate share of health resources(11). Treatment provision requires access to specialised drugs and skilled staff to monitor people with MDR/RR-TB over many months(11). The sustainable use of newer regimens to address this case load will require strengthening MDR/RR-TB treatment delivery systems to ensure effective, person-centred care(12). The WHO End TB Strategy advises a country-specific, tailored, and strategic approach to achieving universal access to high-quality treatment for MDR/RR-TB(13,14).

In response to the rising MDR/RR-TB caseload in South Africa and to local and international pressure from patient and advocacy groups, the National Department of Health (NDoH) adopted an approach to increase ambulatory management of MDR/RR-TB in 2011 (15). This strategy, which had not been widely implemented elsewhere, but was in line with pilot projects

For peer review only

For peer review only - http://bmjopen.bmj.com/site/about/guidelines.xhtml

in South Africa, Peru and Lesotho, was intended to improve treatment access and reduce in-hospital stays(16,17). Prior to decentralisation, people with MDR/RR-TB in South Africa were primarily managed within facilities specialising in MDR/RR-TB care, frequently with long hospital stays, before being discharged under the ongoing care of the specialised facilities. The decentralisation strategy aimed to increase the number of treatment sites from one or two dedicated MDR/RR-TB facilities per province to several facilities capable of providing MDR/RR-TB care within each district(18). A national guideline for decentralisation of care was published in 2011(19) and updated in 2019(20), and served as a generic, non-prescriptive tool for health managers. Since then, implementation of the policy to decentralise MDR/RR-TB care within South Africa has varied widely (21), in response to differences in health system capacity and readiness to cope with change(22)(23)(22). Funding to support decentralisation was not specifically allocated, which resulted in further setting-specific innovations and adaptations to the program at both provincial and district level(24,25).

We have previously described variation in the care journeys of people with MDR/RR-TB(21), which may reflect differences in patient co-morbidity or disease features, but likely also reflects regional differences in the organisation of care for the management of MDR/RR-TB. Ideally, models for organisation of care should attempt to improve patient outcomes through coordinated care that is person-centred, timely and effective (26); however, models of care often evolve to suit health care providers and health systems convenience (27–29).

We therefore studied variation in the patterns of organisation of care for people with MDR/RR-TB which have emerged in South Africa because of local variation in guideline implementation.

For peer review only - http://bmjopen.bmj.com/site/about/guidelines.xhtml

We used patient care pathways to identify patterns of care provision, outline the implications of these patterns for the needs of people with MDR/RR-TB and draw lessons on different ways in which care for patients with complex illness can be organised.

METHODS

The overarching research project, within which this sub-study was nested, was led by the same group of investigators, funded by a Health Systems Research Initiative award from the Medical Research Council of the United Kingdom and aimed to identify interventions to optimise decentralisation of services for people with MDR/RR-TB (Supplementary Figure 1). Ethical approval for the study was provided by the University of Cape Town, South Africa (350/2016), and the London School of Hygiene & Tropical Medicine, UK (11680). The project used a stepwise realist approach to understand the policy context, implementation and working models of decentralisation of MDR/RR-TB care in South Africa. The aim of this sub-study, which was conducted over the same period, was to explore variations in the implementation of care models in response to a change in MDR/RR-TB health policy, and the implications of these variations for people with MDR/RR-TB and the health services.

Study design and setting

We used a multiple case study methodology (30). Data collection included a retrospective cohort study of geographically-linked laboratory records, validated by patient folder reviews. All TB samples in the state sector in South Africa are sent to laboratories which form part of the National Health Laboratory Services (NHLS) for testing. The NHLS has a centralised data

For peer review only - http://bmjopen.bmj.com/site/about/guidelines.xhtml

repository which records all samples sent to laboratories within the network, together with results and patient details. Patterns of patient movement and facility visits were mapped. Semi-structured interviews with health care workers were undertaken to verify patient journey patterns and organisational patterns, and to determine their implications for the health service and people with MDR/RR-TB. We defined MDR/RR-TB as resistance to rifampicin (with or without isoniazid resistance), detected using genotypic or phenotypic methods, irrespective of resistance to other anti-TB drugs.

Three provinces in South Africa (KwaZulu-Natal [KZN], Eastern Cape [EC] and Western Cape [WC]) were selected due to their high MDR/RR-TB disease burden and differing approaches to decentralised care (Supplementary Figure 2). Within each province, two urban and two rural districts were selected based on observed differences in patient pathways from the parent study(31). One additional rural district was selected in KZN, due to a rapid shift in urbanisation associated with relocation of the province's airport.

The study took place between July 2016 and July 2019. During this period, MDR/RR-TB treatment regimens were largely based on a standardised aminoglycoside-containing regimen (requiring an injectable drug), tailored according to individualised drug-susceptibility patterns. Prior to the study period, rapid molecular testing for TB and MDR/RR-TB (Xpert MTB/RIF) had been implemented, while limited roll-out of the novel anti-tuberculous drug bedaquiline in pilot programmes and the introduction of a shortened MDR/RR-TB treatment regimen occurred over the study period. People with MDR/RR-TB generally underwent scheduled monthly

For peer review only

clinical evaluation and investigation over the treatment duration, in line with World Health Organisation (WHO) guidelines(31).

Selection of people with MDR/RR-TB and assessment of patient movement pathways

We have previously described the methods used to construct MDR/RR-TB patient healthcare journeys(31). Briefly, National Health Laboratory Service laboratory records were used to generate a list of all people in the study districts with new bacteriologically-confirmed MDR/RR-TB from July to September 2016 (n=2 649). A random sample of 195 people with MDR/RR-TB was selected from this list, stratified by district. One patient was found to have been incorrectly diagnosed with MDR/RR-TB, leaving 194 in the analysis. The laboratory records for the 194 people served as tracers of their pathways over a 9-month period(31). Geographic coordinates of healthcare facilities were collected, and spatial-temporal mapping of patient movement was used to reconstruct patient movement pathways in chronological order for each sampled person with MDR/RR-TB, including movement outside the study districts.

Data on treatment progress were collected from clinical record reviews, telephonic enquiries with clinics and the electronic MDR/RR-TB registry [EDRWeb] up to two years after diagnosis. Data on patient movement obtained from laboratory records were confirmed by telephonic enquiries at all health care facilities attended by people with MDR/RR-TB. This input was used to verify laboratory information, determine additional clinic visits (where no laboratory sample was taken), obtain demographic and health data and to identify reasons for movement of sampled people with MDR/RR-TB between facilities. Descriptive statistics for the sampled

For peer review only - http://bmjopen.bmj.com/site/about/guidelines.xhtml

districts and participants were extracted. Time from laboratory diagnosis to treatment initiation and time on treatment were calculated (32).

All movement between health care facilities for each study participant was geospatially mapped, based on ‘as the crow flies’ measurements(33). Patient pathway patterns sharing similar appearance were then grouped according to their pattern and named, as described in other studies(34)(35) (36). Pattern descriptions included the level of facilities visited (ranging from quaternary care level to mobile clinic), movements between facilities, facility admission period or ‘period under care’ (where facilities retained responsibility for monthly follow up of people with MDR/RR-TB after discharge) and sector of care (state or private). The number of visits (or patient-days) spent under care of a centralised service were used to position the patterns on a decentralisation continuum.

Detailed assessment of organisation of services for MDR/RR-TB

A total of 22 facilities representing the spectrum of patterns were chosen for more detailed investigation of organisation of MDR/RR-TB care. These facilities represented six urban and seven rural districts as well as the different levels of care ranging from primary health care to tertiary care. At each facility we conducted patient folder reviews and semi-structured interviews with health workers, including pharmacists (n=2), out-patient physicians (n=2), inpatient physicians (n=6), TB facility managers (n=5), ward nurses (n=9), out-patient nurses (n=8) [including HIV care nurses (n=2), paediatric care nurses (n=2)], and lay health care workers (n=2). Health care workers were selected for interview based on their involvement with the MDR/RR-TB care programme at their facility. No participants refused to participate or dropped out and only the researchers and participant/s were present during interviews. For

For peer review only - http://bmjopen.bmj.com/site/about/guidelines.xhtml

interviews, specific patient trajectories related to each facility were used as prompts for discussion of the reasons for and implications of patient movements between facilities. Additional data on the same themes were extracted from patient folder reviews. The interviews were conducted in English, with some translation from Afrikaans, and recorded, except in a prison where recording was not permitted. Most interviews were conducted with individuals or pairs of informants, with two interviews involving multidisciplinary panels at a facility. Transcripts of interviews and folder review data were reviewed by seven researchers who extracted information relating to the implications of the different patterns for care organisation for both people with MDR/RR-TB and the health services. A COREQ checklist is included (Supplementary Material). To further identify the implications of the different organisational patterns for care outcomes and person-centredness for people with MDR/RR-TB and the health services, we used key metrics including time to treatment, length of hospital stay, number of facilities visited, and distance travelled.

Patient and Public Involvement Statement

This study involved neither patients nor members of the public. Findings from this study were disseminated during national MDR/RR-TB meetings organised by the Department of Health and non-governmental organisation partners and presented to programme implementers from all nine provinces of the country.

RESULTS

Cohort description

For peer review only

For peer review only - http://bmjopen.bmj.com/site/about/guidelines.xhtml

We studied urban and rural districts from three high TB burden provinces (Table S1). A total of 191 participants had sufficient data available to map treatment pathways and outcomes (the remaining 3 participants had too few data points to assess)(37). Of the 191 people with MDR/RR-TB, 63% (n=120) were male and 92% (n=177) were aged 20-59 years. Approximately half of the participants lived in an urban area (46%, n=88). Sixty-three percent (n=121) had previously been treated for TB, with a further 19% (n=36) previously treated for MDR/RR-TB. 69% (n=131) were coinfected with HIV at the time of MDR/RR-TB diagnosis (37). Two thirds (65%, n=124) were unemployed at the time of diagnosis. Fifteen percent (n=28) had a history of correctional services detention. Approximately one quarter (24%, n=46) reported comorbidities other than TB and HIV at diagnosis. Five percent (n=10) had a household member die from TB disease. All participants were treated exclusively in the state health sector, except for two participants who also received treatment in the private sector. We have previously described in detail the clinical comorbidities and social needs of the people with MDR/RR-TB in this cohort(37).

Sixty-four percent (n=122) of the cohort were diagnosed with MDR/RR-TB; 16% (n=31) with pre-extensively drug-resistant (pre-XDR), 10% (n=19) with XDR-TB and 10% (n=19) with MDR/RR-resistance with unknown second-line drug resistance. Of the 191 participants 80% (n=153) were alive at 6 months, and 77% (n=148) were alive at 2 years after laboratory diagnosis. At 24 months after laboratory diagnosis, 46% of participants (n=88/191) had either completed treatment or achieved 'cure', 19% (n=37) had ongoing treatment, 10% (n=19) were lost to follow-up, 2% (n=3) had unknown outcome and 0.5% (n=1) had ongoing illness where TB treatment was deemed futile, and the patient had been referred for palliative care.

For peer review only - http://bmjopen.bmj.com/site/about/guidelines.xhtml

Geospatial patterns of patient movement and their distribution

A total of 191 patient movement pathways could be classified into six dominant geospatial movement patterns (Table 1 and Figure 1). These were named according to the trajectories of patient movements as A 'fan' [n=41 (22%)], B 'chicken-foot' [n=19 (10%)], C 'hub and spoke' [n=72 (38%)], D 'triangle' [n=30 (15%)], E 'line' [n=12 (6%)], and F 'dot' [n=17 (9%)]. The patterns were placed on a continuum from the most centralised, 'fan', to the most decentralised, 'dot'. The 'hub and spoke' configuration was the most frequently observed pattern across all three provinces in both urban [n=35 (18%)] and rural [37 (19%)] settings (Table 2). Several typical patient pathways for each pattern are depicted on a stylised study district in Figure 1.

Description of organisation of care within each of the patterns

We used facility visits, staff interviews and patient record review to describe the organisation of care within each of these patterns. The most centralised pattern, 'fan', is highly dependent on the designated MDR-TB Centre of Excellence (CoE) to manage people with MDR/RR-TB, following initial assessment at a peripheral facility. CoEs were historically designated by the provincial health departments to manage people with complex TB. The first step in increasing decentralisation of MDR/RR-TB services was the establishment of specialised MDR-TB hospitals, as a 'step down' from the CoEs. The 'chicken-foot' pattern demonstrates some decentralisation to these specialised hospitals for routine cases, but with dependence on the CoEs for caring for people with more extensive TB drug resistance or complex presentations. This pattern was common for regions with higher XDR-TB or MDR/RR-TB numbers and where new drugs were being introduced into TB regimens. Although the participant numbers in the

For peer review only

For peer review only - http://bmjopen.bmj.com/site/about/guidelines.xhtml

study limited outcome analysis, observation suggests that this model has the greatest risk for people with MDR/RR-TB for retention in care, treatment outcome and prolonged hospital stay (Table 1). The' hub and spoke' pattern, which was predominately observed in rural districts, primarily utilises specialised MDR-TB hospitals for all care provision, with little capacity existing at primary care level. These three models are considered the more centralised of the six models identified.

The 'triangle' pattern incorporates a more flexible network of MDR-TB services and indicates availability of MDR-TB services at many facilities within the district. Typically, this pattern has oversight by a specialist infectious diseases or TB physician, who is either within the district or closely monitors patient progress through telemedicine. This 'conductor' is responsible for weighing complex social and medical needs and for directing patient movement to an appropriate level of care, according to the clinical need and social circumstances of the patient, at various points during their care. This organisational model is also able to respond to changes in the local availability of MDR-TB resources, by modifying patient referral pathways. This model is frequently associated with decision-making shared between people with MDR/RR-TB and clinicians.

The 'line' and 'dot' patterns reflect a substantial shift to outpatient clinic-based care, frequently including MDR-TB clinical practitioners providing mobile outreach services to different subdistrict primary health care sites. In the line pattern, not all clinics had capacity to initiate treatment, which resulted in some clinics referring to neighbouring clinics, or a district hospital for outpatient treatment initiation or a short admission for early management and stabilisation. In the 'dot' pattern, clinics had capacity to initiate and maintain MDR/RR-TB treatment

For peer review only

independently, with pharmacy and multidisciplinary resources available. This 'dot' model was associated with the best treatment outcomes, however the sample size was small and possible health care worker selection of participants with less severe illness (to cope with a fully decentralised care option) limits generalisability of these findings.

Different patient movement pathways may reflect organisational models within the health system, the incidence of MDR/RR-TB within districts as well as patient features which require delivery of care at a particular level(38). We observed that the distribution of the different movement patterns across districts differed. Most districts had three or four patterns identified (Table 2). This suggests flexibility in the organisation of care within these districts to respond to differing patient or health system needs.

We conducted exploratory analysis to assess whether patient clinical features were associated with certain patterns of care (Table 3). Children, participants older than 60 years and people with XDR -TB/ pre-XDR-TB seldom followed the more decentralised 'line' and 'dot' patterns. There was otherwise no evidence that patterns of care differed by patient HIV status, previous TB treatment, low body mass index, or higher number of co-morbidities; however, the small sample size precludes robust statistical analysis.

Implications of different patterns of organising care for people with MDR/RR-TB and the health system

We observed care pathways, reviewed patient records, visited healthcare facilities, and interviewed facility staff to identify the implications of different organisational patterns on

For peer review only - http://bmjopen.bmj.com/site/about/guidelines.xhtml

people with MDR/RR-TB and the health system (Table 4). More decentralised models ('line' and 'dot') had advantages such as quick linkage to care, shorter hospital stays, and less travel distance for diagnosis and treatment. However, these models of care were compromised at times through the risk of clinical practitioner shortages and less rigorous treatment monitoring. More centralised models ('fan' and 'chicken foot') had advantages such as multidisciplinary clinical, pharmacy, radiological, and laboratory resources in one facility, but had disadvantages including long travel distances, treatment initiation delays, and patient travel distance. (Table 1).

DISCUSSION

This study used a novel approach, combining quantitative and qualitative methods and patient pathway analysis, to describe emerging patterns of decentralised care for MDR/RR-TB in South Africa. We identified six patterns of care organisation, ranging from highly centralised to highly decentralised models, with several different models operational in many study districts. The different models demonstrated distinct advantages and disadvantages for both people with MDR/RR-TB and the health system. Although exploratory, the patterns identified can contribute to developing a health systems typology for understanding how policy-driven models of service delivery are implemented in the context of variable resources and differing patient needs. Explicit recognition of the divergence of organisational models and cross-district learning around different models for organising care may provide opportunity for health system managers to optimise delivery of care in different contexts, and should be considered by policy developers when planning interventions to decentralise care for complex illness.

For peer review only - http://bmjopen.bmj.com/site/about/guidelines.xhtml

Stokes et al have previously described a framework for analysing models of care for patients with multimorbidity(32). A key component of this framework is the organisation of care across different levels of the health system. We and others have previously described other elements of this framework in relation to MDR/RR-TB in South Africa, including the theoretical basis for the decentralisation policy(39), the clinical and social needs of the target population(37,40), and the local adaptations of the decentralisation policy(22,41,42). Our approach to identifying organisational models, which involved analysis of patient movement pathways, may be a useful tool and contribute valuable insights into understanding variation in policy implementation. This approach also provides important metrics, including the number of different facilities visited, and travel distances. These metrics differed substantially between models in our study, with more centralised models associated with prolonged hospitalisation and multiple transfers between facilities. Although people with MDR/RR-TB who are treated with adequate doses of appropriate drugs rapidly become non-infectious(43), there is increasing recognition that multiple patient transfers (often together with non-TB patients) and prolonged hospitalisation may expose both people with MDR/RR-TB and others to unnecessary infectious risks, reduce retention in care and incur financial costs to people with MDR/RR-TB (44,45). Studies in LMICs have shown that physical proximity of healthcare services can play an important role in the use of healthcare facilities(46–48).

People with MDR/RR-TB in this cohort presented with multiple medical comorbidities, including mental illness, and challenging social circumstances(37). Our exploration of the advantages and disadvantages of the different organisational patterns highlights the trade-offs when having to consider people with complex or severe disease. Since it is not possible to decentralise all components of care for people with complex MDR/RR-TB, flexibility to

For peer review only - http://bmjopen.bmj.com/site/about/guidelines.xhtml

provide differentiated or individualised care, which adapts to address the specific requirements of individuals or a subgroup of people with MDR/RR-TB, is particularly relevant (49) (20,22,50,51). For example, a flexible referral model like 'triangle', which utilises centralised clinical oversight to direct patient care, or implementation of multiple healthcare models (as observed in the Western Cape) may allow for a more seamless patient flow in response to the needs of people with MDR/RR-TB. Such flexible healthcare models, that can be organised in response to operational constraints and patient needs, may lead to contextually sensitive and optimal patient care that is safe, effective, person-centred, timely, efficient, and equitable (52–54). Although we did not collect data on patient costs, since centralised models were associated with longer hospital stays and travel distances, as well as fewer participants receiving an income, it is likely that patient costs were higher with centralised models. Fewer participants not receiving income fall into the line and dot patterns, which could point to these people with MDR/RR-TB being able to keep on with their employment and livelihoods. The elimination of catastrophic costs to families affected by TB is one of the key pillars of the End-TB strategy (55).

Care integration, and models that aim to overcome fragmentation between providers have been identified as important(56). Ideally, a bio-psychosocial systems approach should be used, where the primary focus of health care is the client in the context of their family(57) and where adult and paediatric services are provided together in a single setting(58). Reviews of family-centred care models in HIV care, indicate excellent adherence, retention in care, and low mortality and/or loss to follow-up in both adult and child services(59). Since HIV care in South Africa is highly decentralised, HIV status did not appear to affect the degree of decentralisation for people with MDR/RR-TB, even for people living with HIV who had low CD4

For peer review only - http://bmjopen.bmj.com/site/about/guidelines.xhtml

values. The dot model, where services are provided closest to the community is likely to be most family centred, but may be less ideal where home social circumstances are difficult, or where people with MDR/RR-TB require highly specialised care (60). In the latter circumstances, models, such as triangle, which are intrinsically more flexible, and responsive to changing patient needs, whilst containing a mix of care expertise, levels and disciplines may be more appropriate.

Societal features, such as urbanization, may have driven the emergence of certain models. However, we found no clear association between care patterns and epidemiological features of districts. For example, the most decentralised patterns (line and dot) were seen both in highly urbanised districts with large numbers of cases as well as in sparsely populated rural areas. These models appeared to be responses to differing pressures – the need to commence large numbers of people with MDR/RR-TB on treatment in urban areas, and the need to deliver care closer to people with MDR/RR-TB in remote areas. In rural districts in the Eastern Cape and KZN, where several of the more decentralised models were available, these appeared to be driven by clinician champions, as we have previously described(61). In the Western Cape, the pre-existing strength of the primary health care system may have facilitated the preponderance of line and dot models.

The WHO annual TB report card on South Africa cautions that reported numbers of people diagnosed and commenced on TB treatment in 2020 had fallen by 25% compared to the same period in 2019 and that gains in the TB program made over the past decade could be reversed by the COVID-19 pandemic(62). Countries have been encouraged to mitigate COVID-19

For peer review only - http://bmjopen.bmj.com/site/about/guidelines.xhtml

related impacts on MDR/RR-TB services by expanding use of digital technologies for remote advice and support, improving community connectivity(63), reducing the need for visits to health care facilities, encouraging home-based treatment and increasing use of strategies for facilitating patient drug supply. Lessons from the COVID-19 pandemic may offer lessons for organisation of MDR/RR-TB care delivery, in particular consideration of opportunities to improve connectivity to enhance decentralised care. We identified several examples where either telemedicine had been used, in line or dot models, or physicians travelled to enable peripheral facilities to initiate and care for newly-diagnosed or more people with complex illness. In some instances, medication prescriptions or clinical records travelled in place of people with MDR/RR-TB. However, these adaptations were not a formal system response but rather an example of local adaptation, often with staff using their own professional networks, personal devices and internet connectivity for communication(22). Description of patient movement patterns, critical appraisal of reasons for patient movement, and identification of which travel might be avoided through other equally effective means, such as virtual consultations, may be a useful exercise for diseases that are complex, rare or require specialised care (64–66).

Our study was undertaken prior to the widespread implementation of 6-month, all oral regimens for MDR/RR-TB (bedaquiline, pretomanid, linezolid and moxifloxacin [BPaLM]), which may affect decentralisation models further. It is possible that these relatively simpler regimens may facilitate further decentralisation. However, this is countered by the need for ECG monitoring for cardiotoxicity, together with the need to provide ongoing training and mentorship to the staff involved. We observed that drug stockouts were a problem at

For peer review only - http://bmjopen.bmj.com/site/about/guidelines.xhtml

decentralised sites – this may be a particular issue for more costly new drugs, due to concerns around shelf-life and wastage.

A limitation of our study is that it is not possible to clearly differentiate to what degree patient needs versus health system organisation and resources determined individual care pathways, but both are likely to have played a role. The substantial differences in care organisation, regardless of patient needs, reflect that there were multiple factors that were likely to determine care organisation. The sample size limited our ability to adequately assess care quality indicators, and so are unable to determine whether particular organisational models were associated with favourable patient outcomes. Further, we did not interview people with MDR/RR-TB, and so cannot draw conclusions about patient preferences. People with MDR/RR-TB who did not link to care, who died shortly after linking to care, and those linking through the private sector were not included in this study. Since the entry point to the study was a microbiological diagnosis, we are unable to quantify the effect of decentralised management on diagnostic delay. We were unable to determine changes to the degree of decentralisation and evolution of movement patterns during the study period.

In this analysis, we also did not specifically interrogate the reasons for the emergence of the different organisational models. Models of care are often based on what was in place historically, and over time become less responsive to the changing needs of the person with MDR/RR-TB and to health system constraints(67). Models of care are also often developed to bridge service delivery gaps rather than as a planned strategic response to an identified local

For peer review only - http://bmjopen.bmj.com/site/about/guidelines.xhtml

need (68,69). The development of models of care is commonly an iterative process(70,71), shaped by socio-political, economic, cultural, environmental, and legal drivers (72,73).

CONCLUSION

With the challenge of striving towards the WHO targets of zero deaths, disease and suffering due to TB by 2035 (74), stronger and more flexible health systems for decentralising patient management of MDR/RR-TB will be required to accommodate varying treatment needs. Indeed, a key finding of our study is that flexible models for offering decentralised care have emerged in different settings, in response to differing patient needs. The study of patient care pathways is a useful approach to providing a health systems typology of the ways in which care is organised for complex conditions. Having more clearly defined and articulated models of care may assist policy planners and implementers to identify which organisational models are most responsive to patient needs, quality indicators and system constraints. Increased use of digital technologies should be explored to democratise specialised and individualised care for MDR/RR-TB and other complex illnesses.

For peer review only

DECLARATIONS

Acknowledgements

The authors wish to thank the provinces of the W Cape, Eastern Cape, KwaZulu-Natal, and acknowledge the staff at the NHLS for their tremendous input and assistance. We give special mention to the late Dr Iqbal Masters and Mrs Anna Maria Evans for their contributions to the study. We also appreciate the support of Staff Nurse Cheryl Liedeman and Dr Widaad Zemanay.

Competing interests:

The authors declare that they have no competing interests.

Funding

This study was supported by a Health Systems Research Initiative award from the Medical Research Council of the United Kingdom and the Wellcome Trust (MR/N015924/1). This UK funded award is part of the EDCTP2 program supported by the European Union. HC is supported by a Wellcome Trust Fellowship. MN is supported by an Australian National Health and Medical Research Council Award (APP1174455). No conflicts of interest were reported.

Authors' contributions

Conception and design of study: MN, HC, MM, AG, LD and KK

Planning of the work: MN, HC, MM, AG, LD, KK, KM, JB, ML, NN, WJ

Interrogation and analysis of laboratory reports and geo-mapping: JH, LD, SLR, LM, MN, HC

For peer review only - http://bmjopen.bmj.com/site/about/guidelines.xhtml

Field visits and interviews: LD, SLR, WJ, JH, LM

Analysis of field worker and interview data: LD, SLR, WJ, JH, LM, KK, HC, MN

Conceptualizing manuscript: LD, MN

Drafting manuscript and revisions: LD, MN

Reviewing manuscript and approval of final version: MN, HC, MM, AG, LD, KK, KM, JB, ML, NN, JH, SLR, LM, WJ

For peer review only

For peer review only - http://bmjopen.bmj.com/site/about/guidelines.xhtml

Ethics approval and consent to participate

The study was a retrospective laboratory and record review of routinely captured data and a waiver of informed consent was granted to retrospectively review these records. Participants did not provide consent to participate in the study as all data analysed were collected as part of routine diagnosis and care. This study was approved by ethics boards at the University of Cape Town (350/2016) and the London School of Hygiene & Tropical Medicine (11680). Research approval was obtained in each province (Eastern Cape 2016RP30-232, KwaZulu-Natal 2016RP51-466, Western Cape 2016RP45-978).

Consent for publication

Not applicable

Availability of data and materials:

The dataset (anonymised participant data) used and analysed during the current study are not publicly available but can be obtained from the corresponding author (ORCID identifier 0000-0002-0020-1414) on reasonable request and may be shared through a controlled access repository.

Word count: 4670

For peer review only

Figure titles and legends:

Figure 1. Examples of the dominant geospatial movement patterns annotated on a stylised study district. Each coloured line depicts a single participant travelling in a pathway between the specific levels of health care facilities found in that model. Arrows represent direction of participant movement direction between health care facilities. Stars represent participants who only visited a single facility across their diagnostic and treatment pathway. A: 'Fan'; B: 'Chicken Foot'; C;' Hub and Spoke'; D: 'Triangle'; E: 'Line'; F: 'Dot'.

References

1. Loveday M, Mzobe YN, Pillay Y, Barron P. Figures of the dead: A decade of tuberculosis mortality registrations in South Africa. S Afr Med J. 2019;109(10):728–32.
2. Statistics South Africa. Mortality and causes of death in South Africa, 2016: Findings from death notification. Statistical release. 2016;(November 2016):1–127.
3. World Health Organization; Global tuberculosis report 2020 [Internet]. Geneva; 2020 [cited 2020 Jun 1]. Available from: https://www.who.int/teams/global-tuberculosis-programme/tb-reports/global-tuberculosis-report-2020
4. Murdoch J, Curran R, Rensburg AJ Van, Awotiwon A, Dube A, Bachmann M, et al. Identifying contextual determinants of problems in tuberculosis care provision in South Africa : a theory - generating case study. Infect Dis Poverty. 2021;1–13.
5. Sineke T, Evans D, Schnippel K, Van Aswegen H, Berhanu R, Musakwa N, et al. The impact of adverse events on health-related quality of life among patients receiving treatment for drug-resistant tuberculosis in Johannesburg, South Africa. Health Qual Life Outcomes. 2019;17(1).
6. World Health Organization. Consolidated Guidelines on Tuberculosis Treatment. World Health Organization; 2016. 99 p.
7. World Health Organization. Companion handbook. World Health Organization; 2014. 464 p.
8. IOM (Institute of Medicine). Patient Safety: Achieving a NewStandard for Care. Vol. 12, The National Academies Press. Washinton,DC; 2004. 126–129 p.
9. Law S, Daftary A, O'Donnell M, Padayatchi N, Calzavara L, Menzies D. Interventions to improve retention-in-care and treatment adherence among patients with drug-resistant tuberculosis: A systematic review. European Respiratory Journal. 2019;53(1):13993003.
10. Mohr E, Hughes J, Snyman L, Beko B, Harmans X, Caldwell J, et al. Healthcare delivery: Patient support interventions to improve adherence to drug-resistant tuberculosis treatment: A counselling toolkit. South African Medical Journal. 2015;105(8):631–4.

For peer review only

For peer review only - http://bmjopen.bmj.com/site/about/guidelines.xhtml

Hochman M, Asch SM. Disruptive Models in Primary Care: Caring for High-Needs, High-Cost Populations. J Gen Intern Med. 2017;32(4):392–7.

ReAct Group. Learning from Experiences : Introducing Bedaquiline in South Africa Executive summary [Internet]. 2020 [cited 2021 Nov 18]. Available from: https://www.reactgroup.org/news-and-views/news-and-opinions/year-2020/learning-from-bedaquiline-in-south-africa-comprehensive-health-systems-for-new-antibiotics/

Gray RM. The paradigms global plan of TB. Archetypal Explorations. 2020;108–28.

Stop TB Partnership. The Global Plan to End TB 2016-2020: The Paradigm Shift. World Health Organization Document. 2015;124.

Overland L, Uidelines C. Understanding the process to develop a Model of Care An ACI Framework. Perspectives on Swallowing and Swallowing Disorders (Dysphagia). 2009;20(3):60–4.

Loveday M, Wallengren K, Voce A, Margot B, Reddy T, Master I et al. Comparing early treatment outcomes of MDR-TB in decentralised and centralised settings in KwaZulu-Natal, South Africa. Int J Tuberc Lung Dis. 2012;16(2):209–15.

Padayatchi N, Friedland G. Decentralised management of drug-resistant tuberculosis (MDR- and XDR-TB) in South Africa: An alternative model of care. International Journal of Tuberculosis and Lung Disease. 2008;12(8):978–80.

World Health Organization. Guidelines for the programmatic management of drug-resistant tuberculosis Guidelines for the programmatic management of drug-resistant tuberculosis. World Health Organization. 2008;12–185.

National Department of Health South Africa. Multi-drug resistant tuberculosis: A policy framework on decentralised and deinstitutionalised management for South Africa. Pretoria; 2011.

National Department of Health South Africa. Multi-drug resistant tuberculosis a policy framework on decentralised and deinstitutionalised management for south africa. 2019.

Cox, HS; Dickson-Hall, L; Jassat W et al. Drug-resistant tuberculosis in South Africa: history, progress and opportunities for achieving universal access to diagnosis and effective treatment. In: South African Health Review 2017. 2017. p. 157–67.

Kielmann K, Dickson-Hall L, Jassat W et al. “We had to manage what we had on hand, in whatever way we could”: Adaptive responses in policy for decentralized drug-resistant tuberculosis care in South Africa. Health Policy Plan. 2021;36(3):249–59.

Kielmann K, Dickson-Hall L, Jassat W, Le Roux S, Moshabela M, Cox H, et al. “We had to manage what we had on hand, in whatever way we could”: Adaptive responses in policy for decentralized drug-resistant tuberculosis care in South Africa. Health Policy Plan. 2021;36(3):249–59.

WHO. Implementing The End TB Strategy. Antimicrob Agents Chemother. 2015;58(12):7250–7.

The Global Fund. Best Practices on TB Case Finding and Treatment. The global fund org. 2018;73.

Cott CA, Davis AM, Badley EM, Wong R, Canizares M, Li LC, et al. Commonalities and differences in the implementation of models of care for arthritis: Key informant interviews from Canada. BMC Health Serv Res. 2016;16(1):1–12.

For peer review only - http://bmjopen.bmj.com/site/about/guidelines.xhtml

World Health Organization; Everybody's business strengthening health systems to improve health outcomes. Geneva; 2007.

Saha, S, MD, Beach, MC, Cooper L. Patient Centeredness, Cultural Competence and Healthcare Quality. 2010;100(11):1275–85.

National Department of Health South Africa. White paper for the transformation of the health system in South Africa. 1997.

Walt G, Shiffman J, Schneider H, Murray SF, Brugha R, Gilson L. "Doing" health policy analysis: Methodological and conceptual reflections and challenges. Health Policy Plan. 2008;23(5):308–17.

Hill J, Dickson-Hall L, Grant AD, Grundy C, Black J, Kielmann K, et al. Drug-resistant tuberculosis patient care journeys in South Africa: A pilot study using routine laboratory data. International Journal of Tuberculosis and Lung Disease. 2020;24(1):83–91.

Stokes J, Man MS, Guthrie B, Mercer SW, Salisbury C, Bower P. The foundations framework for developing and reporting new models of care for multimorbidity. Ann Fam Med. 2017;15(6):570–7.

Sijm-Eeken, M, Zheng J, Peute L. Towards a Lean Process for Patient Journey Mapping - A Case Study in a Large Academic Setting. Stud Health Technol Inform. 2020;270:1071–5.

Hanson CL, Osberg M, Brown J, Durham G, Chin DP. Conducting Patient-Pathway Analysis to Inform Programming of Tuberculosis Services: Methods. Journal of Infectious Diseases. 2017;216(Suppl 7):S679–85.

Rathi P, Shringarpure K, Unnikrishnan B, Pandey A, Nair A. Patient treatment pathways of multidrug-resistant tuberculosis cases in coastal South India: Road to a drug resistant tuberculosis center. F1000Res. 2019;8:498.

Loveday M, Wallengren K, Reddy T, Besada D, Brust JCM, Voce A, et al. MDR-TB patients in KwaZulu-Natal, South Africa: Cost-effectiveness of 5 models of care. PLoS One. 2018;13(4):1–13.

Mitrani L, Roux S Le, Hill J, Loveday M, Grant AD, Kielmann K, et al. Diverse clinical and social circumstances: developing patient-centred care for DR-TB patients in South Africa L. Public Health Action. 2021;I(3):120–5.

Citro B, Soltan V, Malar J, Katlholo T, Smyth C, Sari AH, et al. Building the Evidence for a Rights-Based , People- Centered , Gender-Transformative Tuberculosis Response : An Analysis of the Stop TB Partnership Community , Rights , and Gender Tuberculosis Assessment. Health and Human Rights Journal. 2021;23(2):253–67.

Moshabela, M, Cox, H., Jassat, W., Dickson-Hall, L., Kielmann, K., Loveday M., Black, J., Mlisana, K., Nicol, M., Grant A. "We were caught sleeping": Tensions between 'control-based' and 'access-based' approaches in the formulation of a national policy to decentralise management of drug-resistant tuberculosis in South Africa. Manuscript under review, BMJ Global Health.

Furin J, Loveday M, Hlangu S, Dickson-hall L, Le Roux S, Nicol M, et al. " A very humiliating illness " : a qualitative study of patient-centered Care for Rifampicin-Resistant Tuberculosis in South Africa. 2020;1–11.

Loveday M, Wallengren K, Brust J, Roberts J, Voce A, Margot B et al. Community-based care vs. centralised hospitalisation for MDR-TB patients, KwaZulu-Natal, South Africa. Int J Tuberc Lung Dis. 2015;19(2):163–71.

For peer review only

For peer review only - http://bmjopen.bmj.com/site/about/guidelines.xhtml

Loveday M, Wallengren K, Voce A, Margot B, Reddy T, Master I, et al. Comparing early treatment outcomes of MDR-TB in decentralised and centralised settings in KwaZulu-Natal, South Africa. International Journal of Tuberculosis and Lung Disease. 2012;16(2):209–15.

Petersen E, Khamis F, Battista G, Glerup J, Marais B, Wejse C, et al. International Journal of Infectious Diseases De-isolation of patients with pulmonary tuberculosis after start of treatment — clear , unequivocal guidelines are missing. International Journal of Infectious Diseases. 2017;56:34–8.

Shringarpure KS, Isaakidis P, Sagili KD, Baxi RK, Das M, Daftary A. "When treatment is more challenging than the disease": A qualitative study of MDR-TB patient retention. PLoS One. 2016;11(3):1–12.

Daftary A, Mondal S, Zelnick J, Friedland G, Seepamore B, Boodhram R, et al. Dynamic needs and challenges of people with drug-resistant tuberculosis and HIV in South Africa : a qualitative study. Lancet Glob Health. 2015;9(4):e479–88.

Al-mandhari A, Al-adawi S, Al-zakwani I, Al-shafaee M, Eloul L. Impact of Geographical Proximity on Health Care Seeking Behaviour in Northern Oman. 2008;8(3):310–8.

Peabody, JW, Taguiwalo, MM, Robalino, DA, Frenk J. Improving the Quality of Care in Developing Countries. In: Disease Control Priorities in Developing Countries. 2nd ed. New York: Oxford University Press; 2006.

Mangundu M, Roets L, Rensberg EJ Van, Africa S, Mangundu M. Accessibility of healthcare in rural Zimbabwe : The perspective of nurses and healthcare users. :1–7.

The Global Fund. The Global Fund. 2015 [cited 2021 Oct 20]. A Toolkit for Health Facilities Differentiated Care For HIV and Tuberculosis. Available from: https://reliefweb.int/report/world/toolkit-health-facilities-differentiated-care-hiv-and-tuberculosis

Benn J, Burnett S, Parand A, Pinto A, Iskander S, Vincent C. Studying large-scale programmes to improve patient safety in whole care systems: Challenges for research. Soc Sci Med. 2009 Dec 1;69(12):1767–76.

Harrison MB, Legare F, Graham ID, Fervers B. Adapting clinical practice guidelines to local context and assessing barriers to their use. CMAJ. 2010;182(2):78–84.

Hodkinson P, Argent A, Wallis L, Reid S, Perera R, Harrison S, et al. Pathways to care for critically Ill or injured children: A cohort study from first presentation to healthcare services through to admission to intensive care or death. PLoS One. 2016;11(1).

Das M, Mathur T, Ravi S, Meneguim AC, Iyer A, Mansoor H, et al. Challenging drug-resistant TB treatment journey for children, adolescents and their care-givers: A qualitative study. PLoS One. 2021;16(3 March):1–15.

Seddon JA, Hesseling AC, Godfrey-Faussett P, Schaaf HS. High treatment success in children treated for multidrug-resistant tuberculosis: An observational cohort study. Thorax. 2014;69(5):471–7.

World Health Organization. WHO | WHO End TB Strategy. World Health Organization. 2015;

World Health Organization. Integrated care models: an overview. Health Services Delivery Programme. 2016;42.

Epstein RM, Legacy GES. The Biopsychosocial Model 25 Years Later : 2004;576–82.

For peer review only - http://bmjopen.bmj.com/site/about/guidelines.xhtml

Babiker A, Husseini M El, Nemri A Al, Frayh A Al, Juryyan N Al, Faki MO, et al. Current Opinion Health care professional development : Working as a team to improve patient care. 2014;14(2):9–16.

Leeper SC, Montague BT, Friedman JF, Flanigan TP. Lessons learned from family-centred models of treatment for children living with HIV: Current approaches and future directions. J Int AIDS Soc. 2010;13(SUPPL. 2).

Le Roux SR, Jassat W, Dickson L, Mitrani L, Cox H, Mlisana K, et al. The role of emergent champions in policy implementation for decentralised drug-resistant tuberculosis care in South Africa. BMJ Glob Health. 2022 Dec 9;7(12).

Le Roux, Sacha, Jassat, Waasila, Dickson-hall, Lindy, Mitrani, Leila, Cox, Helen, Milsana K, et al. The role of healthcare champions in policy implementation for decentralised drug-resistant tuberculosis care in South Africa. Manuscr under Rev BMJ Global Health.

Chin DP, Hanson CL. Finding the Missing Tuberculosis Patients. Journal of Infectious Diseases. 2017;216:S675–8.

Chin DP, Hanson CL. Finding the Missing Tuberculosis Patients. Journal of Infectious Diseases. 2017;216(Suppl 7):S675–8.

Vaikuntam BP, Middleton JW, Mcelduff P, Pearse J, Walsh J, Cameron ID, et al. Assessing the impact of care pathways on potentially preventable complications and costs for spinal trauma patients : protocol for a data linkage study using cohort study and administrative data. 2018;10–4.

Sharwood LN, Whyatt D, Vaikuntam BP, Cheng CL, Noonan VK, Joseph AP, et al. A geospatial examination of specialist care accessibility and impact on health outcomes for patients with acute traumatic spinal cord injury in New South Wales , Australia : a population record linkage study. 2021;4:1–13.

Moscibrodzki P, Parkinson, S, Ferry R, Nwaneri, N, Thies W. Mapping the technology landscape of national TB programs. Geneva; 2021.

World Health Organization; Monitoring the building blocks of health systems : A handbook of indiators and their measurement strategies. Geneva; 2010.

World Health Organization, Organisation for Economic Co-operation and Development TWB. Delivering quality health services: A global imperative for universal health coverage. Geneva; 2018.

Ramsden R, Davies S, Richard MPH, Ba C, Service H, Shannon M, et al. Collaborative care : Primary health workforce and service delivery in Western New South Wales — A case study. 2021;(July):768–78.

Taber JM, Ph D, Leyva B, Persoskie A, Ph D. Why do People Avoid Medical Care ? A Qualitative Study Using National Data. 2008;18–24.

Kruk ME, Gage AD, Arsenault C, Jordan K, Leslie HH, Roder-dewan S, et al. The Lancet Global Health Commission High-quality health systems in the Sustainable Development Goals era : time for a revolution. 2018;6(November):1196–252.

World Health Organization. Action on the Social Determinants of Health: Learning from Previous Experiences. Social Determinants of Health Discussion Paper 1. 2010.

For peer review only

For peer review only - http://bmjopen.bmj.com/site/about/guidelines.xhtml

Dookie S, Singh S. Primary health services at district level in South Africa: a critique of the primary health care approach. BMC Fam Pract. 2012;13:67.

World Health Organization. Global Tuberculosis Report 2015. Geneva; 2015.

For peer review only - http://bmjopen.bmj.com/site/about/guidelines.xhtml

Pattern name	Fan	Chicken foot	Hub & spoke	Triangle	Line	Dot
**Level of decentralisation**	Highly centralised care	Care decentralised to district level with high reliance on COE	Care decentralised to district level	Multi-level / multi-facility decentralised care	Community based, ambulatory care	Community based, ambulatory care
**Description of care delivery**	Hospital based care with long admissions, far from home.	Hospital based care with long admissions, some care closer to home.	Hospital based care with long admissions, closer to home.	Flexible care, coordinated by specialist doctors.	Home based care, with short admissions if required.	Home based, outpatient care.
**Diagnosis**	At clinic or general☐ hospital.	At clinic or general☐ hospital	At clinic or general hospital	At clinic or general☐ hospital	At clinic	At clinic
**Treatment initiation**	At provincial COE	At specialist TB hospital, but with frequent change to regimen at COE.	At specialist TB hospital	At specialist TB hospital or by specialist TB outreach service.	At MDR/RR-TB clinic or district hospital MDR/RR-TB clinic	At the diagnosing clinic
**Co-ordination of care and follow up**	Specialist TB doctors at the COE	Specialist TB doctors at the COE and TB doctors at the decentralised site.	TB doctors at the specialised site	Specialist physician or TB physician on site or through telemedicine.	TB nurses at clinics. Sometimes assisted by a generalist doctor.	TB nurses at clinics. Sometimes assisted in person/telephonically by generalist doctor.
**Hospitalisation**	At COE, frequently for long periods.	At specialist TB hospital or COE, frequently for long periods.	At specialist TB hospital for long periods.	Depending on patient's medical and social circumstances.	Depending on patient's medical and social circumstances.	No admission.
**Number of participants**	N=41	N=19	N=72	N=30	N=12	N=17

For peer review only

	(urban n=25, rural n =16)	(urban n=5, rural n=14)	(urban n=29, rural n=43)	(urban n=13, rural n=17)	(urban n=4, rural n=8)	(urban n=10, rural n=7)
**Median distance from home to diagnosing site****	10km (n=36)	11.4km (n=17)	14.6km (n=66)	10.6km (n=24)	5.4km (n=6)	0.9km (n=14)
**Median distance from home to initiating site****	44km (n=36)	38km (n=17)	41km (n=66)	26km (n=24)	6km (n=6)	2km (n=14)
**Median distance travelled between facilities**	102 km	188km	51km	76km	8km	0km
**Median time to treatment initiation*****	35 days	13 days	14 days	7 days	0 days	1 day
**Median (range) number of trips******	6 (1-7)	5 (2-12)	6 (1-17)	8 (2-16)	6 (1-21)	8 (6-22)
**Median duration of hospital admission**	79 days (IQR=105) Range 0 - 240	103 days (IQR=91) Range 20 - 197	81 days (IQR=124 Range 0 - 281	40 days (IQR=57) Range 0 - 175	12 days (IQR=24) Range 0 - 38	0 days (IQR=0) Range:0 - 0
**Vital status 6 months following MDR/RR-TB lab result**	Deceased 20% (8)	Decreased 11% (2)	Deceased 25% (18)	Deceased 20% (6)	Deceased 33% (4)	Deceased - Nil

For peer review only - http://bmjopen.bmj.com/site/about/guidelines.xhtml

For peer review only

**Treatment status at 24 months following MDR/RR-TB lab result**	Completed treatment 45% (19)<br>Still on treatment 21% (9)<br>*(favourable 68%)*<br>Deceased 24% (10)<br>Lost to follow-up 5% (2)<br>Treatment failure Nil<br>Unknown 3% (1)	Completed treatment 33% (6)<br>Still on treatment 16.5% (3)<br>*(favourable 47%)*<br>Deceased 16.5% (3)<br>Lost to follow-up 37% (7)<br>Treatment failure Nil<br>Unknown Nil	Completed treatment 41% (29)<br>Still on treatment 21% (15)<br>*(favourable 61%)*<br>Deceased 26% (19)<br>Lost to follow-up 9% (6)<br>Treatment failure 2% (1)<br>Unknown 3% (2)	Completed treatment 53% (16)<br>Still on treatment 17% (5)<br>*(favourable 70%)*<br>Deceased 20% (6)<br>Lost to follow-up 10% (3)<br>Treatment failure Nil<br>Unknown Nil	Completed treatment 17% (2)<br>Still on treatment 42% (5)<br>*(favourable 58%)*<br>Deceased 42% (5)<br>Lost to follow-up Nil<br>Treatment failure Nil<br>Unknown Nil	Completed treatment 94% (16)<br>Still on treatment Nil<br>*(favourable 94%)*<br>Deceased Nil<br>Lost to follow-up 6% (1)<br>Treatment failure Nil<br>Unknown Nil

* n = number of participants ** distance travelled calculated as straight-line distance as calculated from the patient's home to the health care site. *** Days from MDR/RR-TB diagnosis to treatment initiation

**** Number of trips undertaken to health care sites during first 9 months of treatment. ☐General hospital indicates either a district or regional level hospital. COE – TB Centre of Excellence

<u>Table 1: Dominant geospatial movement patterns observed within study districts, together with key features of organisation of care within these patterns, descriptive statistics of patient journeys and treatment status after 24 months.</u>

peer review only

Province	Rural or urban	Districts	A<br>Fan	B<br>Chicken foot	C<br>Hub & Spoke	D<br>Triangle	E<br>Line	F<br>Dot
**KwaZulu -Natal**	Urban	Ethekwini	4	3	5	1		1
		Uthungulu		4	2	7		2
	Rural	Ilembe*	2		8	5		
		Umkanyakude		2	7	6		
		Uthukela		6	8	1		
**Western Cape**	Urban	City of Cape Town	5	2			3	5
		Eden			12	2		1
	Rural	Cape Winelands			8	2	2	3
		West Coast	1		9		2	2
**Eastern Cape**	Urban	Buffalo City	14					
		Nelson Mandela Bay			10	3	1	1
	Rural	OR Tambo	8		1	1	4	1
		Sarah Baartman	7	2	2	2		1
		**TOTAL (n=191):**	**41**	**19**	**72**	**30**	**12**	**17**

iLembe* – undergoing rural to urban transition

Numbers in cells refer to number of individual patient pathways observed for each organisational model within the district

Table 2: The distribution of organisational models by study district.

For peer review only - http://bmjopen.bmj.com/site/about/guidelines.xhtml

PATIENT FEATURES	Category	Fan<br>*n=41	Chicken foot<br>n=19	Hub & spoke<br>n=72	Triangle<br>n=30	Line<br>n=12	Dot<br>n=17	Total<br>n=191
Age category (yrs)	0-19 yrs	3 (7)	1 (5)	3 (4)	2 (7)	0	0	9 (5)
	20-59 yrs	38 (93)	18 (95)	65 (90)	27 (90)	12 (100)	17 (100)	177 (92)
	≥60 yrs	0	0	4 (6)	1 (3)	0	0	5 (3)
Not receiving income		30 (73)	14 (74)	49 (68)	18 (60)	4 (33)	9 (53)	124 (65)
HIV positive		29 (71)	16 (84)	50 (70)	18 (60)	11 (92)	7 (41)	131 (69)
Baseline CD4 <200 c/ul**	CD4 <200 c/ul	14 (48)	6 (38)	15 (30)	5 (26)	5 (45)	2 (29)	47 (35)
	CD4≥200 c/ul	9 (31)	5 (31)	17 (34)	6 (33)	3 (27)	3 (43)	43 (33)
	Unreported CD4	6 (21)	5 (31)	18 (36)	7 (39)	3 (27)	2 (29)	41 (32)
Previous TB history	DS-TB any episode	25 (61)	14 (74)	49 (68)	19 (63)	6 (50)	8 (47)	121 (63)
	MDR/RR-TB 1 episode	3 (7)	4 (21)	6 (8)	3 (10)	3 (25)	1 (6)	20 (10)
	MDR/RR-TB ≥2 episodes	5 (12)	1 (5)	9 (13)	1 (3)	0	0	16 (9)
Baseline BMI <18.5 kg/m2	≤18.5 kg/m2	10 (24)	7 (37)	25 (35)	7 (23)	5 (42)	4 (24)	58 (49)
	≥18.6 kg/m2	19 (47)	8 (42)	26 (36)	11 (34)	0 (0)	9 (52)	74
	Unreported BMI	12 (29)	4 (21)	21 (29)	12 (40)	7 (58)	4 (24)	60 (51)
Comorbidities per person (excluding HIV)	1-3 comorbidities/pt	4 (10)	4 (21)	15 (20)	10 (33)	2 (17)	2 (12)	37 (19)
	> 3 comorbidities/pt	0	0	2 (3)	1 (3)	4 (33)	2 (12)	9 (5)
2nd line TB drug resistance	MDR/RR-TB	22 (53)	11 (58)	46 (64)	22 (73)	9 (76)	12 (70)	122 (64)
	Pre – XDR	11 (27)	6 (32)	8 (11)	4 (13)	1 (8)	1 (6)	31 (16)
	XDR	6 (15)	1 (5)	8 (11)	2 (7)	1 (8)	1 (6)	19 (10)
	Unknown resistance	2 (5)	1 (5)	10 (14)	2 (7)	1 (8)	3 (18)	19 (10)

*Number of patients **Within 3 months of diagnosis % refer to proportion of patients within each pattern

For peer review only - http://bmjopen.bmj.com/site/about/guidelines.xhtml

775 Table 3: Characteristics of the study participants n=191 within each organisational pattern
776 that may influence the degree of decentralisation achieved.

For peer review only

For peer review only - http://bmjopen.bmj.com/site/about/guidelines.xhtml

PATTERN NAME	Fan	Chicken foot	Hub & spoke	Triangle	Line	Dot
**Advantages of the organisational model for people with MDR/RR-TB**	Access to multidisciplinary team with experienced clinicians, drug availability.<br><br>Access to acute tertiary (ICU, dialysis) and chronic tertiary care services (nephrology, cardiology, neurology).	People with less complex illness are able to be cared for in their districts.<br><br>Ready referral pathway back to the centralised site for people with complex illness.	People are cared for in their districts, closer to home .<br><br>Specialist TB hospitals may be better designed for long-term care than COEs. Staff are more familiar with individual people and their conditions and contexts, compared to COE.	People able to access care at different facilities, depending on preference.<br><br>As a collective, the facilities have multidisciplinary resources.<br><br>Expert conductor has the ability to navigate care and advocate for holistic care. Oversight for people with complex conditions.	Care close to home.<br><br>Initial period of supervised inpatient care may provide nutrition support, side effect monitoring, linkage to financial grant support & patient education.	Care very close to home<br><br>Increased focus on community, patient- and family-centred care, with staff embedded within the community<br><br>Other decentralised services, eg, HIV care, family planning, are easily accessible.
**Advantages of the organisational model for the health system**	Logistically simple to compile statistics & monitor people with MDR/RR-TB and train specialised staff.<br><br>Rapid response to change in diagnostic or drug regimens.<br><br>Centralization of expensive drug storage & control – minimise waste, expiry or	Reduces bed pressure on centralised sites, whilst maintaining strong clinical governance of people with complex illness.<br><br>Less reliance on one facility per province (redundancy).	Strong connections between specialist TB hospital and clinics facilitates patient monitoring and surveillance.<br><br>The district node taking a high degree of responsibility promotes accountability.<br><br>Early treatment modification by TB specialists at the	Oversight from an expert conductor facilitates rapid adjustment of patient care, reducing hospitalization and optimising resource usage.<br><br>Leverage of resources by senior clinicians.<br><br>Flexibility around staffing	Outpatient review within the community, with option for short hospital admission for treatment optimisation and stabilisation.<br><br>Reduces patient transport (infection transmission, expense) and promotes family support, whilst minimising the need to provide services and drugs to	Rapid diagnosis and treatment initiation.<br><br>Reduced requirement for patient transport.<br><br>Patient trust in local staff promotes adherence and treatment completion.

For peer review only - http://bmjopen.bmj.com/site/about/guidelines.xhtml

	shrinkage.		district level reduces admissions.	allows system to buffer more easily.	every clinic.	Support from decentralised HIV programmes.
**Challenges of the organisational model for people with MDR/RR-TB**	Treatment delay, long admission times, hospital acquired infection, poor family centredness, loss of patient income.<br><br>Community stigma of being at a CoE for 'strong' TB. Disconnect from community chronic care services.	People with MDR/RR-TB need to adjust to care in dfferent hospitals.<br><br>Fragmentation of care<br><br>Travel between sites.<br><br>Moderate to long admission times.	District sites may have reduced support from COEs. This may result in difficulty referring people with MDR/RR-TB requiring more complex care.<br><br>Moderate to long admission times.	Care may be fragmented between the sites & people may become lost to follow-up between sites.<br><br>Requires support from the province (travel, information technology services, training) which may not be consistent.	Sites may have reduced support from COEs. This may result in difficulty referring people requiring more complex care.<br><br>Multidisciplinary team not always available. Complications may go undetected or there may be a delay in referring for higher levels of care.	Sites may have reduced support from COEs. This may result in difficulty referring people requiring more complex care.<br><br>Multidisciplinary team less available. Complications may go undetected or delay in referral for higher-level care.
**Challenges of the organisational model for the health system**	Reliance on a single centre - lack of redundancy.<br><br>Skill set 'owned' by a few individuals at the COE's. Other HCWs see themselves as unable to care for MDR/RR-TB reducing willingness to care for these people.	Over-reliance on COE, with decentralised site poorly empowered to manage people with MDR/RR-TB .<br><br>Providing safe patient transport.	Depending on the geographical size of the province, many people with MDR/RR-TB still travel far distances to visit the TB service or be admitted to hospital.	Reliance on retention of individual specialist physicians to maintain the program.<br><br>Tracking patient progress through the system.<br><br>Providing safe transport.	Monitoring & supporting many MDR-TB sites. Providing sites with laboratory & IT infrastructure, drugs, trained staff and supervision.<br><br>Ensuring quality of care provision and timely capturing of accurate data.	Monitoring & supporting many MDR-TB sites. Providing sites with laboratory & IT infrastructure, drugs, trained staff, and supervision.<br><br>Ensuring quality of care provision and timely capturing of accurate data.

For peer review only - http://bmjopen.bmj.com/site/about/guidelines.xhtml

Table 4. Advantages and challenges associated with each organisational care pattern

For peer review only

For peer review only - http://bmjopen.bmj.com/site/about/guidelines.xhtml

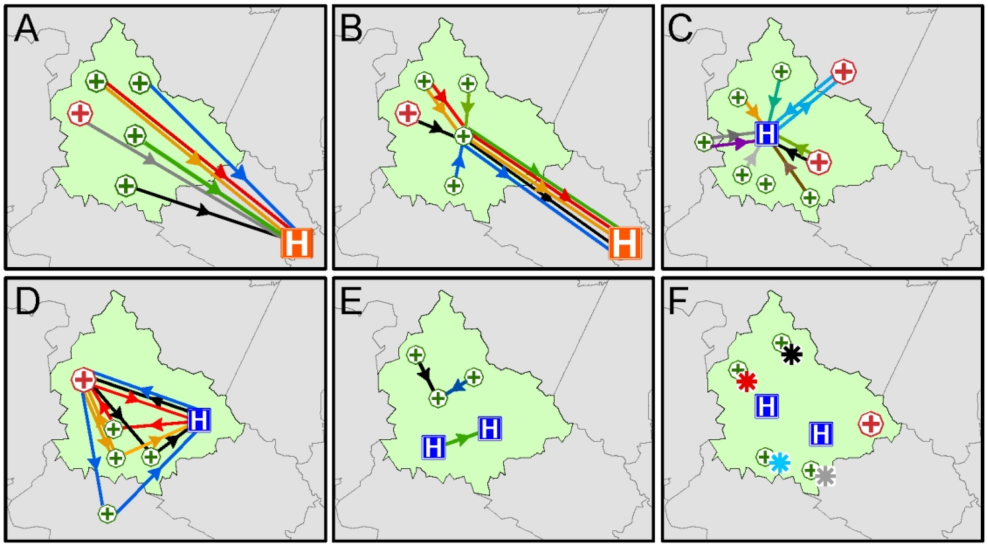

Figure 1. Examples of the dominant geospatial movement patterns annotated on a stylised study district. Each coloured line depicts a single participant travelling in a pathway between the specific levels of health care facilities found in that model. Arrows represent direction of participant movement direction between health care facilities. Stars represent participants who only visited a single facility across their diagnostic and treatment pathway. A: ’Fan’; B: ‘Chicken Foot’; C;’ Hub and Spoke’; D: ‘Triangle’; E: ‘Line’; F: ’Dot’.

210x148mm (200 x 200 DPI)

Supplementary material: A

Additional information related to COREQ (Consolidated criteria for Reporting Qualitative research) Checklist

Domain 1: Research team & reflexivity

LD, JH, SLR and LM conducted interviews. The researchers were a team of clinicians and social science degree majors qualified in the fields of medicine, biology and teaching. At the time of the study all were full time researchers on the project. Three were female and one was male. Three were South African and one was Australian. Three had Masters degrees and one had a Bachelor's degree. LD had been employed as a clinician in the DR-TB, HIV and family medicine programs in KZN and the Western Cape and had been previously working on a DR-TB linkage to care study. JH had been employed as a clinician in the DR-TB and infectious diseases domain at Papua New Guinea. SLR had been teaching senior high school students and working in a health care call centre and medical practice previously. LM had been completing a master's degree in biology involving geo-informatics. LD and JH had been investigators in previous DR-TB studies. There was no formal relationship established prior to the study, except some familiarity between the local DR-TB clinicians and LD. The participants were aware of research aims and objectives and the names of the researchers and investigators. The participants were aware that the researchers were interested in health systems around DR-TB disease in SA.

Domain 2: Study design

Questions and guides were piloted with a smaller group of local DR-TB staff at a non-study facility and then provided to participants by the interviewers, together with patient journey maps obtained from that region to serve as probes for some questions. Audio recordings were obtained, except in the case of a correctional services facility where recording was not permitted. A single round of interviews took place at the work place during tea or lunch breaks. Field notes were made during and then more comprehensively after the interview. The duration of the interviews ranged from 30-50 minutes, and depended on the operational work time constraints and the degree of insight and knowledge held and shared by the participant. Data saturation was discussed before ending interviews. Transcripts were not returned for comment or correction.

Domain 3: Analysis and findings

SLR and LD reviewed the interviews and coded the data, using a simple, unstructured coding scheme to extract information on the implications of the patterns of care for patients or the health care system . Participants did not give feedback on the data, except at an end-of-study feedback session with the Departments of Health, which was not used to inform this manuscript. Participant quotations were not presented. There was consistency between the data presented and the findings. Major themes were clearly presented in the findings. The nature of the patient pathway analysis allowed for interrogation of both major themes (common reasons for patient movement) as well as minor themes less common reasons for patient movement).

For peer review only - http://bmjopen.bmj.com/site/about/guidelines.xhtml

Supplementary information: B

		Rural or urban	Demographic and health care descriptors					Poverty indices				Death and disease profile						
			Population count	Population density / km^2	Number of clinics	Number of public hospitals	Provincial spend on health / person / year (ZAR)*	Population below lower poverty line	Unemployed	Informal shack dwellings	Illiteracy	Life expectancy (yrs)	% of deaths due to infectious diseases	Leading cause of natural death	HIV / TB coinfection	TB incidence / 100 000 in 2015	Number of MDR/RR-TB cases in 2017**	
																	XDR	MDR
PROVINCE	KWAZULU-NATAL						R3 527	44%	28%		15%	57-64			69%	685	115	1687
DISTRICT	eThekwini (1)***	City (metro)	3 702 231	1502	31	18		41%	19%	13%	17%		16%	Heart disease		698	85	1063
	uThungulu (2)	City (small)	971 135	88	83	9		72%	34%	3%	12%		21%	TB		859	7	242
	iLembe (3)	Rural (small metro)	657 612	185	36	8		55%	31%	9%	15%		24%	TB		801	10	151
	uMkhanyakude (4)	Rural district	689 090	61	86	5		80%	31%	15%	25%		27%	HIV/ AIDS		577	6	146
	uThukela(5)	Rural district	706 588	63	49	4		63%	29%	1%	10%		24%	TB		533	7	85
PROVINCE	WESTERN CAPE						R3 867	32%	20%		7%	66-71			47%	681	86	1140
DISTRICT	City of Cape Town (6)	City (metro)	4 208 000	1530	130	18		31%	22%	11%	2%		14%	Diabetes		596	59	757
	Eden (7)	City (metro)	611 279	25	209	7		33%	27%	8%	12%		17%	Diabetes		809	5	100
	Cape Winelands (8)	Rural district	917 462	40	134	10		34%	17%	7%			18%	Diabetes		880	14	170
	West Coast (9)	Rural district	436 403	15	180	7		30%	19%	8%	13%		16%	Heart disease		837	8	113
PROVINCE	EASTERN CAPE						R3 046	56%	47%		18%	60-67			56%	692	281	1151
DISTRICT	Buffalo City (7)	City (metro)	893 157	300	103	10		41%	35%	25%	12%		21%	TB		743	77	314
	Nelson Mandela Bay(8)	City (metro)	1 263 051	615	68	12		51%	63%	7%	1%		20%	Diabetes		938	150	471
	OR Tambo (5)	Rural district	1 457 384	108	147	16		67%	39%	1%	14%		20%	TB		571	22	220
	Sarah Baartman (10)	Rural district	520 480	8	89	15		41%	30%	9%	13%		20%	TB		1022	32	146

Table S1. Description of key health care and demographic features of the study districts

* ZAR = South African Rands (1 US dollar is approximately 17 ZAR)

** Data from National Institute of Communicable Diseases

***Reference numbers for data sources are given following each district name

For peer review only - http://bmjopen.bmj.com/site/about/guidelines.xhtml

Disease	Definition	Programmatic treatment for MDR/RR-TB in South Africa
DR-TB	Drug resistant TB<br>(Resistance to any TB drug)	Second-line treatment for 18–24 months, with a combination including a combination selection with several of:<br>• Fluoroquinolones, e.g.: ofloxacin, moxifloxacin, levofloxacin<br>• Second-line injectable drugs, e.g.: amikacin, kanamycin, capreomycin<br>• Other available drug, e.g.: ethionamide, terizidone, para-aminosalicyclic acid<br>• Repurposed drugs, e.g.: linezolid, clofazimine<br>• New drugs recently available, e.g.: bedaquiline, delamanid
MDR-TB	Multi drug resistant TB<br>(Resistance to isoniazid and rifampicin)	
RR-TB	Rifampicin resistant TB<br>(Resistant to at least rifampicin, regardless of resistance to other drugs)	
PreXDR-TB	Pre-Extensively drug resistant TB<br>(MDR-TB with resistance to either a fluoroquinolone OR a second-line injectable TB drug)	
XDR-TB	Extensively drug-resistant TB<br>(MDR-TB with resistance to both a fluoroquinolone AND a second-line injectable TB drug)	
Completed treatment	Treatment completed as recommended by the national policy without evidence of failure *but no record that* three or more consecutive cultures ≥30 days apart are negative after the intensive phase.	
Cured	Treatment completed as recommended by the national policy without evidence of failure *and* three or more consecutive cultures ≥30 days apart are negative after the intensive phase.	

Table S2. Definitions and treatment for drug-resistant TB at the time of the study

For peer review only

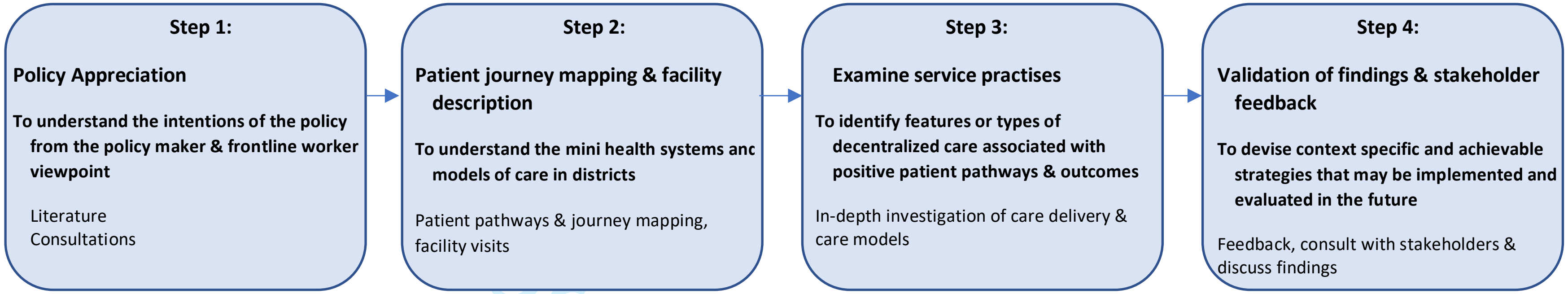

Supplementary Figure 1. The overarching research project, within which this sub-study was nested. This analysis relates to Step 2 and Step 3 of the project.

For peer review only - http://bmjopen.bmj.com/site/about/guidelines.xhtml

Supplementary Figure 2: South African map showing thirteen districts selected for study in three provinces. Districts are shaded according to Province (shaded darker green in Western Cape, lighter green in Eastern Cape and orange in KwaZulu-Natal).

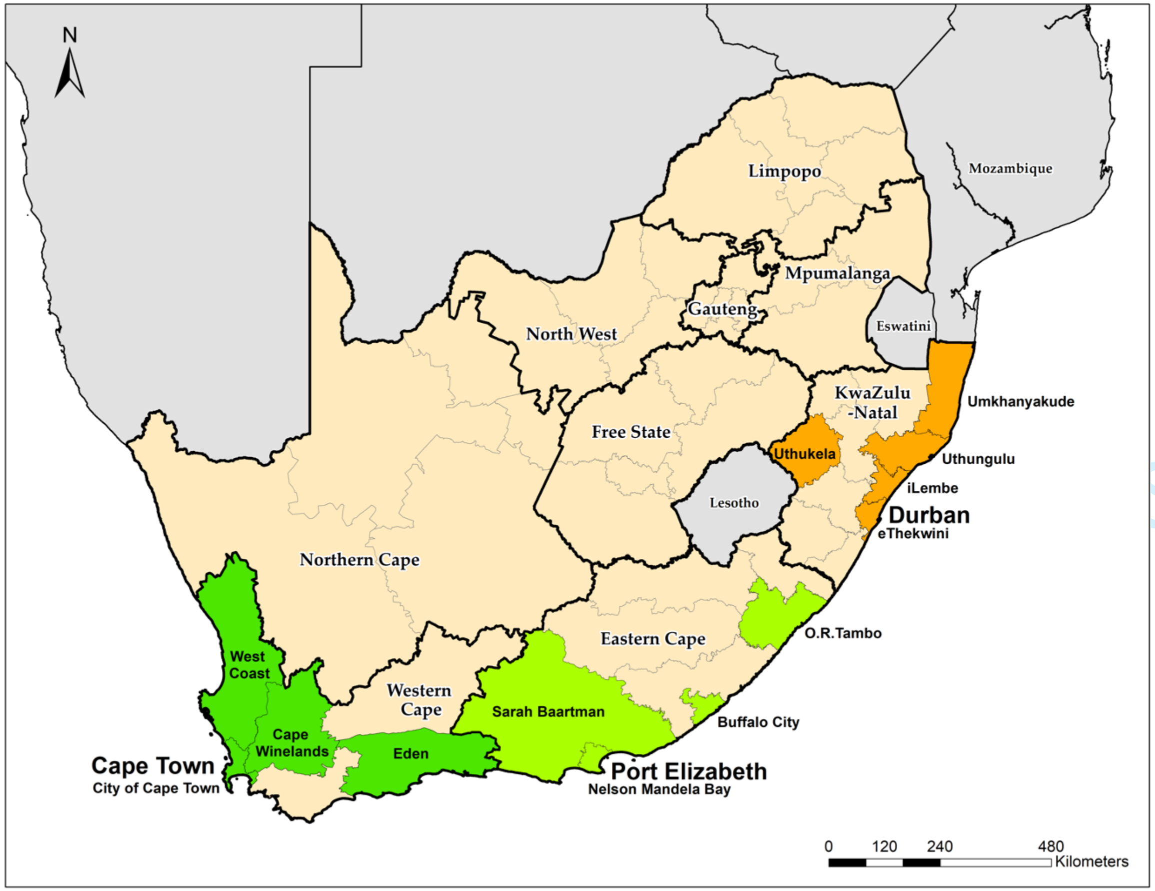

References:

1. National Department of Cooperative Governance & Traditional Affairs South Africa. Profile and Analysis : District Developement Model. 2020. p. 31 Ethekwini Metropolitan KZN. Available from: https://www.cogta.gov.za/ddm/wp-content/uploads/2020/07/Metro-Profile_Ethekwini.pdf
2. National Department of Cooperative Governance & Traditional Affairs South Africa. King Cetshwayo District. 2020.
3. National Department of Cooperative Governance & Traditional Affairs South Africa. Ilembe District Municipality KZN [Internet]. 2020. p. 39. Available from: https://www.cogta.gov.za/ddm/wp-content/uploads/2020/11/Ilembe-September2020.pdf
4. National Department of Cooperative Governance & Traditional Affairs South Africa. Umkhanyakude District Municipality [Internet]. 2020 [cited 2001 Feb 20]. p. 1–24. Available from: https://www.cogta.gov.za/ddm/wp-content/uploads/2020/07/Umkhanyakude-DM-Final-JUNE-2020.pdf
5. National Department of Cooperative Governance & Traditional Affairs South Africa. Uthukela District Municipality EC [Internet]. 2000. p. 34. Available from: https://www.cogta.gov.za/ddm/wp-content/uploads/2020/11/Uthukela-October-2020.pdf
6. National Department of Cooperative Governance & Traditional Affairs. City of Cape town Metropolitan WC. 2020.
7. National Department of Cooperative Governance & Traditional Affairs South Africa. Buffalo City Metro EC [Internet]. 2000. p. 32. Available from: https://www.cogta.gov.za/ddm/wp-content/uploads/2020/08/DistrictProfile_BUFFALOCITY07072020-1.pdf
8. National Department of Cooperative Governance & Traditional Affairs South Africa. NMBM Metro EC [Internet]. 2000. p. 1–30. Available from: https://www.cogta.gov.za/ddm/wp-content/uploads/2020/11/Nelson-Mandela-Bay-Metro-October-2020-1.pdf
9. National Department of Cooperative Governance & Traditional Affairs South Africa. West Coast District Municipality WC [Internet]. 2000 [cited 2022 Jan 20]. Available from: https://www.cogta.gov.za/ddm/wp-content/uploads/2020/08/DistrictProfile_WESTCOAST23072020-002.pdf
10. National Department of Cooperative Governance & Traditional Affairs South Africa. Sarah Baartman District Municipality GAU [Internet]. 2000. p. 43. Available from: https://www.cogta.gov.za/ddm/wp-content/uploads/2020/08/DistrictProfile_SarahBaartman01072020.pdf

For peer review only

For peer review only - http://bmjopen.bmj.com/site/about/guidelines.xhtml

COREQ (COnsolidated criteria for REporting Qualitative research) Checklist

A checklist of items that should be included in reports of qualitative research. You must report the page number in your manuscript where you consider each of the items listed in this checklist. If you have not included this information, either revise your manuscript accordingly before submitting or note N/A.

Topic	Item No.	Guide Questions/Description	Reported on Page No.
**Domain 1: Research team and reflexivity**			
Personal characteristics			
Interviewer/facilitator	1	Which author/s conducted the interview or focus group?	
Credentials	2	What were the researcher's credentials? E.g. PhD, MD	
Occupation	3	What was their occupation at the time of the study?	
Gender	4	Was the researcher male or female?	
Experience and training	5	What experience or training did the researcher have?	
Relationship with participants			
Relationship established	6	Was a relationship established prior to study commencement?	
Participant knowledge of the interviewer	7	What did the participants know about the researcher? e.g. personal goals, reasons for doing the research	
Interviewer characteristics	8	What characteristics were reported about the inter viewer/facilitator? e.g. Bias, assumptions, reasons and interests in the research topic	
**Domain 2: Study design**			
Theoretical framework			
Methodological orientation and Theory	9	What methodological orientation was stated to underpin the study? e.g. grounded theory, discourse analysis, ethnography, phenomenology, content analysis	
Participant selection			
Sampling	10	How were participants selected? e.g. purposive, convenience, consecutive, snowball	
Method of approach	11	How were participants approached? e.g. face-to-face, telephone, mail, email	
Sample size	12	How many participants were in the study?	
Non-participation	13	How many people refused to participate or dropped out? Reasons?	
Setting			
Setting of data collection	14	Where was the data collected? e.g. home, clinic, workplace	
Presence of non-participants	15	Was anyone else present besides the participants and researchers?	
Description of sample	16	What are the important characteristics of the sample? e.g. demographic data, date	
Data collection			
Interview guide	17	Were questions, prompts, guides provided by the authors? Was it pilot tested?	
Repeat interviews	18	Were repeat inter views carried out? If yes, how many?	
Audio/visual recording	19	Did the research use audio or visual recording to collect the data?	
Field notes	20	Were field notes made during and/or after the inter view or focus group?	
Duration	21	What was the duration of the inter views or focus group?	
Data saturation	22	Was data saturation discussed?	
Transcripts returned	23	Were transcripts returned to participants for comment and/or	

For peer review only

Topic	Item No.	Guide Questions/Description	Reported on Page No.
		correction?	
**Domain 3: analysis and findings**			
Data analysis			
Number of data coders	24	How many data coders coded the data?	
Description of the coding tree	25	Did authors provide a description of the coding tree?	
Derivation of themes	26	Were themes identified in advance or derived from the data?	
Software	27	What software, if applicable, was used to manage the data?	
Participant checking	28	Did participants provide feedback on the findings?	
Reporting			
Quotations presented	29	Were participant quotations presented to illustrate the themes/findings? Was each quotation identified? e.g. participant number	
Data and findings consistent	30	Was there consistency between the data presented and the findings?	
Clarity of major themes	31	Were major themes clearly presented in the findings?	
Clarity of minor themes	32	Is there a description of diverse cases or discussion of minor themes?	

Developed from: Tong A, Sainsbury P, Craig J. Consolidated criteria for reporting qualitative research (COREQ): a 32-item checklist for interviews and focus groups. *International Journal for Quality in Health Care*. 2007. Volume 19, Number 6: pp. 349 – 357

Once you have completed this checklist, please save a copy and upload it as part of your submission. DO NOT include this checklist as part of the main manuscript document. It must be uploaded as a separate file.

For peer review only

The organisation of care for people receiving drug-resistant tuberculosis treatment in South Africa: a mixed methods study

Journal:	*BMJ Open*
Manuscript ID	bmjopen-2022-067121.R2
Article Type:	Original research
Date Submitted by the Author:	20-Sep-2023

Complete List of Authors:	Dickson, Lindy; University of Cape Town Faculty of Health Sciences, Department of Pathology<br>Le Roux, Sacha; University of Cape Town Faculty of Health Sciences, Department of Pathology<br>Mitrani, Leila; University of Cape Town<br>Hill, Jeremy; London School of Hygiene &Tropical Medicine , TB Centre<br>Jassat, Waasila; University of the Western Cape School of Public Health<br>Cox, Helen; University of Cape town, Medical Microbiology<br>Mlisana, Koleka; University of KwaZulu-Natal College of Health Sciences, School of Laboratory Medicine and Medical Sciences<br>Black, John; University of Cape Town Faculty of Health Sciences, Division of Infectious Diseases<br>Loveday, Marian; Medical Research Council of South Africa<br>Grant, Alison; London School of Hygiene & Tropical Medicine, TB Centre; Africa Health Research Institute<br>Kielmann, Karina; Institute of Tropical Medicine<br>Ndjeka, Norbert; National Department of Health; University of KwaZulu-Natal<br>Moshabela, Mosa; University of KwaZulu-Natal College of Health Sciences, School of Nursing and Public Health<br>Nicol, Mark; University of Cape Town; University of Western Australia

**Primary Subject Heading**:	Health services research
Secondary Subject Heading:	Infectious diseases, Patient-centred medicine, Health policy, Public health, Qualitative research
Keywords:	Tuberculosis < INFECTIOUS DISEASES, Health policy < HEALTH SERVICES ADMINISTRATION & MANAGEMENT, PUBLIC HEALTH, QUALITATIVE RESEARCH, Patient-Centered Care, Organisation of health services < HEALTH SERVICES ADMINISTRATION & MANAGEMENT

SCHOLARONE™
Manuscripts

I, the Submitting Author has the right to grant and does grant on behalf of all authors of the Work (as defined in the below author licence), an exclusive licence and/or a non-exclusive licence for contributions from authors who are: i) UK Crown employees; ii) where BMJ has agreed a CC-BY licence shall apply, and/or iii) in accordance with the terms applicable for US Federal Government officers or employees acting as part of their official duties; on a worldwide, perpetual, irrevocable, royalty-free basis to BMJ Publishing Group Ltd ("BMJ") its licensees and where the relevant Journal is co-owned by BMJ to the co-owners of the Journal, to publish the Work in this journal and any other BMJ products and to exploit all rights, as set out in our licence.

The Submitting Author accepts and understands that any supply made under these terms is made by BMJ to the Submitting Author unless you are acting as an employee on behalf of your employer or a postgraduate student of an affiliated institution which is paying any applicable article publishing charge ("APC") for Open Access articles. Where the Submitting Author wishes to make the Work available on an Open Access basis (and intends to pay the relevant APC), the terms of reuse of such Open Access shall be governed by a Creative Commons licence – details of these licences and which Creative Commons licence will apply to this Work are set out in our licence referred to above.

Other than as permitted in any relevant BMJ Author's Self Archiving Policies, I confirm this Work has not been accepted for publication elsewhere, is not being considered for publication elsewhere and does not duplicate material already published. I confirm all authors consent to publication of this Work and authorise the granting of this licence.

For peer review only

Title: The organisation of care for people receiving drug-resistant tuberculosis treatment in South Africa: a mixed methods study

Corresponding author: Mark Nicol

Email: mark.nicol@uwa.edu.au

Tel: (+61) 410 584 165

Address: School of Biomedical Sciences

University of Western Australia

M504, Perth, WA 6009, Australia

Author names and affiliations:

Lindy Dickson[1], Sacha Le Roux[1], Leila Mitrani[1], Jeremy Hill[3,4], Waasila Jassat[2], Helen Cox[1,5], Koleka Milsana[6], John Black[7,8], Marian Loveday[9,10], Alison D. Grant[11,12,], Karina Kielmann[13,14,], Norbert Ndjeka[15,16,], Mosa Moshabela [12,18], Mark P. Nicol[1,17]

1. Division of Medical Microbiology, University of Cape Town, Cape Town, South Africa
2. School of Public Health, University of the Western Cape, Cape Town, South Africa
3. Faculty of Medicine and Health, The University of Sydney, Sydney, New South Wales, Australia
4. Tuberculosis Research Program, Centenary Institute Medical Research Foundation, Newtown, New South Wales Australia
5. Institute for Infectious Disease and Molecular Medicine and Wellcome Centre for Infectious Disease Research in Africa, University of Cape Town, Cape Town, South Africa

For peer review only - http://bmjopen.bmj.com/site/about/guidelines.xhtml

6. School of Laboratory Medicine and Medical Sciences, University of KwaZulu-Natal, Durban, South Africa
7. Department of Infectious Diseases, Livingstone Hospital, Port Elizabeth, South Africa
8. Division of Infectious Diseases, Department of Medicine, University of Cape Town, Cape Town, South Africa
9. HIV and other Infectious Diseases Research Unit, South African Medical Research Council, South Africa
10. Centre for the AIDS Programme of Research in South Africa (CAPRISA), University of KwaZulu-Natal, Durban, South Africa
11. TB Centre, London School of Hygiene & Tropical Medicine, London, UK
12. Africa Health Research Institute, School of Laboratory Medicine and Medical Sciences, College of Health Sciences, University of KwaZulu-Natal, Durban, South Africa
13. Institute of International Health and Development, Queen Margaret University, Edinburgh, United Kingdom
14. Equity and Health Unit, Department of Public Health, Institute of Tropical Medicine Antwerp, Antwerp, Belgium
15. National Department of Health, Tuberculosis Control and Management Cluster, Pretoria, South Africa
16. Nelson R Mandela School of Medicine, University of KwaZulu-Natal, Durban, South Africa
17. Marshall Centre, Division of Infection and Immunity, School of Biomedical Sciences, University of Western Australia, Perth, Australia
18. School of Nursing and Public Health, University of KwaZulu-Natal, Durban, South Africa

For peer review only

Key Words: tuberculosis, drug-resistant, decentralisation, patient pathways, models of care, health care systems, person-centred care, South Africa

For peer review only

For peer review only - http://bmjopen.bmj.com/site/about/guidelines.xhtml

ABSTRACT

Objectives: Treatment for multidrug-resistant/rifampicin-resistant tuberculosis (MDR/RR-TB) is increasingly transitioning from hospital-centred to community-based care. A national policy for decentralised programmatic MDR/RR-TB care was adopted in South Africa in 2011. We explored variations in the implementation of care models in response to this change in policy, and the implications of these variations for people affected by MDR/RR-TB.

Design: A mixed methods study Patient movements between health care facilities were reconstructed from laboratory records, verified by folder reviews. Facility visits and staff interviews were used to determine reasons for movements.

Participants and Setting: People identified with MDR/RR-TB from 13 high-burden districts within South Africa

Outcome measures: Geospatial movement patterns were used to identify organisational models. Reasons for patient movement and implications of different organisational models for people affected by MDR/RR-TB and the health system were determined.

Results: Among 191 participants, six dominant geospatial movement patterns were identified, which varied in average hospital stay (0-281 days), average patient distance travelled (12-198km) and number of health facilities involved in care (1–5 facilities). More centralised models were associated with longer delays to treatment initiation and lengthy hospitalisation. Decentralised models facilitated family-centered care and were associated with reduced time to treatment and hospitalization duration. Responsiveness to the needs of people affected by MDR/RR-TB and health system constraints was achieved through implementation of flexible models, or the implementation of multiple models in a district.

For peer review only - http://bmjopen.bmj.com/site/about/guidelines.xhtml

Conclusions: Understanding how models for organising care have evolved may assist policy implementers to tailor implementation to promote particular patterns of care organisation or encourage flexibility, based on patient needs and local health system resources. Our approach can contribute towards the development of a health systems typology for understanding how policy-driven models of service delivery are implemented in the context of variable resources.

For peer review only

Strengths and limitations of this study

- We used real-world patient movement pathways to understand emerging models of care in response to a change in policy.
- We triangulated several different sources of data, including laboratory data, interviews with health care providers and facility visits to understand the implications of these emerging models of care for people affected by MDR/RR-TB and the health system.
- We could not clearly differentiate to what degree patient needs versus health system organisation and resources determined individual care pathways.
- We were not able to adequately assess care quality indicators, and so are unable to determine whether particular organisational models were associated with favourable patient outcomes.
- We did not interview people affected by MDR/RR-TB, and so cannot draw conclusions about their preferences.

For peer review only

BACKGROUND

Tuberculosis (TB) has been the leading cause of death in South Africa for two decades (1) and claims the lives of around 330 persons daily worldwide(2). Multidrug-resistant (MDR-) TB is defined as tuberculosis caused by *Mycobacterium tuberculosis* that is resistant to at least the two key drugs, rifampicin and isoniazid, whilst rifampicin-resistant (RR-) TB is defined by resistance to rifampicin with or without isoniazid resistance. MDR/RR-TB caused illness in around 14 000 persons in South Africa in 2019(3), is more complex and costly to treat than drug sensitive TB(4), and is associated with a fourfold higher mortality (1,5). Within the next 20 years, the MDR/RR-TB case load in South Africa is projected to increase substantially(6,7). Persons with MDR/RR-TB often experience complex medical, psychological(8), social(9,10), and economic(11) challenges. Management for people with MDR/RR-TB requires an intensive multidisciplinary, comprehensive approach, and typically consumes a disproportionate share of health resources(11). Treatment provision requires access to specialised drugs and skilled staff to monitor people with MDR/RR-TB over many months(11). The sustainable use of newer regimens to address this case load will require strengthening MDR/RR-TB treatment delivery systems to ensure effective, person-centred care(12). The WHO End TB Strategy advises a country-specific, tailored, and strategic approach to achieving universal access to high-quality treatment for MDR/RR-TB(13,14).

In response to the rising MDR/RR-TB caseload in South Africa and to local and international pressure from patient and advocacy groups, the National Department of Health (NDoH) adopted an approach to increase ambulatory management of MDR/RR-TB in 2011 (15). This strategy, which had not been widely implemented elsewhere, but was in line with pilot projects

For peer review only - http://bmjopen.bmj.com/site/about/guidelines.xhtml

in South Africa, Peru and Lesotho, was intended to improve treatment access and reduce in-hospital stays(16,17). Prior to decentralisation, people with MDR/RR-TB in South Africa were primarily managed within facilities specialising in MDR/RR-TB care, frequently with long hospital stays, before being discharged under the ongoing care of the specialised facilities. The decentralisation strategy aimed to increase the number of treatment sites from one or two dedicated MDR/RR-TB facilities per province to several facilities capable of providing MDR/RR-TB care within each district(18). A national guideline for decentralisation of care was published in 2011(19) and updated in 2019(20), and served as a generic, non-prescriptive tool for health managers. Since then, implementation of the policy to decentralise MDR/RR-TB care within South Africa has varied widely (21), in response to differences in health system capacity and readiness to cope with change(22)(23)(22). Funding to support decentralisation was not specifically allocated, which resulted in further setting-specific innovations and adaptations to the program at both provincial and district level(24,25).

We have previously described variation in the care journeys of people with MDR/RR-TB(21), which may reflect differences in patient co-morbidity or disease features, but likely also reflects regional differences in the organisation of care for the management of MDR/RR-TB. Ideally, models for organisation of care should attempt to improve patient outcomes through coordinated care that is person-centred, timely and effective (26); however, models of care often evolve to suit health care providers and health systems convenience (27–29).

We therefore studied variation in the patterns of organisation of care for people with MDR/RR-TB which have emerged in South Africa because of local variation in guideline implementation.

For peer review only - http://bmjopen.bmj.com/site/about/guidelines.xhtml

We used patient care pathways to identify patterns of care provision, outline the implications of these patterns for the needs of people with MDR/RR-TB and draw lessons on different ways in which care for patients with complex illness can be organised.

METHODS

The overarching research project, within which this sub-study was nested, was led by the same group of investigators, funded by a Health Systems Research Initiative award from the Medical Research Council of the United Kingdom and aimed to identify interventions to optimise decentralisation of services for people with MDR/RR-TB (Supplementary Figure 1). Ethical approval for the study was provided by the University of Cape Town, South Africa (350/2016), and the London School of Hygiene & Tropical Medicine, UK (11680). The project used a stepwise realist approach to understand the policy context, implementation and working models of decentralisation of MDR/RR-TB care in South Africa. The aim of this sub-study, which was conducted over the same period, was to explore variations in the implementation of care models in response to a change in MDR/RR-TB health policy, and the implications of these variations for people with MDR/RR-TB and the health services.

Study design and setting

We used a multiple case study methodology (30). Data collection included a retrospective cohort study of geographically-linked laboratory records, validated by patient folder reviews. All TB samples in the state sector in South Africa are sent to laboratories which form part of the National Health Laboratory Services (NHLS) for testing. The NHLS has a centralised data

For peer review only - http://bmjopen.bmj.com/site/about/guidelines.xhtml

repository which records all samples sent to laboratories within the network, together with results and patient details. Patterns of patient movement and facility visits were mapped. Semi-structured interviews with health care workers were undertaken to verify patient journey patterns and organisational patterns, and to determine their implications for the health service and people with MDR/RR-TB. We defined MDR/RR-TB as resistance to rifampicin (with or without isoniazid resistance), detected using genotypic or phenotypic methods, irrespective of resistance to other anti-TB drugs.

Three provinces in South Africa (KwaZulu-Natal [KZN], Eastern Cape [EC] and Western Cape [WC]) were selected due to their high MDR/RR-TB disease burden and differing approaches to decentralised care (Supplementary Figure 2). Within each province, two urban and two rural districts were selected based on observed differences in patient pathways from the parent study(31). One additional rural district was selected in KZN, due to a rapid shift in urbanisation associated with relocation of the province’s airport.

The study took place between July 2016 and July 2019. During this period, MDR/RR-TB treatment regimens were largely based on a standardised aminoglycoside-containing regimen (requiring an injectable drug), tailored according to individualised drug-susceptibility patterns. Prior to the study period, rapid molecular testing for TB and MDR/RR-TB (Xpert MTB/RIF) had been implemented, while limited roll-out of the novel anti-tuberculous drug bedaquiline in pilot programmes and the introduction of a shortened MDR/RR-TB treatment regimen occurred over the study period. People with MDR/RR-TB generally underwent scheduled monthly

For peer review only

clinical evaluation and investigation over the treatment duration, in line with World Health Organisation (WHO) guidelines(31).

Selection of people with MDR/RR-TB and assessment of patient movement pathways

We have previously described the methods used to construct MDR/RR-TB patient healthcare journeys(31). Briefly, National Health Laboratory Service laboratory records were used to generate a list of all people in the study districts with new bacteriologically-confirmed MDR/RR-TB from July to September 2016 (n=2 649). A random sample of 195 people with MDR/RR-TB was selected from this list, stratified by district. One patient was found to have been incorrectly diagnosed with MDR/RR-TB, leaving 194 in the analysis. The laboratory records for the 194 people served as tracers of their pathways over a 9-month period(31). Geographic coordinates of healthcare facilities were collected, and spatial-temporal mapping of patient movement was used to reconstruct patient movement pathways in chronological order for each sampled person with MDR/RR-TB, including movement outside the study districts.

Data on treatment progress were collected from clinical record reviews, telephonic enquiries with clinics and the electronic MDR/RR-TB registry [EDRWeb] up to two years after diagnosis. Data on patient movement obtained from laboratory records were confirmed by telephonic enquiries at all health care facilities attended by people with MDR/RR-TB. This input was used to verify laboratory information, determine additional clinic visits (where no laboratory sample was taken), obtain demographic and health data and to identify reasons for movement of sampled people with MDR/RR-TB between facilities. Descriptive statistics for the sampled

For peer review only - http://bmjopen.bmj.com/site/about/guidelines.xhtml

districts and participants were extracted. Time from laboratory diagnosis to treatment initiation and time on treatment were calculated (32).

All movement between health care facilities for each study participant was geospatially mapped, based on 'as the crow flies' measurements(33). Patient pathway patterns sharing similar appearance were then grouped according to their pattern and named, as described in other studies(34)(35) (36). Pattern descriptions included the level of facilities visited (ranging from quaternary care level to mobile clinic), movements between facilities, facility admission period or 'period under care' (where facilities retained responsibility for monthly follow up of people with MDR/RR-TB after discharge) and sector of care (state or private). The number of visits (or patient-days) spent under care of a centralised service were used to position the patterns on a decentralisation continuum.

Detailed assessment of organisation of services for MDR/RR-TB

A total of 22 facilities representing the spectrum of patterns were chosen for more detailed investigation of organisation of MDR/RR-TB care. These facilities represented six urban and seven rural districts as well as the different levels of care ranging from primary health care to tertiary care. At each facility we conducted patient folder reviews and semi-structured interviews with health workers, including pharmacists (n=2), out-patient physicians (n=2), inpatient physicians (n=6), TB facility managers (n=5), ward nurses (n=9), out-patient nurses (n=8) [including HIV care nurses (n=2), paediatric care nurses (n=2)], and lay health care workers (n=2). Health care workers were selected for interview based on their involvement with the MDR/RR-TB care programme at their facility. No participants refused to participate or dropped out and only the researchers and participant/s were present during interviews. For

For peer review only - http://bmjopen.bmj.com/site/about/guidelines.xhtml

interviews, specific patient trajectories related to each facility were used as prompts for discussion of the reasons for and implications of patient movements between facilities. Additional data on the same themes were extracted from patient folder reviews. The interviews were conducted in English, with some translation from Afrikaans, and recorded, except in a prison where recording was not permitted. Most interviews were conducted with individuals or pairs of informants, with two interviews involving multidisciplinary panels at a facility. Transcripts of interviews and folder review data were reviewed by seven researchers who extracted information relating to the implications of the different patterns for care organisation for both people with MDR/RR-TB and the health services. A COREQ checklist is included (Supplementary Material). To further identify the implications of the different organisational patterns for care outcomes and person-centredness for people with MDR/RR-TB and the health services, we used key metrics including time to treatment, length of hospital stay, number of facilities visited, and distance travelled.

Patient and Public Involvement Statement

This study involved neither patients nor members of the public. Findings from this study were disseminated during national MDR/RR-TB meetings organised by the Department of Health and non-governmental organisation partners and presented to programme implementers from all nine provinces of the country.

RESULTS

Cohort description

For peer review only - http://bmjopen.bmj.com/site/about/guidelines.xhtml

We studied urban and rural districts from three high TB burden provinces (Table S1). A total of 191 participants had sufficient data available to map treatment pathways and outcomes (the remaining 3 participants had too few data points to assess)(37). Of the 191 people with MDR/RR-TB, 63% (n=120) were male and 92% (n=177) were aged 20-59 years. Approximately half of the participants lived in an urban area (46%, n=88). Sixty-three percent (n=121) had previously been treated for TB, with a further 19% (n=36) previously treated for MDR/RR-TB. 69% (n=131) were coinfected with HIV at the time of MDR/RR-TB diagnosis (37). Two thirds (65%, n=124) were unemployed at the time of diagnosis. Fifteen percent (n=28) had a history of correctional services detention. Approximately one quarter (24%, n=46) reported comorbidities other than TB and HIV at diagnosis. Five percent (n=10) had a household member die from TB disease. All participants were treated exclusively in the state health sector, except for two participants who also received treatment in the private sector. We have previously described in detail the clinical comorbidities and social needs of the people with MDR/RR-TB in this cohort(37).

Sixty-four percent (n=122) of the cohort were diagnosed with MDR/RR-TB; 16% (n=31) with pre-extensively drug-resistant (pre-XDR), 10% (n=19) with XDR-TB and 10% (n=19) with MDR/RR-resistance with unknown second-line drug resistance. Of the 191 participants 80% (n=153) were alive at 6 months, and 77% (n=148) were alive at 2 years after laboratory diagnosis. At 24 months after laboratory diagnosis, 46% of participants (n=88/191) had either completed treatment or achieved 'cure', 19% (n=37) had ongoing treatment, 10% (n=19) were lost to follow-up, 2% (n=3) had unknown outcome and 0.5% (n=1) had ongoing illness where TB treatment was deemed futile, and the patient had been referred for palliative care.

For peer review only - http://bmjopen.bmj.com/site/about/guidelines.xhtml

Geospatial patterns of patient movement and their distribution

A total of 191 patient movement pathways could be classified into six dominant geospatial movement patterns (Table 1 and Figure 1). These were named according to the trajectories of patient movements as A ‘fan’ [n=41 (22%)], B ‘chicken-foot’ [n=19 (10%)], C ‘hub and spoke’ [n=72 (38%)], D ‘triangle’ [n=30 (15%)], E ‘line’ [n=12 (6%)], and F ‘dot’ [n=17 (9%)]. The patterns were placed on a continuum from the most centralised, ‘fan’, to the most decentralised, ‘dot’. The ‘hub and spoke’ configuration was the most frequently observed pattern across all three provinces in both urban [n=35 (18%)] and rural [37 (19%)] settings (Table 2). Several typical patient pathways for each pattern are depicted on a stylised study district in Figure 1.

Description of organisation of care within each of the patterns

We used facility visits, staff interviews and patient record review to describe the organisation of care within each of these patterns. The most centralised pattern, ‘fan’, is highly dependent on the designated MDR-TB Centre of Excellence (CoE) to manage people with MDR/RR-TB, following initial assessment at a peripheral facility. CoEs were historically designated by the provincial health departments to manage people with complex TB. The first step in increasing decentralisation of MDR/RR-TB services was the establishment of specialised MDR-TB hospitals, as a ‘step down’ from the CoEs. The ‘chicken-foot’ pattern demonstrates some decentralisation to these specialised hospitals for routine cases, but with dependence on the CoEs for caring for people with more extensive TB drug resistance or complex presentations. This pattern was common for regions with higher XDR-TB or MDR/RR-TB numbers and where new drugs were being introduced into TB regimens. Although the participant numbers in the

For peer review only

For peer review only - http://bmjopen.bmj.com/site/about/guidelines.xhtml

study limited outcome analysis, observation suggests that this model has the greatest risk for people with MDR/RR-TB for retention in care, treatment outcome and prolonged hospital stay (Table 1). The' hub and spoke' pattern, which was predominately observed in rural districts, primarily utilises specialised MDR-TB hospitals for all care provision, with little capacity existing at primary care level. These three models are considered the more centralised of the six models identified.

The 'triangle' pattern incorporates a more flexible network of MDR-TB services and indicates availability of MDR-TB services at many facilities within the district. Typically, this pattern has oversight by a specialist infectious diseases or TB physician, who is either within the district or closely monitors patient progress through telemedicine. This 'conductor' is responsible for weighing complex social and medical needs and for directing patient movement to an appropriate level of care, according to the clinical need and social circumstances of the patient, at various points during their care. This organisational model is also able to respond to changes in the local availability of MDR-TB resources, by modifying patient referral pathways. This model is frequently associated with decision-making shared between people with MDR/RR-TB and clinicians.

The 'line' and 'dot' patterns reflect a substantial shift to outpatient clinic-based care, frequently including MDR-TB clinical practitioners providing mobile outreach services to different subdistrict primary health care sites. In the line pattern, not all clinics had capacity to initiate treatment, which resulted in some clinics referring to neighbouring clinics, or a district hospital for outpatient treatment initiation or a short admission for early management and stabilisation. In the 'dot' pattern, clinics had capacity to initiate and maintain MDR/RR-TB treatment

For peer review only

independently, with pharmacy and multidisciplinary resources available. This 'dot' model was associated with the best treatment outcomes, however the sample size was small and possible health care worker selection of participants with less severe illness (to cope with a fully decentralised care option) limits generalisability of these findings.

Different patient movement pathways may reflect organisational models within the health system, the incidence of MDR/RR-TB within districts as well as patient features which require delivery of care at a particular level(38). We observed that the distribution of the different movement patterns across districts differed. Most districts had three or four patterns identified (Table 2). This suggests flexibility in the organisation of care within these districts to respond to differing patient or health system needs.

We conducted exploratory analysis to assess whether patient clinical features were associated with certain patterns of care (Table 3). Children, participants older than 60 years and people with XDR -TB/ pre-XDR-TB seldom followed the more decentralised 'line' and 'dot' patterns. There was otherwise no evidence that patterns of care differed by patient HIV status, previous TB treatment, low body mass index, or higher number of co-morbidities; however, the small sample size precludes robust statistical analysis.

Implications of different patterns of organising care for people with MDR/RR-TB and the health system

We observed care pathways, reviewed patient records, visited healthcare facilities, and interviewed facility staff to identify the implications of different organisational patterns on

For peer review only - http://bmjopen.bmj.com/site/about/guidelines.xhtml

people with MDR/RR-TB and the health system (Table 4). More decentralised models ('line' and 'dot') had advantages such as quick linkage to care, shorter hospital stays, and less travel distance for diagnosis and treatment. However, these models of care were compromised at times through the risk of clinical practitioner shortages and less rigorous treatment monitoring. More centralised models ('fan' and 'chicken foot') had advantages such as multidisciplinary clinical, pharmacy, radiological, and laboratory resources in one facility, but had disadvantages including long travel distances, treatment initiation delays, and patient travel distance. (Table 1).

DISCUSSION

This study used a novel approach, combining quantitative and qualitative methods and patient pathway analysis, to describe emerging patterns of decentralised care for MDR/RR-TB in South Africa. We identified six patterns of care organisation, ranging from highly centralised to highly decentralised models, with several different models operational in many study districts. The different models demonstrated distinct advantages and disadvantages for both people with MDR/RR-TB and the health system. Although exploratory, the patterns identified can contribute to developing a health systems typology for understanding how policy-driven models of service delivery are implemented in the context of variable resources and differing patient needs. Explicit recognition of the divergence of organisational models and cross-district learning around different models for organising care may provide opportunity for health system managers to optimise delivery of care in different contexts, and should be considered by policy developers when planning interventions to decentralise care for complex illness.

For peer review only - http://bmjopen.bmj.com/site/about/guidelines.xhtml

384 Stokes et al have previously described a framework for analysing models of care for patients
385 with multimorbidity(32). A key component of this framework is the organisation of care across
386 different levels of the health system. We and others have previously described other elements
387 of this framework in relation to MDR/RR-TB in South Africa, including the theoretical basis for
388 the decentralisation policy(39), the clinical and social needs of the target population(37,40),
389 and the local adaptations of the decentralisation policy(22,41,42). Our approach to identifying
390 organisational models, which involved analysis of patient movement pathways, may be a
391 useful tool and contribute valuable insights into understanding variation in policy
392 implementation. This approach also provides important metrics, including the number of
393 different facilities visited, and travel distances. These metrics differed substantially between
394 models in our study, with more centralised models associated with prolonged hospitalisation
395 and multiple transfers between facilities. Although people with MDR/RR-TB who are treated
396 with adequate doses of appropriate drugs rapidly become non-infectious(43), there is
397 increasing recognition that multiple patient transfers (often together with non-TB patients) and
398 prolonged hospitalisation may expose both people with MDR/RR-TB and others to
399 unnecessary infectious risks, reduce retention in care and incur financial costs to people with
400 MDR/RR-TB (44,45). Studies in LMICs have shown that physical proximity of healthcare
401 services can play an important role in the use of healthcare facilities(46–48).

402

403 People with MDR/RR-TB in this cohort presented with multiple medical comorbidities,
404 including mental illness, and challenging social circumstances(37). Our exploration of the
405 advantages and disadvantages of the different organisational patterns highlights the trade-
406 offs when having to consider people with complex or severe disease. Since it is not possible
407 to decentralise all components of care for people with complex MDR/RR-TB, flexibility to

For peer review only - http://bmjopen.bmj.com/site/about/guidelines.xhtml

provide differentiated or individualised care, which adapts to address the specific requirements of individuals or a subgroup of people with MDR/RR-TB, is particularly relevant (49) (20,22,50,51). For example, a flexible referral model like 'triangle', which utilises centralised clinical oversight to direct patient care, or implementation of multiple healthcare models (as observed in the Western Cape) may allow for a more seamless patient flow in response to the needs of people with MDR/RR-TB. Such flexible healthcare models, that can be organised in response to operational constraints and patient needs, may lead to contextually sensitive and optimal patient care that is safe, effective, person-centred, timely, efficient, and equitable (52–54). Although we did not collect data on patient costs, since centralised models were associated with longer hospital stays and travel distances, as well as fewer participants receiving an income, it is likely that patient costs were higher with centralised models. Fewer participants not receiving income fall into the line and dot patterns, which could point to these people with MDR/RR-TB being able to keep on with their employment and livelihoods. The elimination of catastrophic costs to families affected by TB is one of the key pillars of the End-TB strategy (55).

Care integration, and models that aim to overcome fragmentation between providers have been identified as important(56). Ideally, a bio-psychosocial systems approach should be used, where the primary focus of health care is the client in the context of their family(57) and where adult and paediatric services are provided together in a single setting(58). Reviews of family-centred care models in HIV care, indicate excellent adherence, retention in care, and low mortality and/or loss to follow-up in both adult and child services(59). Since HIV care in South Africa is highly decentralised, HIV status did not appear to affect the degree of decentralisation for people with MDR/RR-TB, even for people living with HIV who had low CD4

For peer review only - http://bmjopen.bmj.com/site/about/guidelines.xhtml

values. The dot model, where services are provided closest to the community is likely to be most family centred, but may be less ideal where home social circumstances are difficult, or where people with MDR/RR-TB require highly specialised care (60). In the latter circumstances, models, such as triangle, which are intrinsically more flexible, and responsive to changing patient needs, whilst containing a mix of care expertise, levels and disciplines may be more appropriate.

Societal features, such as urbanization, may have driven the emergence of certain models. However, we found no clear association between care patterns and epidemiological features of districts. For example, the most decentralised patterns (line and dot) were seen both in highly urbanised districts with large numbers of cases as well as in sparsely populated rural areas. These models appeared to be responses to differing pressures – the need to commence large numbers of people with MDR/RR-TB on treatment in urban areas, and the need to deliver care closer to people with MDR/RR-TB in remote areas. In rural districts in the Eastern Cape and KZN, where several of the more decentralised models were available, these appeared to be driven by clinician champions, as we have previously described(61). In the Western Cape, the pre-existing strength of the primary health care system may have facilitated the preponderance of line and dot models.

The WHO annual TB report card on South Africa cautions that reported numbers of people diagnosed and commenced on TB treatment in 2020 had fallen by 25% compared to the same period in 2019 and that gains in the TB program made over the past decade could be reversed by the COVID-19 pandemic(62). Countries have been encouraged to mitigate COVID-19

For peer review only

For peer review only - http://bmjopen.bmj.com/site/about/guidelines.xhtml

related impacts on MDR/RR-TB services by expanding use of digital technologies for remote advice and support, improving community connectivity(63), reducing the need for visits to health care facilities, encouraging home-based treatment and increasing use of strategies for facilitating patient drug supply. Lessons from the COVID-19 pandemic may offer lessons for organisation of MDR/RR-TB care delivery, in particular consideration of opportunities to improve connectivity to enhance decentralised care. We identified several examples where either telemedicine had been used, in line or dot models, or physicians travelled to enable peripheral facilities to initiate and care for newly-diagnosed or more people with complex illness. In some instances, medication prescriptions or clinical records travelled in place of people with MDR/RR-TB. However, these adaptations were not a formal system response but rather an example of local adaptation, often with staff using their own professional networks, personal devices and internet connectivity for communication(22). Description of patient movement patterns, critical appraisal of reasons for patient movement, and identification of which travel might be avoided through other equally effective means, such as virtual consultations, may be a useful exercise for diseases that are complex, rare or require specialised care (64–66).

Our study was undertaken prior to the widespread implementation of 6-month, all oral regimens for MDR/RR-TB (bedaquiline, pretomanid, linezolid and moxifloxacin [BPaLM]), which may affect decentralisation models further. It is possible that these relatively simpler regimens may facilitate further decentralisation. However, this is countered by the need for ECG monitoring for cardiotoxicity, together with the need to provide ongoing training and mentorship to the staff involved. We observed that drug stockouts were a problem at

For peer review only - http://bmjopen.bmj.com/site/about/guidelines.xhtml

decentralised sites – this may be a particular issue for more costly new drugs, due to concerns around shelf-life and wastage.

A limitation of our study is that it is not possible to clearly differentiate to what degree patient needs versus health system organisation and resources determined individual care pathways, but both are likely to have played a role. The substantial differences in care organisation, regardless of patient needs, reflect that there were multiple factors that were likely to determine care organisation. The sample size limited our ability to adequately assess care quality indicators, and so are unable to determine whether particular organisational models were associated with favourable patient outcomes. Further, we did not interview people with MDR/RR-TB, and so cannot draw conclusions about patient preferences. People with MDR/RR-TB who did not link to care, who died shortly after linking to care, and those linking through the private sector were not included in this study. Since the entry point to the study was a microbiological diagnosis, we are unable to quantify the effect of decentralised management on diagnostic delay. We were unable to determine changes to the degree of decentralisation and evolution of movement patterns during the study period.

In this analysis, we also did not specifically interrogate the reasons for the emergence of the different organisational models. Models of care are often based on what was in place historically, and over time become less responsive to the changing needs of the person with MDR/RR-TB and to health system constraints(67). Models of care are also often developed to bridge service delivery gaps rather than as a planned strategic response to an identified local

For peer review only - http://bmjopen.bmj.com/site/about/guidelines.xhtml

need (68,69). The development of models of care is commonly an iterative process(70,71), shaped by socio-political, economic, cultural, environmental, and legal drivers (72,73).

CONCLUSION

With the challenge of striving towards the WHO targets of zero deaths, disease and suffering due to TB by 2035 (74), stronger and more flexible health systems for decentralising patient management of MDR/RR-TB will be required to accommodate varying treatment needs. Indeed, a key finding of our study is that flexible models for offering decentralised care have emerged in different settings, in response to differing patient needs. The study of patient care pathways is a useful approach to providing a health systems typology of the ways in which care is organised for complex conditions. Having more clearly defined and articulated models of care may assist policy planners and implementers to identify which organisational models are most responsive to patient needs, quality indicators and system constraints. Increased use of digital technologies should be explored to democratise specialised and individualised care for MDR/RR-TB and other complex illnesses.

For peer review only - http://bmjopen.bmj.com/site/about/guidelines.xhtml

DECLARATIONS

Acknowledgements

The authors wish to thank the provinces of the W Cape, Eastern Cape, KwaZulu-Natal, and acknowledge the staff at the NHLS for their tremendous input and assistance. We give special mention to the late Dr Iqbal Masters and Mrs Anna Maria Evans for their contributions to the study. We also appreciate the support of Staff Nurse Cheryl Liedeman and Dr Widaad Zemanay.

Competing interests:

The authors declare that they have no competing interests.

Funding

This study was supported by a Health Systems Research Initiative award from the Medical Research Council of the United Kingdom and the Wellcome Trust (MR/N015924/1). This UK funded award is part of the EDCTP2 program supported by the European Union. HC is supported by a Wellcome Trust Fellowship. MN is supported by an Australian National Health and Medical Research Council Award (APP1174455). No conflicts of interest were reported.

Authors' contributions

Conception and design of study: MN, HC, MM, AG, LD and KK

Planning of the work: MN, HC, MM, AG, LD, KK, KM, JB, ML, NN, WJ

Interrogation and analysis of laboratory reports and geo-mapping: JH, LD, SLR, LM, MN, HC

For peer review only - http://bmjopen.bmj.com/site/about/guidelines.xhtml

Field visits and interviews: LD, SLR, WJ, JH, LM

Analysis of field worker and interview data: LD, SLR, WJ, JH, LM, KK, HC, MN

Conceptualizing manuscript: LD, MN

Drafting manuscript and revisions: LD, MN

Reviewing manuscript and approval of final version: MN, HC, MM, AG, LD, KK, KM, JB, ML, NN, JH, SLR, LM, WJ

For peer review only

For peer review only - http://bmjopen.bmj.com/site/about/guidelines.xhtml

Ethics approval and consent to participate

The study was a retrospective laboratory and record review of routinely captured data and a waiver of informed consent was granted to retrospectively review these records. Participants did not provide consent to participate in the study as all data analysed were collected as part of routine diagnosis and care. This study was approved by ethics boards at the University of Cape Town (350/2016) and the London School of Hygiene & Tropical Medicine (11680). Research approval was obtained in each province (Eastern Cape 2016RP30-232, KwaZulu-Natal 2016RP51-466, Western Cape 2016RP45-978).

Consent for publication

Not applicable

Data sharing:

The dataset (anonymised participant data) used and analysed during the current study are not publicly available but can be obtained from the corresponding author (ORCID identifier 0000-0002-0020-1414) on reasonable request through a controlled access repository.

Word count: 4670

For peer review only - http://bmjopen.bmj.com/site/about/guidelines.xhtml

Figure titles and legends:

Figure 1. Examples of the dominant geospatial movement patterns annotated on a stylised study district. Each coloured line depicts a single participant travelling in a pathway between the specific levels of health care facilities found in that model. Arrows represent direction of participant movement direction between health care facilities. Stars represent participants who only visited a single facility across their diagnostic and treatment pathway. Green crosses represent Primary Health Care Clinics, Red Crosses represent General Hospitals, Blue H represents Specialized TB Hospital and Orange H represents Centre of Excellence TB Hospital. A: ’Fan’; B: ‘Chicken Foot’; C;’ Hub and Spoke’; D: ‘Triangle’; E: ‘Line’; F: ’Dot’.

References

1. Loveday M, Mzobe YN, Pillay Y, Barron P. Figures of the dead: A decade of tuberculosis mortality registrations in South Africa. S Afr Med J. 2019;109(10):728–32.

2. Statistics South Africa. Mortality and causes of death in South Africa, 2016: Findings from death notification. Statistical release. 2016;(November 2016):1–127.

3. World Health Organization; Global tuberculosis report 2020 [Internet]. Geneva; 2020 [cited 2020 Jun 1]. Available from: https://www.who.int/teams/global-tuberculosis-programme/tb-reports/global-tuberculosis-report-2020

4. Murdoch J, Curran R, Rensburg AJ Van, Awotiwon A, Dube A, Bachmann M, et al. Identifying contextual determinants of problems in tuberculosis care provision in South Africa : a theory - generating case study. Infect Dis Poverty. 2021;1–13.

5. Sineke T, Evans D, Schnippel K, Van Aswegen H, Berhanu R, Musakwa N, et al. The impact of adverse events on health-related quality of life among patients receiving treatment for drug-resistant tuberculosis in Johannesburg, South Africa. Health Qual Life Outcomes. 2019;17(1).

6. World Health Organization. Consolidated Guidelines on Tuberculosis Treatment. World Health Organization; 2016. 99 p.

7. World Health Organization. Companion handbook. World Health Organization; 2014. 464 p.

8. IOM (Institute of Medicine). Patient Safety: Achieving a NewStandard for Care. Vol. 12, The National Academies Press. Washinton,DC; 2004. 126–129 p.

For peer review only

For peer review only - http://bmjopen.bmj.com/site/about/guidelines.xhtml

Law S, Daftary A, O'Donnell M, Padayatchi N, Calzavara L, Menzies D. Interventions to improve retention-in-care and treatment adherence among patients with drug-resistant tuberculosis: A systematic review. European Respiratory Journal. 2019;53(1):13993003.

Mohr E, Hughes J, Snyman L, Beko B, Harmans X, Caldwell J, et al. Healthcare delivery: Patient support interventions to improve adherence to drug-resistant tuberculosis treatment: A counselling toolkit. South African Medical Journal. 2015;105(8):631–4.

Hochman M, Asch SM. Disruptive Models in Primary Care: Caring for High-Needs, High-Cost Populations. J Gen Intern Med. 2017;32(4):392–7.

ReAct Group. Learning from Experiences : Introducing Bedaquiline in South Africa Executive summary [Internet]. 2020 [cited 2021 Nov 18]. Available from: https://www.reactgroup.org/news-and-views/news-and-opinions/year-2020/learning-from-bedaquiline-in-south-africa-comprehensive-health-systems-for-new-antibiotics/

Gray RM. The paradigms global plan of TB. Archetypal Explorations. 2020;108–28.

Stop TB Partnership. The Global Plan to End TB 2016-2020: The Paradigm Shift. World Health Organization Document. 2015;124.

Overland L, Uidelines C. Understanding the process to develop a Model of Care An ACI Framework. Perspectives on Swallowing and Swallowing Disorders (Dysphagia). 2009;20(3):60–4.

Loveday M, Wallengren K, Voce A, Margot B, Reddy T, Master I et al. Comparing early treatment outcomes of MDR-TB in decentralised and centralised settings in KwaZulu-Natal, South Africa. Int J Tuberc Lung Dis. 2012;16(2):209–15.

Padayatchi N, Friedland G. Decentralised management of drug-resistant tuberculosis (MDR- and XDR-TB) in South Africa: An alternative model of care. International Journal of Tuberculosis and Lung Disease. 2008;12(8):978–80.

World Health Organization. Guidelines for the programmatic management of drug-resistant tuberculosis Guidelines for the programmatic management of drug-resistant tuberculosis. World Health Organization. 2008;12–185.

National Department of Health South Africa. Multi-drug resistant tuberculosis: A policy framework on decentralised and deinstitutionalised management for South Africa. Pretoria; 2011.

National Department of Health South Africa. Multi-drug resistant tuberculosis a policy framework on decentralised and deinstitutionalised management for south africa. 2019.

Cox, HS; Dickson-Hall, L; Jassat W et al. Drug-resistant tuberculosis in South Africa: history, progress and opportunities for achieving universal access to diagnosis and effective treatment. In: South African Health Review 2017. 2017. p. 157–67.

Kielmann K, Dickson-Hall L, Jassat W et al. “We had to manage what we had on hand, in whatever way we could”: Adaptive responses in policy for decentralized drug-resistant tuberculosis care in South Africa. Health Policy Plan. 2021;36(3):249–59.

Kielmann K, Dickson-Hall L, Jassat W, Le Roux S, Moshabela M, Cox H, et al. “We had to manage what we had on hand, in whatever way we could”: Adaptive responses in policy for decentralized drug-resistant tuberculosis care in South Africa. Health Policy Plan. 2021;36(3):249–59.

For peer review only

For peer review only - http://bmjopen.bmj.com/site/about/guidelines.xhtml

WHO. Implementing The End TB Strategy. Antimicrob Agents Chemother. 2015;58(12):7250–7.

The Global Fund. Best Practices on TB Case Finding and Treatment. The global fund org. 2018;73.

Cott CA, Davis AM, Badley EM, Wong R, Canizares M, Li LC, et al. Commonalities and differences in the implementation of models of care for arthritis: Key informant interviews from Canada. BMC Health Serv Res. 2016;16(1):1–12.

World Health Organization; Everybody's business strengthening health systems to improve health outcomes. Geneva; 2007.

Saha, S, MD, Beach, MC, Cooper L. Patient Centeredness, Cultural Competence and Healthcare Quality. 2010;100(11):1275–85.

National Department of Health South Africa. White paper for the transformation of the health system in South Africa. 1997.

Walt G, Shiffman J, Schneider H, Murray SF, Brugha R, Gilson L. "Doing" health policy analysis: Methodological and conceptual reflections and challenges. Health Policy Plan. 2008;23(5):308–17.

Hill J, Dickson-Hall L, Grant AD, Grundy C, Black J, Kielmann K, et al. Drug-resistant tuberculosis patient care journeys in South Africa: A pilot study using routine laboratory data. International Journal of Tuberculosis and Lung Disease. 2020;24(1):83–91.

Stokes J, Man MS, Guthrie B, Mercer SW, Salisbury C, Bower P. The foundations framework for developing and reporting new models of care for multimorbidity. Ann Fam Med. 2017;15(6):570–7.

Sijm-Eeken, M, Zheng J, Peute L. Towards a Lean Process for Patient Journey Mapping - A Case Study in a Large Academic Setting. Stud Health Technol Inform. 2020;270:1071–5.

Hanson CL, Osberg M, Brown J, Durham G, Chin DP. Conducting Patient-Pathway Analysis to Inform Programming of Tuberculosis Services: Methods. Journal of Infectious Diseases. 2017;216(Suppl 7):S679–85.

Rathi P, Shringarpure K, Unnikrishnan B, Pandey A, Nair A. Patient treatment pathways of multidrug-resistant tuberculosis cases in coastal South India: Road to a drug resistant tuberculosis center. F1000Res. 2019;8:498.

Loveday M, Wallengren K, Reddy T, Besada D, Brust JCM, Voce A, et al. MDR-TB patients in KwaZulu-Natal, South Africa: Cost-effectiveness of 5 models of care. PLoS One. 2018;13(4):1–13.

Mitrani L, Roux S Le, Hill J, Loveday M, Grant AD, Kielmann K, et al. Diverse clinical and social circumstances: developing patient-centred care for DR-TB patients in South Africa L. Public Health Action. 2021;I(3):120–5.

Citro B, Soltan V, Malar J, Katlholo T, Smyth C, Sari AH, et al. Building the Evidence for a Rights-Based , People- Centered , Gender-Transformative Tuberculosis Response : An Analysis of the Stop TB Partnership Community , Rights , and Gender Tuberculosis Assessment. Health and Human Rights Journal. 2021;23(2):253–67.

Moshabela, M, Cox, H., Jassat, W., Dickson-Hall, L., Kielmann, K., Loveday M., Black, J., Mlisana, K., Nicol, M., Grant A. "We were caught sleeping": Tensions between 'control-based' and 'access-based' approaches in the formulation of a national policy to decentralise management of drug-resistant tuberculosis in South Africa. Manuscript under review, BMJ Global Health.

For peer review only

Furin J, Loveday M, Hlangu S, Dickson-hall L, Le Roux S, Nicol M, et al. “ A very humiliating illness ” : a qualitative study of patient-centered Care for Rifampicin-Resistant Tuberculosis in South Africa. 2020;1–11.

Loveday M, Wallengren K, Brust J, Roberts J, Voce A, Margot B et al. Community-based care vs. centralised hospitalisation for MDR-TB patients, KwaZulu-Natal, South Africa. Int J Tuberc Lung Dis. 2015;19(2):163–71.

Loveday M, Wallengren K, Voce A, Margot B, Reddy T, Master I, et al. Comparing early treatment outcomes of MDR-TB in decentralised and centralised settings in KwaZulu-Natal, South Africa. International Journal of Tuberculosis and Lung Disease. 2012;16(2):209–15.

Petersen E, Khamis F, Battista G, Glerup J, Marais B, Wejse C, et al. International Journal of Infectious Diseases De-isolation of patients with pulmonary tuberculosis after start of treatment — clear , unequivocal guidelines are missing. International Journal of Infectious Diseases. 2017;56:34–8.

Shringarpure KS, Isaakidis P, Sagili KD, Baxi RK, Das M, Daftary A. “When treatment is more challenging than the disease”: A qualitative study of MDR-TB patient retention. PLoS One. 2016;11(3):1–12.

Daftary A, Mondal S, Zelnick J, Friedland G, Seepamore B, Boodhram R, et al. Dynamic needs and challenges of people with drug-resistant tuberculosis and HIV in South Africa : a qualitative study. Lancet Glob Health. 2015;9(4):e479–88.

Al-mandhari A, Al-adawi S, Al-zakwani I, Al-shafaee M, Eloul L. Impact of Geographical Proximity on Health Care Seeking Behaviour in Northern Oman. 2008;8(3):310–8.

Peabody, JW, Taguiwalo, MM, Robalino, DA, Frenk J. Improving the Quality of Care in Developing Countries. In: Disease Control Priorities in Developing Countries. 2nd ed. New York: Oxford University Press; 2006.

Mangundu M, Roets L, Rensberg EJ Van, Africa S, Mangundu M. Accessibility of healthcare in rural Zimbabwe : The perspective of nurses and healthcare users. :1–7.

The Global Fund. The Global Fund. 2015 [cited 2021 Oct 20]. A Toolkit for Health Facilities Differentiated Care For HIV and Tuberculosis. Available from: https://reliefweb.int/report/world/toolkit-health-facilities-differentiated-care-hiv-and-tuberculosis

Benn J, Burnett S, Parand A, Pinto A, Iskander S, Vincent C. Studying large-scale programmes to improve patient safety in whole care systems: Challenges for research. Soc Sci Med. 2009 Dec 1;69(12):1767–76.

Harrison MB, Legare F, Graham ID, Fervers B. Adapting clinical practice guidelines to local context and assessing barriers to their use. CMAJ. 2010;182(2):78–84.

Hodkinson P, Argent A, Wallis L, Reid S, Perera R, Harrison S, et al. Pathways to care for critically Ill or injured children: A cohort study from first presentation to healthcare services through to admission to intensive care or death. PLoS One. 2016;11(1).

Das M, Mathur T, Ravi S, Meneguim AC, Iyer A, Mansoor H, et al. Challenging drug-resistant TB treatment journey for children, adolescents and their care-givers: A qualitative study. PLoS One. 2021;16(3 March):1–15.

For peer review only

Seddon JA, Hesseling AC, Godfrey-Faussett P, Schaaf HS. High treatment success in children treated for multidrug-resistant tuberculosis: An observational cohort study. Thorax. 2014;69(5):471–7.

World Health Organization. WHO | WHO End TB Strategy. World Health Organization. 2015;

World Health Organization. Integrated care models: an overview. Health Services Delivery Programme. 2016;42.

Epstein RM, Legacy GES. The Biopsychosocial Model 25 Years Later : 2004;576–82.

Babiker A, Husseini M El, Nemri A Al, Frayh A Al, Juryyan N Al, Faki MO, et al. Current Opinion Health care professional development : Working as a team to improve patient care. 2014;14(2):9–16.

Leeper SC, Montague BT, Friedman JF, Flanigan TP. Lessons learned from family-centred models of treatment for children living with HIV: Current approaches and future directions. J Int AIDS Soc. 2010;13(SUPPL. 2).

Le Roux SR, Jassat W, Dickson L, Mitrani L, Cox H, Mlisana K, et al. The role of emergent champions in policy implementation for decentralised drug-resistant tuberculosis care in South Africa. BMJ Glob Health. 2022 Dec 9;7(12).

Le Roux, Sacha, Jassat, Waasila, Dickson-hall, Lindy, Mitrani, Leila, Cox, Helen, Milsana K, et al. The role of healthcare champions in policy implementation for decentralised drug-resistant tuberculosis care in South Africa. Manuscr under Rev BMJ Global Health.

Chin DP, Hanson CL. Finding the Missing Tuberculosis Patients. Journal of Infectious Diseases. 2017;216:S675–8.

Chin DP, Hanson CL. Finding the Missing Tuberculosis Patients. Journal of Infectious Diseases. 2017;216(Suppl 7):S675–8.

Vaikuntam BP, Middleton JW, Mcelduff P, Pearse J, Walsh J, Cameron ID, et al. Assessing the impact of care pathways on potentially preventable complications and costs for spinal trauma patients : protocol for a data linkage study using cohort study and administrative data. 2018;10–4.

Sharwood LN, Whyatt D, Vaikuntam BP, Cheng CL, Noonan VK, Joseph AP, et al. A geospatial examination of specialist care accessibility and impact on health outcomes for patients with acute traumatic spinal cord injury in New South Wales , Australia : a population record linkage study. 2021;4:1–13.

Moscibrodzki P, Parkinson, S, Ferry R, Nwaneri, N, Thies W. Mapping the technology landscape of national TB programs. Geneva; 2021.

World Health Organization; Monitoring the building blocks of health systems : A handbook of indiators and their measurement strategies. Geneva; 2010.

World Health Organization, Organisation for Economic Co-operation and Development TWB. Delivering quality health services: A global imperative for universal health coverage. Geneva; 2018.

Ramsden R, Davies S, Richard MPH, Ba C, Service H, Shannon M, et al. Collaborative care : Primary health workforce and service delivery in Western New South Wales — A case study. 2021;(July):768–78.

Taber JM, Ph D, Leyva B, Persoskie A, Ph D. Why do People Avoid Medical Care ? A Qualitative Study Using National Data. 2008;18–24.

For peer review only

For peer review only - http://bmjopen.bmj.com/site/about/guidelines.xhtml

Kruk ME, Gage AD, Arsenault C, Jordan K, Leslie HH, Roder-dewan S, et al. The Lancet Global Health Commission High-quality health systems in the Sustainable Development Goals era : time for a revolution. 2018;6(November):1196–252.

World Health Organization. Action on the Social Determinants of Health: Learning from Previous Experiences. Social Determinants of Health Discussion Paper 1. 2010.

Dookie S, Singh S. Primary health services at district level in South Africa: a critique of the primary health care approach. BMC Fam Pract. 2012;13:67.

World Health Organization. Global Tuberculosis Report 2015. Geneva; 2015.

For peer review only

760

Pattern name	Fan	Chicken foot	Hub & spoke	Triangle	Line	Dot
**Level of decentralisation**	Highly centralised care	Care decentralised to district level with high reliance on COE	Care decentralised to district level	Multi-level / multi-facility decentralised care	Community based, ambulatory care	Community based, ambulatory care
**Description of care delivery**	Hospital based care with long admissions, far from home.	Hospital based care with long admissions, some care closer to home.	Hospital based care with long admissions, closer to home.	Flexible care, coordinated by specialist doctors.	Home based care, with short admissions if required.	Home based, outpatient care.
**Diagnosis**	At clinic or general☐ hospital.	At clinic or general☐ hospital	At clinic or general hospital	At clinic or general☐ hospital	At clinic	At clinic
**Treatment initiation**	At provincial COE	At specialist TB hospital, but with frequent change to regimen at COE.	At specialist TB hospital	At specialist TB hospital or by specialist TB outreach service.	At MDR/RR-TB clinic or district hospital MDR/RR-TB clinic	At the diagnosing clinic
**Co-ordination of care and follow up**	Specialist TB doctors at the COE	Specialist TB doctors at the COE and TB doctors at the decentralised site.	TB doctors at the specialised site	Specialist physician or TB physician on site or through telemedicine.	TB nurses at clinics. Sometimes assisted by a generalist doctor.	TB nurses at clinics. Sometimes assisted in person/telephonically by generalist doctor.
**Hospitalisation**	At COE, frequently for long periods.	At specialist TB hospital or COE, frequently for long periods.	At specialist TB hospital for long periods.	Depending on patient's medical and social circumstances.	Depending on patient's medical and social circumstances.	No admission.
**Number of participants**	N=41	N=19	N=72	N=30	N=12	N=17

For peer review only - http://bmjopen.bmj.com/site/about/guidelines.xhtml

	(urban n=25, rural n =16)	(urban n=5, rural n=14)	(urban n=29, rural n=43)	(urban n=13, rural n=17)	(urban n=4, rural n=8)	(urban n=10, rural n=7)
**Median distance from home to diagnosing site****	10km (n=36)	11.4km (n=17)	14.6km (n=66)	10.6km (n=24)	5.4km (n=6)	0.9km (n=14)
**Median distance from home to initiating site****	44km (n=36)	38km (n=17)	41km (n=66)	26km (n=24)	6km (n=6)	2km (n=14)
**Median distance travelled between facilities**	102 km	188km	51km	76km	8km	0km
**Median time to treatment initiation*****	35 days	13 days	14 days	7 days	0 days	1 day
**Median (range) number of trips******	6 (1-7)	5 (2-12)	6 (1-17)	8 (2-16)	6 (1-21)	8 (6-22)
**Median duration of hospital admission**	79 days (IQR=105) Range 0 - 240	103 days (IQR=91) Range 20 - 197	81 days (IQR=124 Range 0 - 281	40 days (IQR=57) Range 0 - 175	12 days (IQR=24) Range 0 - 38	0 days (IQR=0) Range:0 - 0
**Vital status 6 months following MDR/RR-TB lab result**	Deceased 20% (8)	Decreased 11% (2)	Deceased 25% (18)	Deceased 20% (6)	Deceased 33% (4)	Deceased - Nil

For peer review only

**Treatment status at 24 months following MDR/RR-TB lab result**	Cured / Completed treatment 45% (19)<br>Still on treatment 21% (9)<br>*(favourable 68%)*<br>Deceased 24% (10)<br>Lost to follow-up 5% (2)<br>Treatment failure Nil<br>Unknown 3% (1)	Cured / Completed treatment 33% (6)<br>Still on treatment 16.5% (3)<br>*(favourable 47%)*<br>Deceased 16.5% (3)<br>Lost to follow-up 37% (7)<br>Treatment failure Nil<br>Unknown Nil	Cured / Completed treatment 41% (29)<br>Still on treatment 21% (15)<br>*(favourable 61%)*<br>Deceased 26% (19)<br>Lost to follow-up 9% (6)<br>Treatment failure 2% (1)<br>Unknown 3% (2)	Cured / Completed treatment 53% (16)<br>Still on treatment 17% (5)<br>*(favourable 70%)*<br>Deceased 20% (6)<br>Lost to follow-up 10% (3)<br>Treatment failure Nil<br>Unknown Nil	Cured / Completed treatment 17% (2)<br>Still on treatment 42% (5)<br>*(favourable 58%)*<br>Deceased 42% (5)<br>Lost to follow-up Nil<br>Treatment failure Nil<br>Unknown Nil	Cured / Completed treatment 94% (16)<br>Still on treatment Nil<br>*(favourable 94%)*<br>Deceased Nil<br>Lost to follow-up 6% (1)<br>Treatment failure Nil<br>Unknown Nil

* n = number of participants ** distance travelled calculated as straight-line distance as calculated from the patient's home to the health care site. *** Days from MDR/RR-TB diagnosis to treatment initiation

**** Number of trips undertaken to health care sites during first 9 months of treatment. ☐General hospital indicates either a district or regional level hospital. COE – TB Centre of Excellence

<u>Table 1: Dominant geospatial movement patterns observed within study districts, together with key features of organisation of care within these patterns, descriptive statistics of patient journeys and treatment status after 24 months.</u>

peer review only

For peer review only - http://bmjopen.bmj.com/site/about/guidelines.xhtml

Province	Rural or urban	Districts	A<br>Fan	B<br>Chicken foot	C<br>Hub & Spoke	D<br>Triangle	E<br>Line	F<br>Dot
**KwaZulu -Natal**	Urban	Ethekwini	4	3	5	1		1
		Uthungulu		4	2	7		2
	Rural	Ilembe*	2		8	5		
		Umkanyakude		2	7	6		
		Uthukela		6	8	1		
**Western Cape**	Urban	City of Cape Town	5	2			3	5
		Eden			12	2		1
	Rural	Cape Winelands			8	2	2	3
		West Coast	1		9		2	2
**Eastern Cape**	Urban	Buffalo City	14					
		Nelson Mandela Bay			10	3	1	1
	Rural	OR Tambo	8		1	1	4	1
		Sarah Baartman	7	2	2	2		1
		**TOTAL (n=191):**	**41**	**19**	**72**	**30**	**12**	**17**

iLembe* – undergoing rural to urban transition

Numbers in cells refer to number of individual patient pathways observed for each organisational model within the district

Table 2: The distribution of organisational models by study district.

For peer review only

For peer review only - http://bmjopen.bmj.com/site/about/guidelines.xhtml

PATIENT FEATURES	Category	Fan *n=41	Chicken foot n=19	Hub & spoke n=72	Triangle n=30	Line n=12	Dot n=17	Total n=191
Age category (yrs)	0-19 yrs	3 (7)	1 (5)	3 (4)	2 (7)	0	0	9 (5)
	20-59 yrs	38 (93)	18 (95)	65 (90)	27 (90)	12 (100)	17 (100)	177 (92)
	≥60 yrs	0	0	4 (6)	1 (3)	0	0	5 (3)
Not receiving income		30 (73)	14 (74)	49 (68)	18 (60)	4 (33)	9 (53)	124 (65)
HIV positive		29 (71)	16 (84)	50 (70)	18 (60)	11 (92)	7 (41)	131 (69)
Baseline CD4 <200 c/ul**	CD4 <200 c/ul	14 (48)	6 (38)	15 (30)	5 (26)	5 (45)	2 (29)	47 (35)
	CD4≥200 c/ul	9 (31)	5 (31)	17 (34)	6 (33)	3 (27)	3 (43)	43 (33)
	Unreported CD4	6 (21)	5 (31)	18 (36)	7 (39)	3 (27)	2 (29)	41 (32)
Previous TB history	DS-TB any episode	25 (61)	14 (74)	49 (68)	19 (63)	6 (50)	8 (47)	121 (63)
	MDR/RR-TB 1 episode	3 (7)	4 (21)	6 (8)	3 (10)	3 (25)	1 (6)	20 (10)
	MDR/RR-TB ≥2 episodes	5 (12)	1 (5)	9 (13)	1 (3)	0	0	16 (9)
Baseline BMI <18.5 kg/m2	≤18.5 kg/m2	10 (24)	7 (37)	25 (35)	7 (23)	5 (42)	4 (24)	58 (49)
	≥18.6 kg/m2	19 (47)	8 (42)	26 (36)	11 (34)	0 (0)	9 (52)	74
	Unreported BMI	12 (29)	4 (21)	21 (29)	12 (40)	7 (58)	4 (24)	60 (51)
Comorbidities per person (excluding HIV)	1-3 comorbidities/pt	4 (10)	4 (21)	15 (20)	10 (33)	2 (17)	2 (12)	37 (19)
	> 3 comorbidities/pt	0	0	2 (3)	1 (3)	4 (33)	2 (12)	9 (5)
2nd line TB drug resistance	MDR/RR-TB	22 (53)	11 (58)	46 (64)	22 (73)	9 (76)	12 (70)	122 (64)
	Pre – XDR	11 (27)	6 (32)	8 (11)	4 (13)	1 (8)	1 (6)	31 (16)
	XDR	6 (15)	1 (5)	8 (11)	2 (7)	1 (8)	1 (6)	19 (10)
	Unknown resistance	2 (5)	1 (5)	10 (14)	2 (7)	1 (8)	3 (18)	19 (10)

*Number of patients **Within 3 months of diagnosis % refer to proportion of patients within each pattern

For peer review only - http://bmjopen.bmj.com/site/about/guidelines.xhtml

774 Table 3: Characteristics of the study participants n=191 within each organisational pattern
775 that may influence the degree of decentralisation achieved.

For peer review only

PATTERN NAME	Fan	Chicken foot	Hub & spoke	Triangle	Line	Dot
**Advantages of the organisational model for people with MDR/RR-TB**	Access to multidisciplinary team with experienced clinicians, drug availability.<br><br>Access to acute tertiary (ICU, dialysis) and chronic tertiary care services (nephrology, cardiology, neurology).	People with less complex illness are able to be cared for in their districts.<br><br>Ready referral pathway back to the centralised site for people with complex illness.	People are cared for in their districts, closer to home .<br><br>Specialist TB hospitals may be better designed for long-term care than COEs. Staff are more familiar with individual people and their conditions and contexts, compared to COE.	People able to access care at different facilities, depending on preference.<br><br>As a collective, the facilities have multidisciplinary resources.<br><br>Expert conductor has the ability to navigate care and advocate for holistic care. Oversight for people with complex conditions.	Care close to home.<br><br>Initial period of supervised inpatient care may provide nutrition support, side effect monitoring, linkage to financial grant support & patient education.	Care very close to home<br><br>Increased focus on community, patient- and family-centred care, with staff embedded within the community<br><br>Other decentralised services, eg, HIV care, family planning, are easily accessible.
**Advantages of the organisational model for the health system**	Logistically simple to compile statistics & monitor people with MDR/RR-TB and train specialised staff.<br><br>Rapid response to change in diagnostic or drug regimens.<br><br>Centralization of expensive drug storage & control – minimise waste, expiry or	Reduces bed pressure on centralised sites, whilst maintaining strong clinical governance of people with complex illness.<br><br>Less reliance on one facility per province (redundancy).	Strong connections between specialist TB hospital and clinics facilitates patient monitoring and surveillance.<br><br>The district node taking a high degree of responsibility promotes accountability.<br><br>Early treatment modification by TB specialists at the	Oversight from an expert conductor facilitates rapid adjustment of patient care, reducing hospitalization and optimising resource usage.<br><br>Leverage of resources by senior clinicians.<br><br>Flexibility around staffing	Outpatient review within the community, with option for short hospital admission for treatment optimisation and stabilisation.<br><br>Reduces patient transport (infection transmission, expense) and promotes family support, whilst minimising the need to provide services and drugs to	Rapid diagnosis and treatment initiation.<br><br>Reduced requirement for patient transport.<br><br>Patient trust in local staff promotes adherence and treatment completion.

For peer review only - http://bmjopen.bmj.com/site/about/guidelines.xhtml

	shrinkage.		district level reduces admissions.	allows system to buffer more easily.	every clinic.	Support from decentralised HIV programmes.
**Challenges of the organisational model for people with MDR/RR-TB**	Treatment delay, long admission times, hospital acquired infection, poor family centredness, loss of patient income.<br><br>Community stigma of being at a CoE for 'strong' TB. Disconnect from community chronic care services.	People with MDR/RR-TB need to adjust to care in dfferent hospitals.<br><br>Fragmentation of care<br><br>Travel between sites.<br><br>Moderate to long admission times.	District sites may have reduced support from COEs. This may result in difficulty referring people with MDR/RR-TB requiring more complex care.<br><br>Moderate to long admission times.	Care may be fragmented between the sites & people may become lost to follow-up between sites.<br><br>Requires support from the province (travel, information technology services, training) which may not be consistent.	Sites may have reduced support from COEs. This may result in difficulty referring people requiring more complex care.<br><br>Multidisciplinary team not always available. Complications may go undetected or there may be a delay in referring for higher levels of care.	Sites may have reduced support from COEs. This may result in difficulty referring people requiring more complex care.<br><br>Multidisciplinary team less available. Complications may go undetected or delay in referral for higher-level care.
**Challenges of the organisational model for the health system**	Reliance on a single centre - lack of redundancy.<br><br>Skill set 'owned' by a few individuals at the COE's. Other HCWs see themselves as unable to care for MDR/RR-TB reducing willingness to care for these people.	Over-reliance on COE, with decentralised site poorly empowered to manage people with MDR/RR-TB .<br><br>Providing safe patient transport.	Depending on the geographical size of the province, many people with MDR/RR-TB still travel far distances to visit the TB service or be admitted to hospital.	Reliance on retention of individual specialist physicians to maintain the program.<br><br>Tracking patient progress through the system.<br><br>Providing safe transport.	Monitoring & supporting many MDR-TB sites. Providing sites with laboratory & IT infrastructure, drugs, trained staff and supervision.<br><br>Ensuring quality of care provision and timely capturing of accurate data.	Monitoring & supporting many MDR-TB sites. Providing sites with laboratory & IT infrastructure, drugs, trained staff, and supervision.<br><br>Ensuring quality of care provision and timely capturing of accurate data.

For peer review only - http://bmjopen.bmj.com/site/about/guidelines.xhtml

Table 4. Advantages and challenges associated with each organisational care pattern

For peer review only

For peer review only - http://bmjopen.bmj.com/site/about/guidelines.xhtml

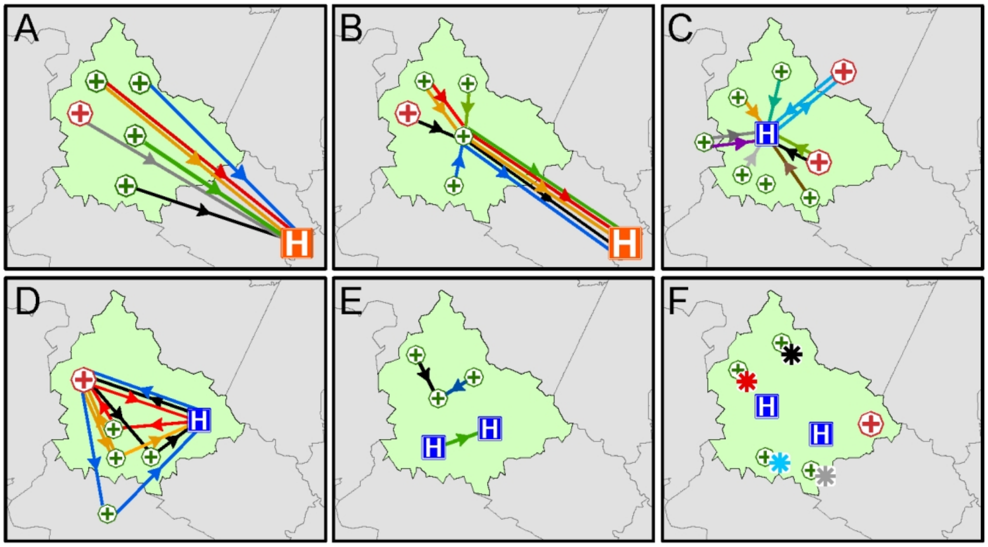

Figure 1. Examples of the dominant geospatial movement patterns annotated on a stylised study district. Each coloured line depicts a single participant travelling in a pathway between the specific levels of health care facilities found in that model. Arrows represent direction of participant movement direction between health care facilities. Stars represent participants who only visited a single facility across their diagnostic and treatment pathway. A: 'Fan'; B: 'Chicken Foot'; C;' Hub and Spoke'; D: 'Triangle'; E: 'Line'; F: 'Dot'.

210x148mm (200 x 200 DPI)

Supplementary material: A

Additional information related to COREQ (Consolidated criteria for Reporting Qualitative research) Checklist

Domain 1: Research team & reflexivity

LD, JH, SLR and LM conducted interviews. The researchers were a team of clinicians and social science degree majors qualified in the fields of medicine, biology and teaching. At the time of the study all were full time researchers on the project. Three were female and one was male. Three were South African and one was Australian. Three had Masters degrees and one had a Bachelor's degree. LD had been employed as a clinician in the DR-TB, HIV and family medicine programs in KZN and the Western Cape and had been previously working on a DR-TB linkage to care study. JH had been employed as a clinician in the DR-TB and infectious diseases domain at Papua New Guinea. SLR had been teaching senior high school students and working in a health care call centre and medical practice previously. LM had been completing a master's degree in biology involving geo-informatics. LD and JH had been investigators in previous DR-TB studies. There was no formal relationship established prior to the study, except some familiarity between the local DR-TB clinicians and LD. The participants were aware of research aims and objectives and the names of the researchers and investigators. The participants were aware that the researchers were interested in health systems around DR-TB disease in SA.

Domain 2: Study design

Questions and guides were piloted with a smaller group of local DR-TB staff at a non-study facility and then provided to participants by the interviewers, together with patient journey maps obtained from that region to serve as probes for some questions. Audio recordings were obtained, except in the case of a correctional services facility where recording was not permitted. A single round of interviews took place at the work place during tea or lunch breaks. Field notes were made during and then more comprehensively after the interview. The duration of the interviews ranged from 30-50 minutes, and depended on the operational work time constraints and the degree of insight and knowledge held and shared by the participant. Data saturation was discussed before ending interviews. Transcripts were not returned for comment or correction.

Domain 3: Analysis and findings

SLR and LD reviewed the interviews and coded the data, using a simple, unstructured coding scheme to extract information on the implications of the patterns of care for patients or the health care system . Participants did not give feedback on the data, except at an end-of-study feedback session with the Departments of Health, which was not used to inform this manuscript. Participant quotations were not presented. There was consistency between the data presented and the findings. Major themes were clearly presented in the findings. The nature of the patient pathway analysis allowed for interrogation of both major themes (common reasons for patient movement) as well as minor themes less common reasons for patient movement).

For peer review only - http://bmjopen.bmj.com/site/about/guidelines.xhtml

Supplementary information: B

		Rural or urban	Demographic and health care descriptors					Poverty indices				Death and disease profile						
			Population count	Population density / km²	Number of clinics	Number of public hospitals	Provincial spend on health / person / year (ZAR)*	Population below lower poverty line	Unemployed	Informal shack dwellings	Illiteracy	Life expectancy (yrs)	% of deaths due to infectious diseases	Leading cause of natural death	HIV / TB coinfection	TB incidence / 100 000 in 2015	Number of MDR/RR-TB cases in 2017**	
																	XDR	MDR
PROVINCE	KWAZULU-NATAL						R3 527	44%	28%		15%	57-64			69%	685	115	1687
DISTRICT	eThekwini (1)***	City (metro)	3 702 231	1502	31	18		41%	19%	13%	17%		16%	Heart disease		698	85	1063
	uThungulu (2)	City (small)	971 135	88	83	9		72%	34%	3%	12%		21%	TB		859	7	242
	iLembe (3)	Rural (small metro)	657 612	185	36	8		55%	31%	9%	15%		24%	TB		801	10	151
	uMkhanyakude (4)	Rural district	689 090	61	86	5		80%	31%	15%	25%		27%	HIV/ AIDS		577	6	146
	uThukela(5)	Rural district	706 588	63	49	4		63%	29%	1%	10%		24%	TB		533	7	85
PROVINCE	WESTERN CAPE						R3 867	32%	20%		7%	66-71			47%	681	86	1140
DISTRICT	City of Cape Town (6)	City (metro)	4 208 000	1530	130	18		31%	22%	11%	2%		14%	Diabetes		596	59	757
	Eden (7)	City (metro)	611 279	25	209	7		33%	27%	8%	12%		17%	Diabetes		809	5	100
	Cape Winelands (8)	Rural district	917 462	40	134	10		34%	17%	7%			18%	Diabetes		880	14	170
	West Coast (9)	Rural district	436 403	15	180	7		30%	19%	8%	13%		16%	Heart disease		837	8	113
PROVINCE	EASTERN CAPE						R3 046	56%	47%		18%	60-67			56%	692	281	1151
DISTRICT	Buffalo City (7)	City (metro)	893 157	300	103	10		41%	35%	25%	12%		21%	TB		743	77	314
	Nelson Mandela Bay(8)	City (metro)	1 263 051	615	68	12		51%	63%	7%	1%		20%	Diabetes		938	150	471
	OR Tambo (5)	Rural district	1 457 384	108	147	16		67%	39%	1%	14%		20%	TB		571	22	220
	Sarah Baartman (10)	Rural district	520 480	8	89	15		41%	30%	9%	13%		20%	TB		1022	32	146

Table S1. Description of key health care and demographic features of the study districts

* ZAR = South African Rands (1 US dollar is approximately 17 ZAR)

** Data from National Institute of Communicable Diseases

***Reference numbers for data sources are given following each district name

For peer review only - http://bmjopen.bmj.com/site/about/guidelines.xhtml

Disease	Definition	Programmatic treatment for MDR/RR-TB in South Africa
DR-TB	Drug resistant TB<br>(Resistance to any TB drug)	Second-line treatment for 18–24 months, with a combination including a combination selection with several of:<br><br>• Fluoroquinolones, e.g.: ofloxacin, moxifloxacin, levofloxacin<br>• Second-line injectable drugs, e.g.: amikacin, kanamycin, capreomycin<br>• Other available drug, e.g.: ethionamide, terizidone, para-aminosalicyclic acid<br>• Repurposed drugs, e.g.: linezolid, clofazimine<br>• New drugs recently available, e.g.: bedaquiline, delamanid
MDR-TB	Multi drug resistant TB<br>(Resistance to isoniazid and rifampicin)	
RR-TB	Rifampicin resistant TB<br>(Resistant to at least rifampicin, regardless of resistance to other drugs)	
PreXDR-TB	Pre-Extensively drug resistant TB<br>(MDR-TB with resistance to either a fluoroquinolone OR a second-line injectable TB drug)	
XDR-TB	Extensively drug-resistant TB<br>(MDR-TB with resistance to both a fluoroquinolone AND a second-line injectable TB drug)	
Completed treatment	Treatment completed as recommended by the national policy without evidence of failure *but no record that* three or more consecutive cultures ≥30 days apart are negative after the intensive phase.	
Cured	Treatment completed as recommended by the national policy without evidence of failure *and* three or more consecutive cultures ≥30 days apart are negative after the intensive phase.	

Table S2. Definitions and treatment for drug-resistant TB at the time of the study

For peer review only

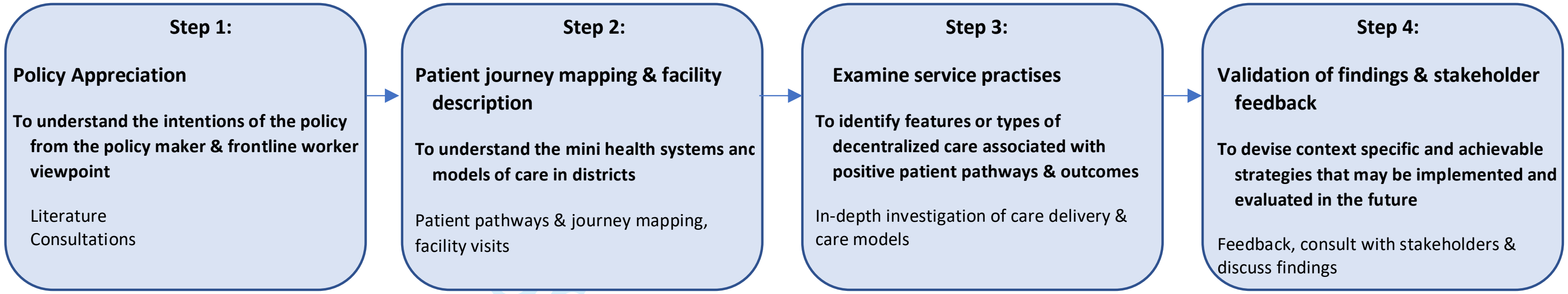

Supplementary Figure 1. The overarching research project, within which this sub-study was nested. This analysis relates to Step 2 and Step 3 of the project.

For peer review only - http://bmjopen.bmj.com/site/about/guidelines.xhtml

Supplementary Figure 2: South African map showing thirteen districts selected for study in three provinces. Districts are shaded according to Province (shaded darker green in Western Cape, lighter green in Eastern Cape and orange in KwaZulu-Natal).

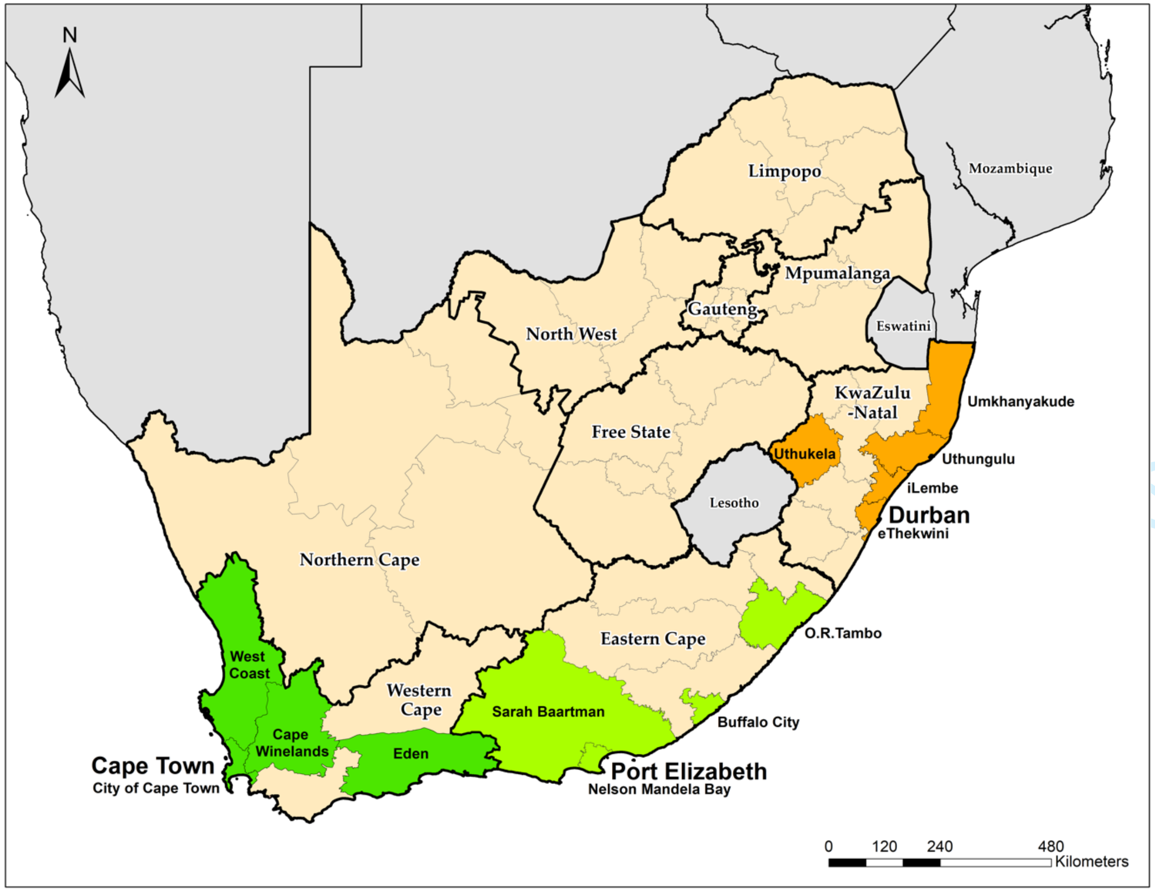

References:

1. National Department of Cooperative Governance & Traditional Affairs South Africa. Profile and Analysis : District Developement Model. 2020. p. 31 Ethekwini Metropolitan KZN. Available from: https://www.cogta.gov.za/ddm/wp-content/uploads/2020/07/Metro-Profile_Ethekwini.pdf

2. National Department of Cooperative Governance & Traditional Affairs South Africa. King Cetshwayo District. 2020.

3. National Department of Cooperative Governance & Traditional Affairs South Africa. Ilembe District Municipality KZN [Internet]. 2020. p. 39. Available from: https://www.cogta.gov.za/ddm/wp-content/uploads/2020/11/Ilembe-September2020.pdf

4. National Department of Cooperative Governance & Traditional Affairs South Africa. Umkhanyakude District Municipality [Internet]. 2020 [cited 2001 Feb 20]. p. 1–24. Available from: https://www.cogta.gov.za/ddm/wp-content/uploads/2020/07/Umkhanyakude-DM-Final-JUNE-2020.pdf

5. National Department of Cooperative Governance & Traditional Affairs South Africa. Uthukela District Municipality EC [Internet]. 2000. p. 34. Available from: https://www.cogta.gov.za/ddm/wp-content/uploads/2020/11/Uthukela-October-2020.pdf

6. National Department of Cooperative Governance & Traditional Affairs. City of Cape town Metropolitan WC. 2020.

7. National Department of Cooperative Governance & Traditional Affairs South Africa. Buffalo City Metro EC [Internet]. 2000. p. 32. Available from: https://www.cogta.gov.za/ddm/wp-content/uploads/2020/08/DistrictProfile_BUFFALOCITY07072020-1.pdf

8. National Department of Cooperative Governance & Traditional Affairs South Africa. NMBM Metro EC [Internet]. 2000. p. 1–30. Available from: https://www.cogta.gov.za/ddm/wp-content/uploads/2020/11/Nelson-Mandela-Bay-Metro-October-2020-1.pdf

9. National Department of Cooperative Governance & Traditional Affairs South Africa. West Coast District Municipality WC [Internet]. 2000 [cited 2022 Jan 20]. Available from: https://www.cogta.gov.za/ddm/wp-content/uploads/2020/08/DistrictProfile_WESTCOAST23072020-002.pdf

10. National Department of Cooperative Governance & Traditional Affairs South Africa. Sarah Baartman District Municipality GAU [Internet]. 2000. p. 43. Available from: https://www.cogta.gov.za/ddm/wp-content/uploads/2020/08/DistrictProfile_SarahBaartman01072020.pdf

For peer review only - http://bmjopen.bmj.com/site/about/guidelines.xhtml

COREQ (COnsolidated criteria for REporting Qualitative research) Checklist

A checklist of items that should be included in reports of qualitative research. You must report the page number in your manuscript where you consider each of the items listed in this checklist. If you have not included this information, either revise your manuscript accordingly before submitting or note N/A.

Topic	Item No.	Guide Questions/Description	Reported on Page No.
**Domain 1: Research team and reflexivity**			
Personal characteristics			
Interviewer/facilitator	1	Which author/s conducted the interview or focus group?	
Credentials	2	What were the researcher's credentials? E.g. PhD, MD	
Occupation	3	What was their occupation at the time of the study?	
Gender	4	Was the researcher male or female?	
Experience and training	5	What experience or training did the researcher have?	
Relationship with participants			
Relationship established	6	Was a relationship established prior to study commencement?	
Participant knowledge of the interviewer	7	What did the participants know about the researcher? e.g. personal goals, reasons for doing the research	
Interviewer characteristics	8	What characteristics were reported about the inter viewer/facilitator? e.g. Bias, assumptions, reasons and interests in the research topic	
**Domain 2: Study design**			
Theoretical framework			
Methodological orientation and Theory	9	What methodological orientation was stated to underpin the study? e.g. grounded theory, discourse analysis, ethnography, phenomenology, content analysis	
Participant selection			
Sampling	10	How were participants selected? e.g. purposive, convenience, consecutive, snowball	
Method of approach	11	How were participants approached? e.g. face-to-face, telephone, mail, email	
Sample size	12	How many participants were in the study?	
Non-participation	13	How many people refused to participate or dropped out? Reasons?	
Setting			
Setting of data collection	14	Where was the data collected? e.g. home, clinic, workplace	
Presence of non-participants	15	Was anyone else present besides the participants and researchers?	
Description of sample	16	What are the important characteristics of the sample? e.g. demographic data, date	
Data collection			
Interview guide	17	Were questions, prompts, guides provided by the authors? Was it pilot tested?	
Repeat interviews	18	Were repeat inter views carried out? If yes, how many?	
Audio/visual recording	19	Did the research use audio or visual recording to collect the data?	
Field notes	20	Were field notes made during and/or after the inter view or focus group?	
Duration	21	What was the duration of the inter views or focus group?	
Data saturation	22	Was data saturation discussed?	
Transcripts returned	23	Were transcripts returned to participants for comment and/or	

For peer review only

Topic	Item No.	Guide Questions/Description	Reported on Page No.
		correction?	
**Domain 3: analysis and findings**			
Data analysis			
Number of data coders	24	How many data coders coded the data?	
Description of the coding tree	25	Did authors provide a description of the coding tree?	
Derivation of themes	26	Were themes identified in advance or derived from the data?	
Software	27	What software, if applicable, was used to manage the data?	
Participant checking	28	Did participants provide feedback on the findings?	
Reporting			
Quotations presented	29	Were participant quotations presented to illustrate the themes/findings? Was each quotation identified? e.g. participant number	
Data and findings consistent	30	Was there consistency between the data presented and the findings?	
Clarity of major themes	31	Were major themes clearly presented in the findings?	
Clarity of minor themes	32	Is there a description of diverse cases or discussion of minor themes?	

Developed from: Tong A, Sainsbury P, Craig J. Consolidated criteria for reporting qualitative research (COREQ): a 32-item checklist for interviews and focus groups. *International Journal for Quality in Health Care*. 2007. Volume 19, Number 6: pp. 349 – 357

Once you have completed this checklist, please save a copy and upload it as part of your submission. DO NOT include this checklist as part of the main manuscript document. It must be uploaded as a separate file.

For peer review only
